# C–H Activation of Pyridines by Boryl Pincer Complexes: Elucidation of Boryl-Directed C–H Oxidative Addition to Ir and Discovery of Transition Metal-Assisted Reductive Elimination from Boron at Rh

**DOI:** 10.1021/jacs.4c12143

**Published:** 2024-10-30

**Authors:** Vinh T. Nguyen, R. Noah Sladek, Yihan Cao, Nattamai Bhuvanesh, Jia Zhou, Oleg V. Ozerov

**Affiliations:** † Department of Chemistry, Texas A&M University, College Station, Texas 77842, United States; ‡ State Key Lab of Urban Water Resource and Environment, School of Science, Harbin Institute of Technology, Shenzhen 518055, China

## Abstract

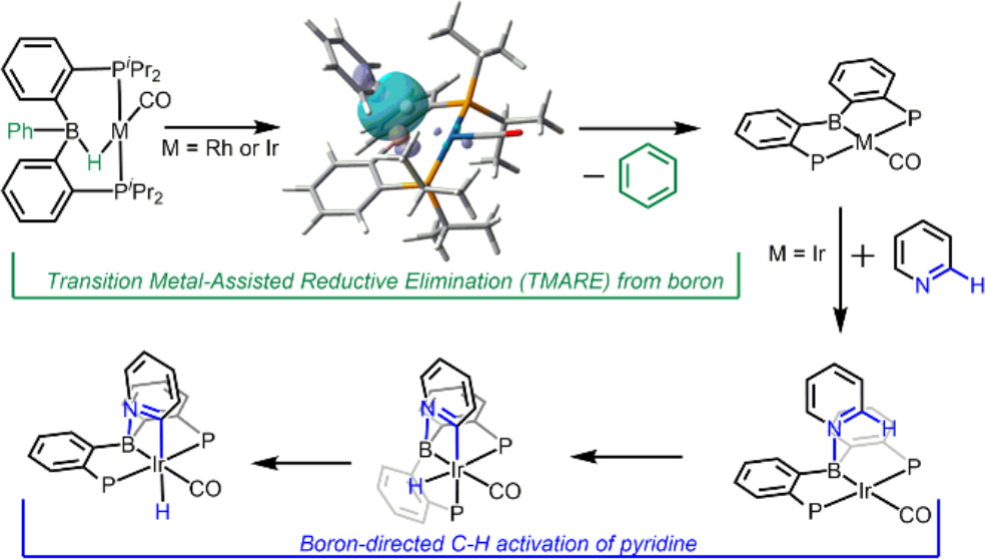

Experimental and theoretical techniques were used to investigate the mechanism of pyridine C–H activation by diarylboryl/bis(phosphine) PBP pincer complexes of Ir. The critical intermediate (PBP)IrCO (**4**) contains a three-coordinate, Ir-bound boron that retains Lewis acidity in the perpendicular direction. Coordination of pyridine to this boron center in **4** leads to fast insertion of Ir into the 2-CH bond of pyridine, providing a different topology of direction than the conventional directed C–H activation where both the directing group coordination and C–H activation happen at the same metal center. Beyond this critical sequence, the system possesses significant complexity in terms of possible isomers and pathways, which have been thoroughly explored. Kinetic and thermodynamic preferences for the activation of differently substituted pyridines were also investigated. In experimental work, the key intermediate **4** is accessed via elimination of benzene from a phenyl/hydride containing precursor (PB^Ph^P)IrHCO (**3**). Density functional theory (DFT) investigations of the mechanism of benzene loss from **3** revealed the possibility of a genuinely new type of mechanism, whereby the Ph–H bond is made in a concerted process that is best described as C–H reductive elimination from boron, assisted by the transition metal (TMARE). For Ir, this pathway was predicted to be competitive with the more conventional pathways involving C–H reductive elimination from Ir, but still higher in energy barrier. However, for the Rh analog **3-Rh**, TMARE was calculated to be the preferred pathway for benzene loss and this prediction was experimentally corroborated through the study of reaction rates and the kinetic isotope effect.

## Introduction

**Figure 1:**
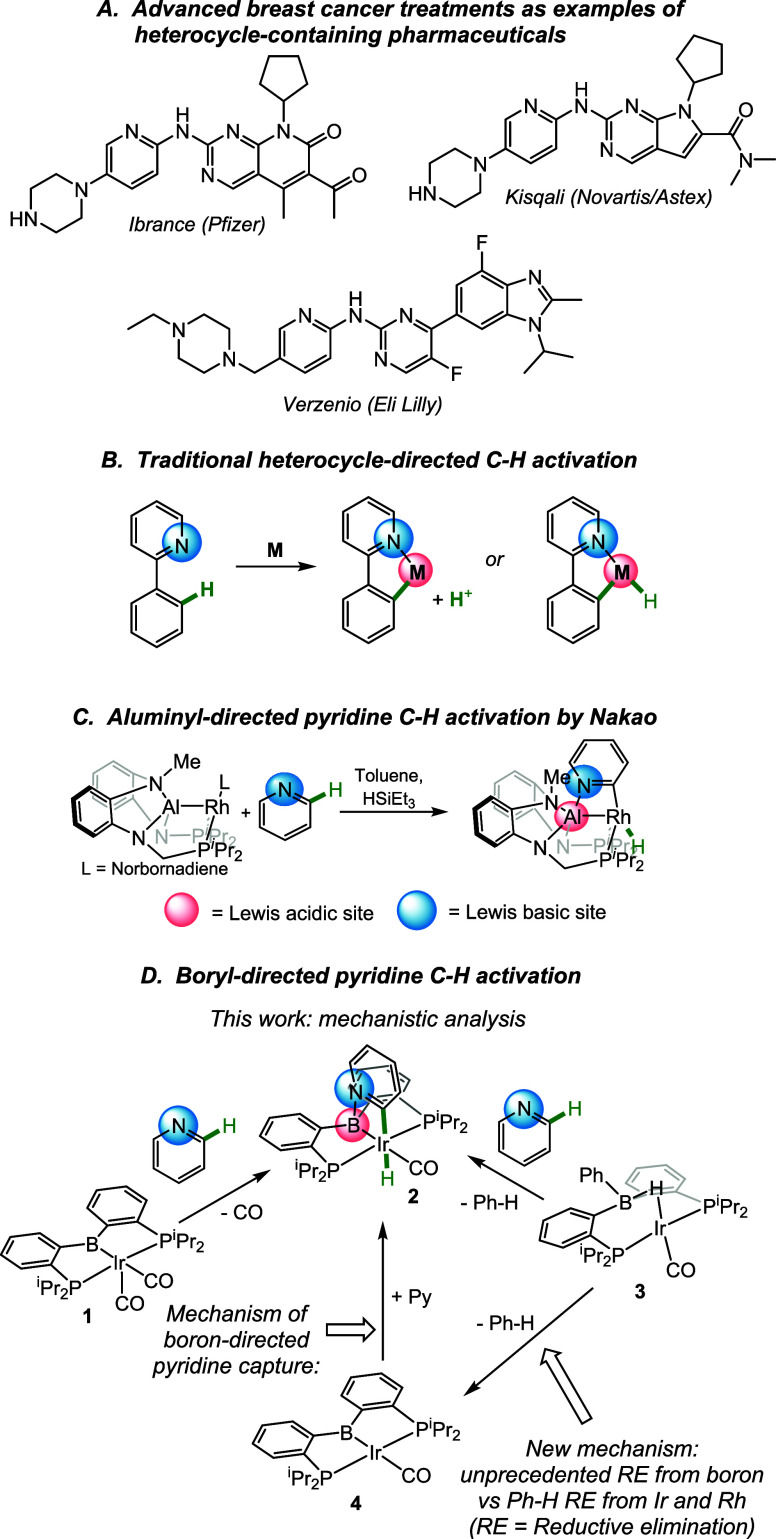
Examples of important heterocycle-containing pharmaceuticals and traditional vs main-group directed C–H activation of heterocycles.

C–H activation and functionalization of pyridines, azines, and other nitrogenous heterocycles is a broadly important challenge,^1−3^ especially relevant in the synthesis of complex molecules.^4^ A 2014 analysis noted that 59% of the FDA-approved small-molecule drugs contained a nitrogen heterocycle, with 9% containing an aromatic six-membered heterocycle.^5^ Of the 40 best-selling small-molecule pharmaceuticals in 2023 (as compiled by the Njardarson group),^6^ 14 contain an aromatic six-membered nitrogenous heterocycle. Three advanced breast cancer treatments Ibrance, Kisqali, and Verzenio (combined sales of $9.5B) are shown in Figure 1A as representative examples of state-of-the-art heterocycle-containing drugs.

The coordinating ability of the nitrogen in pyridine (and other heterocycles) toward transition metals can be both a boon and an obstacle. Large excess of a heterocyclic substrate could effectively block the coordination sites needed for C–H activation and catalysis; this often requires *ortho*-substituted pyridine substrates to diminish their binding ability.^7^ On the other hand, coordination of the nitrogen to the transition metal can be used to direct it toward a specific C–H bond in the substrate. This is now a very common approach to directed C–H functionalization (Figure 1B),^8−13^ and it typically results in the activation of C–H bonds outside of the pyridine/heterocycle ring itself. Designing systems that direct C–H activation selectively toward remote C–H bonds in the pyridine ring is also quite challenging.^14^

An alternative approach to selective pyridine activation emerged recently, in particularly through the work of Nakao and co-workers (Figure 1C) and our group (Figure 1D). It utilizes rhodium and iridium complexes of pincer ligands^15,16^ that combine a central boryl or aluminyl donor with a pair of flanking phosphines. Pincer ligands with central boryl^17^ and aluminyl^18−21^ donors have come to the fore only in the recent 10–15 years, and offer unique reactivity pathways^22^ owing to the Lewis acidic “non-innocence” of the boryl/aluminyl, as well as the low electronegativity of B and Al.^23,24^ It generally relies on the capture of the basic nitrogen atom in the heterocycle not by the transition metal, but by the Lewis-acidic B or Al.

Our group’s utilization of the boryl-centered PBP pincer^25−27^ envisaged the binding of pyridine nitrogen not to the transition metal, but to the boron of the pincer (Figure 1D). This directs the transition metal (Ir) toward the *ortho*-CH bond in the pyridine ring with exquisite selectivity and without significant regard to the substitution in other positions. We observed similar selectivity with Rh.^28^ The Nakao group’s underlying hypothesis is ostensibly similar but with an aluminyl-centered pincer attached to Rh (Figure 1C), including examples of catalytic derivatization of pyridines in the *ortho*-position.^20,29,30^ Other examples of *ortho*-selective C–H activation exist, although they may require specific substitution patterns.^31−37^ The use of pyridine-binding main-group Lewis acids more remotely connected to the C–H activating transition metal for enforcing varying types of selectivity has also been explored.^38−101^ It is worth noting that Ibrance, Kisqali, and Verzenio (Figure 1A) each possess both C–H bonds *ortho* to a heterocyclic nitrogen (offering potential for further functionalization), as well as C–C or C–N bonds *ortho* to a heterocyclic nitrogen (that potentially could have been fashioned via *ortho*-CH activation and subsequent functionalization).

In this study, we set to out to explore in detail the mechanistic pathways involved in the C–H activation of pyridine by (PBP)Ir complexes in Figure 1D. In previous experimental work, we used two different precursors for this reaction: compounds **1** and **3**, with the latter showing superior reactivity. Compound **1** already has a three-coordinate, Lewis-acidic boron center, but is saturated at Ir. Compound **3** is an unsaturated, 16-electron Ir complex, but possesses a four-coordinate boron. Thus, both **1** and **3** must undergo certain steps prior to being able to add the C–H bond of pyridine and form the product **2**. On the one hand, we wished to delineate the generation of the species **4** directly responsible for the capture and C–H activation of pyridine. Our investigations revealed a complex web of mechanistic possibilities that includes a viable, unprecedented mechanism for the reductive elimination of benzene from the boryl complex **3** and its Rh analog. This mechanism involves a concerted process of C–H bond formation at boron, coupled to the concomitant formation of a full-fledged boron–metal bond. It represents a genuinely novel pathway for the formation of C–H bonds in boryl-metal systems, and potentially other element–element bonds.

On the other hand, we wished to explore the nuanced steps involved in the activation of pyridine by **4** and examine the origin of the *ortho*-selectivity. Recently, Ke and co-workers examined the reaction of pyridine with (PBP)Ir(CO)_2_ (**1**) (but not starting from **3**) using exclusively density functional theory (DFT) methods.^42^ While the computational component of the present report partly overlaps in scope with the Ke study, the questions addressed here are somewhat different and additional relevant reactions are considered. It might be argued that the work by Ke and co-workers was more focused on the delineation of the multiple conceivable pathways for the C–H activation of pyridine and ruling out several that possess implausibly high activation barriers. We benefit from this insight by Ke and co-workers, but also our own experimental data, and provide a more nuanced and expanded understanding of the mechanism that combines theoretical and experimental evidence.

## Results and Discussion

### Experimental Analysis: Kinetics of Benzene Loss from **3**

**Scheme 1:**
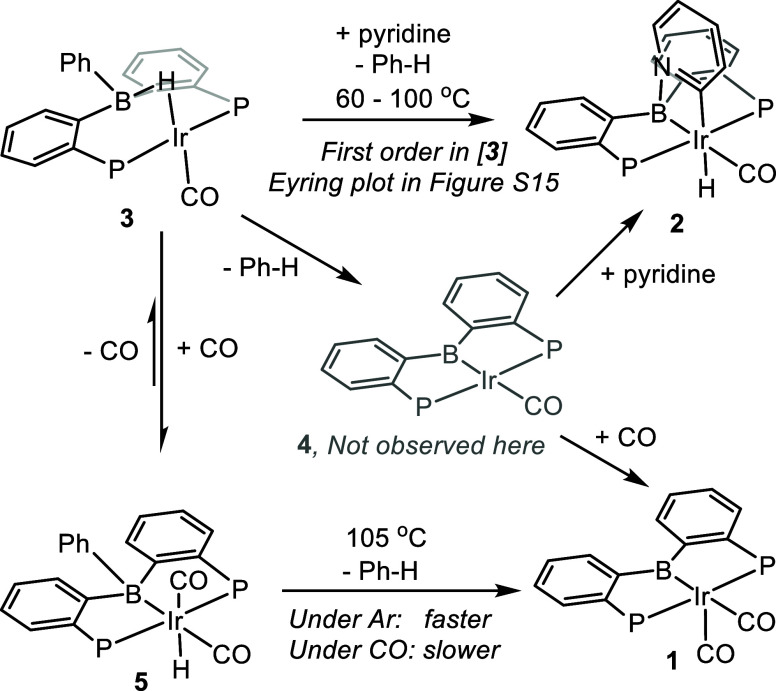
Thermolysis of Compounds **3** and **5**

First, the kinetic behavior of **3** in the benzene elimination reaction was examined. Thermolysis of 0.040 M C_6_D_6_ solutions of **3** in the presence of varying amounts of pyridine (0.40–1.60 M range) under pseudo-first-order conditions proceeded via clean first order decay in [**3**], with no significant dependence on pyridine concentration (see Scheme 1 and Supporting Information (Figure S27)). Moreover, the rate of the reaction was indistinguishable in the presence of pyridine-*d*_5_ or pyridine-*h*_5_. An Eyring analysis of this reaction in the 60–100 °C range yielded the activation parameters of Δ*H*^‡^ = 25.9(10) kcal/mol and Δ*S*^‡^ = −3(3) cal/mol·K. These data are fully consistent with the rate-limiting step of the reaction being a unimolecular process that does not involve pyridine.

We also considered whether benzene elimination can proceed from the dicarbonyl complex **5** (Scheme 1). Thermolysis of **5** at 105 °C under atmosphere of either CO or Ar resulted in an apparent first-order decay and the formation of **1** in both cases. However, the reaction under CO atmosphere was five times slower, which suggests that dissociation of CO is kinetically significant and thus the overall benzene loss starting from **5** proceeds entirely or at least predominantly via **3**.

### DFT Analysis: Benzene Elimination from **3**

**Figure 2:**
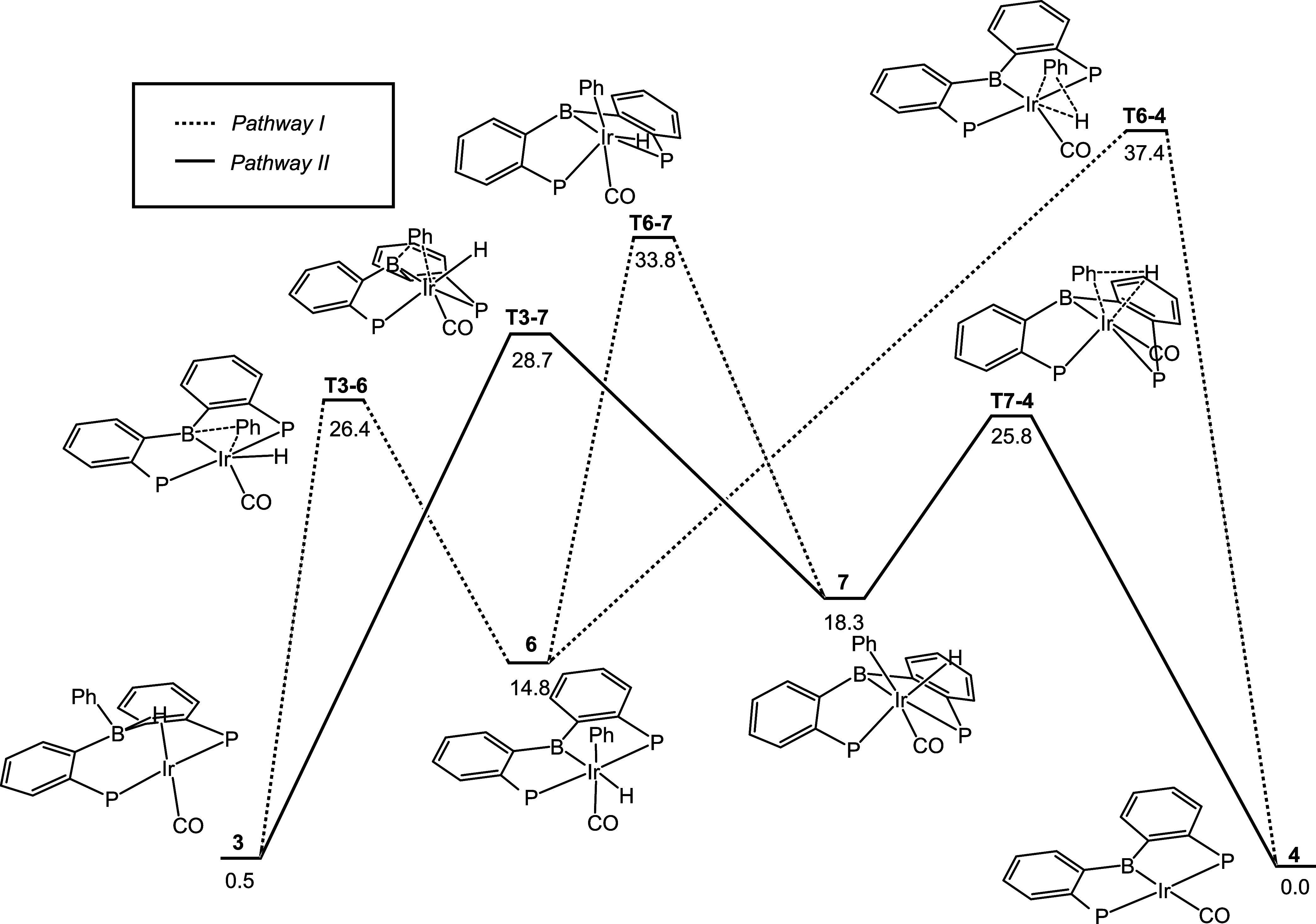
DFT-calculated pathways for the formation of active species starting from **1** or **3** and the binding of pyridine. Isopropyl groups on phosphorus atoms are omitted for clarity here and in the following graphics. In this and all following Figures, Gibbs energies are given under the structure number (in kcal/mol), calculated at the SMD(Bz)-B2PLYPD3/SDD/6-311+G(d,p)//B97D3/LANL2DZ/6-31G(d) level of theory.

For the elimination of Ph–H from **3**, we first analyzed two pathways that reflect the more traditional notion of C–H bond formation via concerted RE from Ir (Figure 2). Thus, the first step from **3** in Pathways I and II is a migration of the Ph group from B to Ir. It happens in concert with the formation of the terminal Ir–H and Ir–B bonds out of the bridging Ir–H–B unit in **1** via **T3–6** (Pathway I) or **T3–7** (Pathway II). The transition states **T3–6** and **T3–7** are “late” in the sense that the Ir–H/Ir-B/Ir–C distances approach the final individual bond distances in the distorted octahedral intermediates **6**/**7** while the B–H and B–C distances are already very far from the normal bond lengths. The conversion of **1**–**6** or **7** positions the new boryl ligand in the same coordination site previously occupied by the bridging H in **1**. The difference between Pathways I and II is in the mode of the distortion of the original P/P/Ir/CO fragment in **1** to accommodate the addition of Ph and H. In Pathway I, the carbonyl ligand bends away from its original position, whereas in Pathway II, it is one of the phosphines.

The first transition state **T3–6** in Pathway I is lower by 2.3 kcal/mol than the corresponding **T3–7** in Pathway II and the corresponding octahedral Ir product **6** is lower in energy by 3.5 kcal/mol than **7**. However, there is a large difference in the barriers for the subsequent RE to produce **4**: in Pathway I, **T6–4** lies 11.6 kcal/mol above **T7–4** in Pathway II. A transition state (**T6–7**) was also found for the isomerization of **6** into **7**, and it lies 5.1 kcal/mol higher than **T3–7**. This implies that while **6** may be formed competitively with **7** in this reaction, the lowest energy pathway from **6** to **4** is to revert to **3** and proceed via **7** through Pathway II, rather than via direct isomerization of **6** into **7**, or via RE from **6**. Thus, on the whole, Pathway II is a lower-barrier pathway to **4** than Pathway I. The substantial distortion of the PBP ligand in **7** may be responsible for its relatively high energy, but it may also “spring-load” **7** such that accessing **T7–4** is assisted by the return of the PBP ligand into its more natural meridional geometry. The experimental activation parameters (Δ*G*_298_^‡^ = 26.8(13) kcal/mol, vide supra) agree well with the DFT-calculated values for Pathway II (Δ*G*_298_^‡^ = 28.2 kcal/mol).

### DFT and Experimental Analysis of TMARE

**Figure 3:**
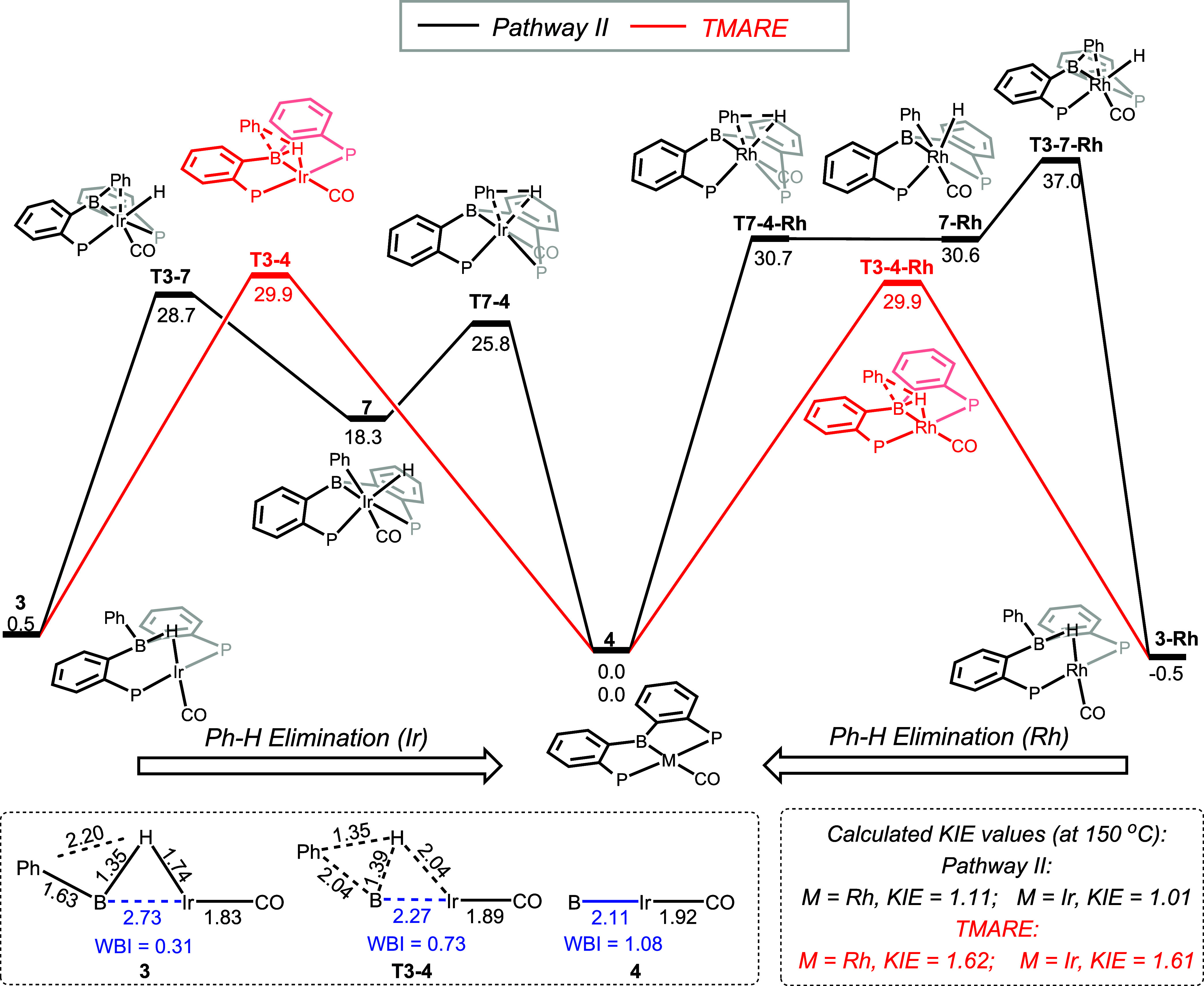
Top: Comparison of the calculated energy profiles for Pathway II and for TMARE for Rh and Ir, with Gibbs energies in kcal/mol. Bottom left: Selected calculated bond distances and Wiberg bond indices for the Ir–B bonds in **3**, **T3–4**, and **4**. Bottom right: Calculated KIE values for Pathway II and for TMARE.

In considering the possible mechanisms for Ph–H loss from **3**, we also examined C–H bond formation directly “from boron”, without involving an Ir–Ph interaction. To our surprise, the transition state for such an elimination (**T3–4**, Figure 3) was found to lie at only 29.4 kcal/mol relative to **3**. This is only 1.2 kcal/mol higher than **T3–7** and thus this new pathway cannot be ruled out as a competitor to Pathway II. Hypothetical elimination of Ph–H from a free hydridotriarylborate would leave behind a very high-energy free diarylboryl anion^43^ and thus very unlikely to occur. In the case of **3**, however, the incipient boryl anion is captured by the Ir fragment. This process can be analyzed as a transition metal-assisted reductive elimination of Ph–H from a main-group element (boron), or TMARE.

Given the exciting novelty of TMARE, we explored tweaking the original system so that TMARE becomes unambiguously preferable to Pathway II. To our delight, DFT calculations predicted that a simple switch from Ir to its lighter congener Rh would invert the preference in favor of TMARE. Loss of benzene from **3-Rh** via Pathway II was calculated to have a much higher barrier of 37.5 kcal/mol for Rh than for Ir, whereas the TMARE barrier for Rh at 30.4 kcal/mol is roughly the same as for Ir (Figure 3).^44^

The divergent effect of the Rh/Ir choice on the activation barriers for Pathway II vs TMARE can be understood by considering that **T3–7** leads from a 16-electron, monovalent Rh/Ir complex **3** to an 18-electron, trivalent Rh/Ir complex **7**. Although it also involves the conversion of a bridging hydride to a terminal hydride, **T3–7** is a transition state for the insertion of Rh/Ir into the B-Ph bond (formally an oxidative addition). The 5d metal Ir should favor this insertion and the formation of an 18-electron structure to a greater degree than the 4d metal Rh. On the other hand, in TMARE, the influence of the nature of the transition metal on **T3–4** is less direct. It could be surmised that Ir might be more favorable than Rh for the incipient boryl-metal bond formation in **4**, but it likely also has a stronger interaction with a bridging hydride in **3** that it has to “give up”.

The finely tuned and concerted nature of the Ph–H elimination via TMARE can be illustrated by considering the changes to the M-B distance and bonding from the starting compound **3** to **T3–4**, to the product **4** (Figure 3). Compound **4** possesses a normal, 2-center-2-electron single metal–boron bond. The Ir–B distance is comparable to other unambiguous metal-boryl bond lengths, and the Wiberg bond index (WBI) is 1.08. Compound **3** instead features a 3-center-2-electron system of a hydride bridging B and Ir, with a very long B–Ir distance and a WBI value of only 0.31. The transition state **T3–4** connecting these two structures already gains much of the increase in the B–Ir bonding as indicated by the shortening of the B–Ir distance and the increase of WBI to 0.73. The situation with the Rh analogs is essentially the same: the requisite distances for the Rh analogs are within <0.02 Å of the ones shown in Figure 3. Indeed, this transformation should be characterized as a reductive elimination from boron that is aided by the transition metal. As remarked above, the developing electron-density at boron as a result of the Ph–H RE progression is captured by the Rh/Ir center (eventually as a fully formed boryl-Rh/Ir bond in **4**). Put in other words, this is a concerted reductive elimination where significant changes to bonding take place among four elements (B, C, H, and Rh/Ir) instead of the usual three.

We decided to explore the H/D kinetic isotope effect (KIE) as a potential experimental confirmation of the preference for TMARE. The computed KIE values for the elimination of Ph–H vs Ph–D via the two competing pathways were quite similar for Rh and Ir (Figure 3). Unfortunately, we were not able to prepare **3** labeled selectively with D in the hydride position because of extensive H/D exchange with the isopropyl groups. However, it did prove possible for Rh.

**Scheme 2:**
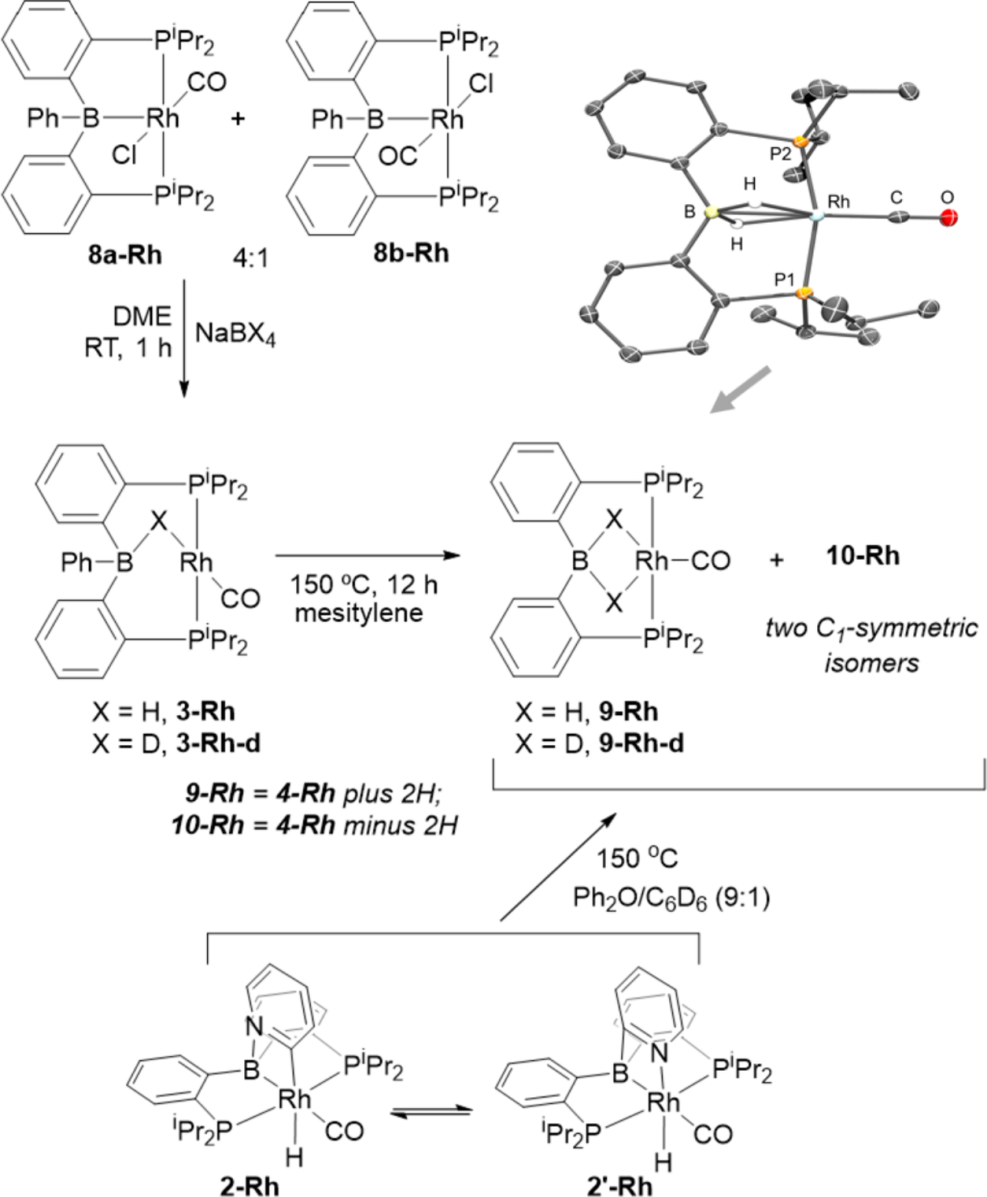
Synthesis of **3-Rh** and **3-Rh-d** and Their Thermolysis

Treatment of **8a/b-Rh** (previously reported by Bourissou)^45^ with NaBH_4_ or NaBD_4_ allowed the preparation of the two isotopomers of **3-Rh** (Scheme 2). Their thermolysis proceeded slower than the thermolysis of **3**. We originally performed the thermolysis in the presence of DMAP, anticipating activation of the latter, as in the case of Ir. However, we discovered that the outcome of thermolysis of **3-Rh** was not affected by the presence of DMAP. Thermolysis led to the quantitative release of Ph–H or Ph–D. However, instead of the expected **4-Rh**, it resulted in the formation of a mixture of Rh complexes, approximately half of which constituted **9-Rh**, and the other half consisted of two dissymmetric compounds (**10-Rh**) in a ca. 4:1 ratio. The identity of **9-Rh** was confirmed by the preponderance of NMR data, and by an X-ray diffractometry study in the solid state. Although the hydrides could not be reliably placed, the structure of **9-Rh** bears a striking similarity to the previously reported structure of (PBP)IrH_4_ which also contained two bridging hydrides.^26^ In particular, they both display the unusual coplanarity of the aromatic rings we have not observed in any other PBP complexes. In addition, the long Rh–B distance of 2.273(3) Å in **9-Rh** is too long for a simple Rh-boryl bond, but is consistent with the borohydride binding to Rh. **9-Rh** was also the sole product when the thermolysis of **3-Rh** was conducted under H_2_ atmosphere. DFT calculations indicated that addition of H_2_ to **4-Rh** to form **9-Rh** is favorable by 12.8 kcal/mol in Gibbs energy. The lack of pyridine activation was traced to thermodynamic origins by performing thermolysis of **2-Rh**(28) and observing the formation of a similar mixture containing **9-Rh** and the dissymmetric isomers **10-Rh** (Scheme 2).

We struggled to establish the identity of the dissymmetric compounds **10-Rh**. In the ^31^P{^1^H} NMR spectra, they each display a pair of doublets of doublets from the coupling of the two inequivalent ^31^P nuclei to each other (large and very similar *J*_PP_ = 275 and 276 Hz, indicating *trans*-disposition of the phosphines), and to ^103^Rh. One of the ^31^P NMR signals in each of these compounds is significantly shifted upfield, by 60–80 ppm, from the other; this is suggestive of cyclometalation of the isopropyl group taking place. We hypothesize that these are two isomers arising from some sort of dehydrogenation involving diastereotopic CH_3_ groups in P*^i^*Pr_2_. Unfortunately, we were not able to isolate them as separate pure materials and the low symmetry gave rise to NMR spectra that were difficult to decipher with confidence.

**Scheme 3:**
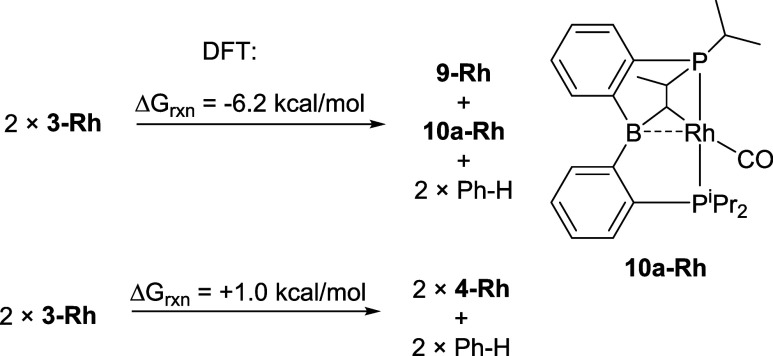
DFT Calculated Energies for the Conversion of **3-Rh** (with Loss of Benzene) into (a) **9-Rh** and the Putative **10-Rh** and (b) to **4-Rh**

DFT studies identified one thermodynamically plausible isomer of **10-Rh** (Scheme 3: **10a-Rh**). The conversion of **3-Rh** into **9-Rh** and **10a-Rh** (and benzene) was calculated to be favorable. The structure of **10a-Rh** (a B/Rh bridging alkylidene) was influenced by the earlier observation of bridging alkylidenes in the reactions of **1** with olefins.^22^ We also calculated the relative energies of several other conceivable isomers that were considerably higher in energy (see Figure S3). ^13^C{^1^H} NMR spectra of mixtures containing **10-Rh** gave rise to resonances in the 60–75 ppm, range, which is similar to where the bridging alkylidene carbons arising from the reactions of **1** with olefins were found,^22^ but we cannot be confident in the assignment of the dissymmetric products as **10a-Rh**. Nonetheless, the fact that DFT suggests that there is at least one isomer of **10-Rh** corresponding to a favorable reaction is encouraging. The exact identity of **10-Rh** is not important in the analysis of the mechanism of Ph–H loss from **3-Rh**, since we assume **10-Rh** forms after the rate-limiting loss of Ph–H.

Kinetic studies of the thermolysis of **3-Rh** at 150 °C displayed well-behaved first-order behavior. The measured *t*_1/2_ ≈ 1.3 h corresponds to a Gibbs energy barrier of ca. 32.5 kcal/mol,^46^ which is much closer to the Gibbs energy barrier calculated by DFT for TMARE (30.4 kcal/mol via **T3–4-Rh**) than for Pathway II (37.5 kcal/mol). We then proceeded to measure the rates of the decay of **3-Rh** and of **3-Rh-d** under matching conditions. We found that at 150 °C, H/D exchange between the hydride/deuteride in **3-Rh** and the solvent (cyclooctane or mesitylene) took place and interfered with the KIE determination. Because of this, it was necessary to thermolyze **3-Rh** in a protio-solvent, and **3-Rh-d** in a deuterated solvent. With that, the D label in **3-Rh-d** was transferred near-quantitatively to the Ph–D product. No H/D exchange with the –P*^i^*Pr_2_ groups was detected. The corresponding KIE value was determined to be 1.62(15), in excellent agreement with the DFT prediction for TMARE. We believe that this experimental result provides strong support for the hypothesis of Ph–H loss from **3-Rh** via a genuinely novel mechanism.

It is interesting to ponder why the two pathways possess different KIEs, and also why the KIE values are not very large for either. There is a substantial change in the fate of H in **T3–7** relative to **3**: in **3**, the hydride is bridging between B and Rh/Ir, but in **T3–7**, it is essentially already a terminal hydride on Rh/Ir. The H–Rh/Ir distance in **T3–7** is actually slightly shorter than in **7**. In other words, by the time the reaction coordinate reaches **T3–7**, the hydride transfer to Rh/Ir is completely done and the difference between the hydride positions in **3** and **T3–7** is essentially a difference between two ground-state geometries, with little H/D variance.

On the other hand, the hydride in **T3–4** is not in a ground-state geometry. It is truly in the middle of the transfer from the bridging position in **3** to the fully formed C–H bond. In **T3–4**, it is interacting with C, B, and Rh/Ir in a nonclassical fashion. We therefore offer that it makes sense that the KIE value for Pathway II is close to unity, but that for TMARE is modest, but larger.

### DFT Analysis (Back to Ir): C–H Activation in **4** and Subsequent Isomerization

**Figure 4:**
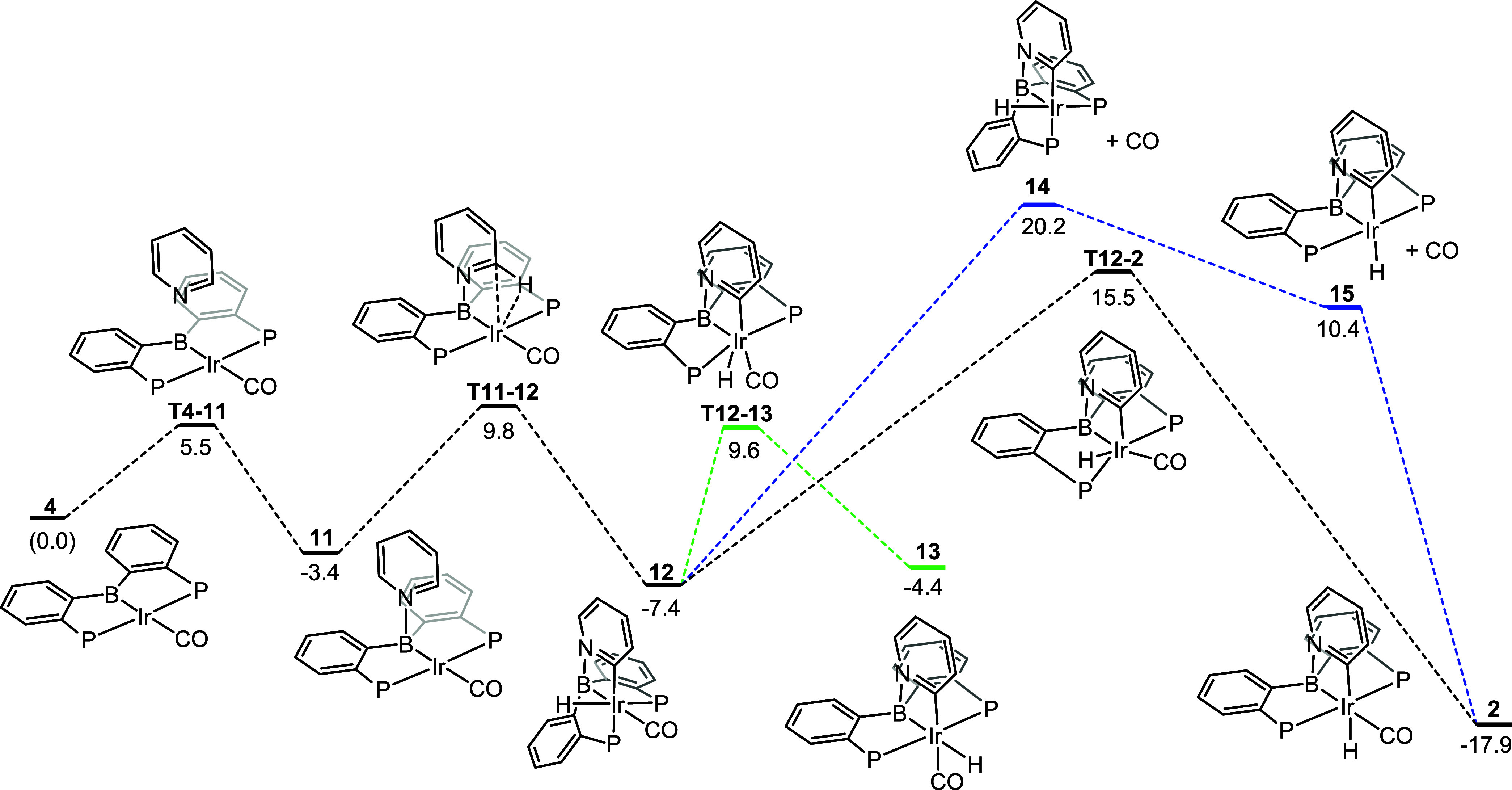
DFT-calculated pathways for the activation of pyridine by **4** and isomerization to **2**.

At the next stage, we set out to evaluate the coordination of pyridine to **4** (to give **11**, Figure 4), the insertion of Ir into the C–H bond in **11**, and the subsequent isomerization to the final product **2** (Figure 4). In our 2017 paper,^27^ we hypothesized that the kinetic product of the initial insertion would be **13** (H *trans* to B). However, in the present DFT calculations, we also considered the formation of the less symmetric isomer **12** (H *trans* to P). The relationship between these two isomers is reminiscent of the relationship between **6** and **7** (Ph migration): for **13**, the CO ligand is moved out of the coordination square plane in **11**, while for **12**, it is one of the phosphines that is moved out to accommodate the addition of the C–H bond to Ir. **12** is lower in energy than **13** by 3.0 kcal/mol and is accessible from **11** via **T11–12** with a barrier of only 13.2 kcal/mol. The formation of **12** from **11** is 4.0 kcal/mol downhill in Gibbs energy.

The intermediate **12** is the kinetic product of C–H oxidative addition to Ir with C(pyridyl) and H *cis* to each other. Its conversion to the final product **2** necessitates an isomerization step. Intramolecular isomerization of a six-coordinate d^6^ metal center would appear surprising; nonetheless, an intramolecular pathway for this isomerization was found with the transition state **T12–2** lying 22.9 kcal/mol above intermediate **12**. The conversion of **12** into **2** requires the H and the phosphine *trans* to C(pyridyl) to switch places and the structure of **T12–2** reflects their intermediate positions along this path. There is only a 0.003 Å difference in the Ir–H distance in **12** vs **T12–2**, which suggests that the hydride migrates through the coordination sphere while maintaining a covalent bond with Ir.

**12** and **13** are connected by an isomerization transition state **T12–13** that lies 5.9 kcal/mol below the **T12–2** and 0.2 kcal/mol below **T11–12**. We attempted to find a transition state connecting **13** and **2** but those attempts converged only on **T12–13**. Thus, even if **13** is formed, it likely can only proceed to **2** via the intermediacy of **12**.

CO dissociation from **12** could be envisaged as an initial step of alternative pathway to **2**. However, the energy of **14**, the product of CO loss from **12**, was already found to be 4.7 kcal/mol higher than **T12–2** and we did not pursue this mechanism further. Interestingly, the loss of CO from **12** (to give **14**) and the loss of CO from **2** (to give **15**) are very similarly endoergic: 27.6 and 28.3 kcal/mol, respectively.

We also considered a pathway to isomerize **12** into **2** that proceeds via the migration of C_pyridyl_ from Ir to B, along the lines of our more general recent study of the preferences of the C,N-bridging pyridyls in the related systems.^28^ However, a few of the necessary transition states along this pathway were found to lie 5–8 kcal/mol above **T12–2** (see Figure S1).

We previously noted that the facility of the concerted oxidative addition of a C–H bond here is surprising as (pincer)IrCO complexes containing other pincers do not undergo such reactions easily.^47,48^ Three factors can be brought up by way of rationalization. First, the intramolecular nature of the C–H activation step in **11** makes it easier. Second, it is possible that the electron-releasing properties of the boron ligand here perturb the electronic nature of the Ir center significantly vs the analogous (PNP)IrCO or (PCP)IrCO complexes. Third, the geometry of **11** is distorted from the idealized square planar, as the tetrahedral geometry about boron necessarily causes some puckering of the (PBP)Ir framework. This distortion is en route to the geometry of **T11–12** and **12** in which the PBP ligand becomes facial, i.e., one of the phosphines moves further “below” the original square plane.

It should be noted that all the transition states after **T3–7** lie well below it, and none of the local barriers following **T3–8** are larger than the difference between **3** and **T3–7**. This means that in the thermolysis of **3** with pyridine, only the starting materials and the products should be observed, as is the case experimentally.

### DFT Analysis: Comparison with the Ke Study

The Ke study^42^ considered several mechanistic possibilities for the addition of the C–H bond of pyridine to Ir in **4**. In particular, they found that the direct addition of the C–H bond to Ir in **4** (without any interaction of boron with the pyridine fragment) corresponds to an unreasonably high activation barrier (>35 kcal/mol). With that insight in hand, we did not pursue analogous calculations. They have also considered a mechanism for the addition of the pyridine C–H bond to B–Ir that is essentially a reverse of TMARE, but they did so for the reaction of **1** not **4**, and found a relatively high barrier. Similarly to our work, Ke and co-workers concluded that intramolecular insertion of Ir into the C–H bond in **11** is the kinetically preferred path. However, the calculations in the Ke study ascribe a much higher relative energy to the dissymmetric intermediate **12**. In their calculations, **12** is 7.4 kcal/mol higher in Gibbs energy relative to **11**, while we calculated it to be 4.0 kcal/mol lower in energy. The anomalously high Gibbs energy for **12** also leads to a much higher energy calculated by Ke for the analog of **T12–2**.

**Figure 5:**
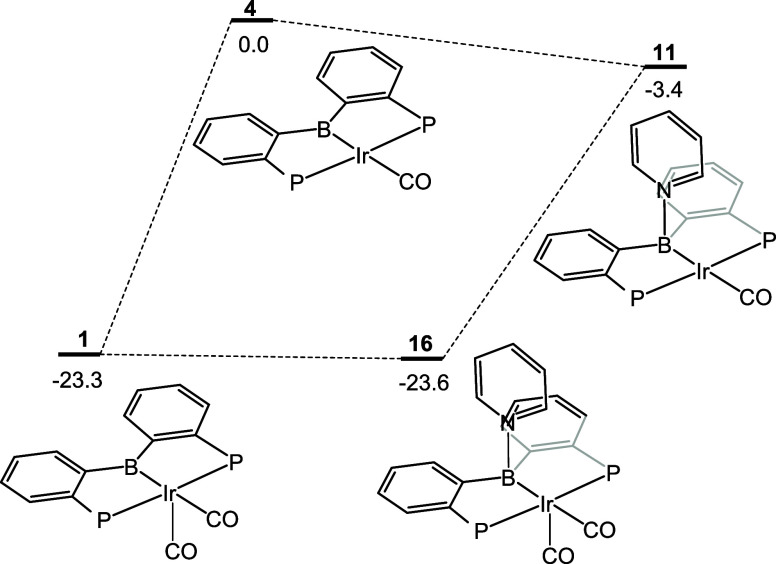
DFT-calculated energies for the loss of CO and binding of pyridine starting from **1**.

Other notable disagreements with the Ke work are found in the ligand exchanges associated with the dicarbonyl complexes. In their work, the binding of pyridine to **1** was calculated to be endergonic by 6.9 kcal/mol. However, our calculations suggest a slightly (by 0.3 kcal/mol) exoergic binding (Figure 5). Experimentally, we determined the thermodynamic parameters for binding pyridine to **1** to be Δ*H* = −12.9(6) kcal/mol and Δ*S* = −42(2) cal/mol·K (via a variable-temperature ^31^P NMR study of a C_6_D_6_ solution of **1** in the presence of pyridine, see Figure S11). This corresponds to Δ*G*_298_ = −0.4(8) kcal/mol and agrees very well with our computational prediction. In addition, the Ke study predicts that the loss of CO from **1** is endergonic by only 5.1 kcal/mol (vs 23.3 kcal/mol in our calculations). The 5.1 kcal/mol value seems too low (barring an unusual large *kinetic* barrier for CO loss), given the experimental stability of **1** to vacuum. As our findings conform very nicely to the experimental observations in this and other regards (vide infra), we did not investigate the origin of the above discrepancies with the Ke work.

### Experimental Analysis: Observation of **4** and **12**

**Figure 6:**
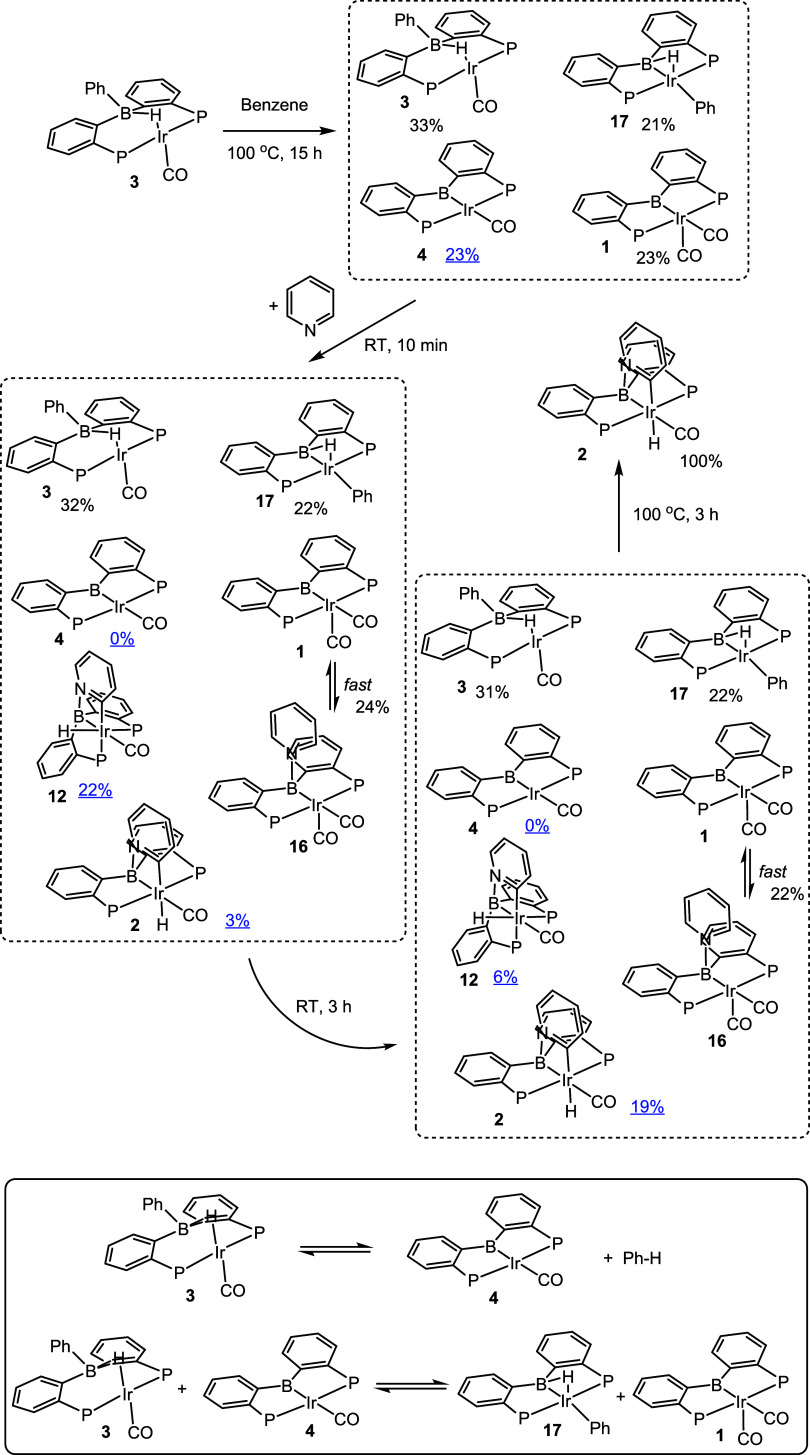
Observation of (PBP)IrCO (**4**) upon thermolysis of **3** and its subsequent reaction with pyridine, and (bottom, in the solid-lined box) the equilibria describing the mixture resulting upon thermolysis of **3** in benzene.

Thermolysis of **3** in C_6_D_6_ in the absence of pyridine produced a rather unexpected result (Figure 6): a mixture of **1**, **3**, **4**, and **17** was formed, apparently in equilibrium with each other on the time scale of >10 h at 100 °C. Remarkably, this is a completely different stoichiometric outcome than the fate of **3-Rh** upon thermolysis (Scheme 2). Compounds **1**, **3**, and **17** were previously characterized independently.^26,27^ Compound **4** was tentatively identified on the basis of an ^11^B{^1^H} NMR signal at 106.0 ppm (indicative of trigonal planar boron), a ^31^P{^1^H} NMR signal at 81.7 ppm, and selected ^1^H NMR resonances displaying C_2v_ symmetry (see Figure S13 in the SI). The composition of the resultant mixture can be understood via a combination of two equilibria: (a) reversible loss of benzene from **3** to make **4** and (b) a reversible transfer of CO from **3** to **4** to make **1** and **17** (Figure 6). Although one could choose another pair of equilibria to define the system, it is not possible to describe the situation with a single equilibrium equation. Addition of extra **1** to this equilibrium mixture, followed by additional thermolysis, led to the changes in the observed ratios of compounds according to the pair of equilibrium constant values (see Figure S49, Table S10 in the SI). DFT calculations are in excellent agreement with the nearly isoergic experimental observations: the loss of benzene from **3** was calculated to be downhill by 0.5 kcal/mol in Gibbs energy (Figure 2), while the conversion of **3** and **4** into **1** and **17** is uphill by 0.5 kcal/mol.

Although we could not isolate **4**, its presence in a mixture that undergoes exchange only slowly at RT offered an opportunity to study its reactivity. Treatment of the mixture containing 23% **4** with one equiv (per total Ir) of pyridine at ambient temperature resulted in an immediate color change, the disappearance of the NMR resonances of **4**, and the appearance of the corresponding amount of a compound we tentatively assign as the dissymmetric intermediate **12**. Compound **1** in this mixture binds pyridine in a fast, reversible equilibrium, giving rise to a single weight-averaged resonance of **1/16** by NMR spectroscopy; this equilibrium was separately examined with pure **1** (vide supra). The other components of the mixture (**3** and **17**) were not affected by the addition of pyridine. DFT calculations indicated a structure for intermediate **12** in which two inequivalent phosphines are *cis* to each other with a hydride *trans* to one of them. Consistent with this prediction, in the ^31^P{^1^H} NMR spectrum, an AB system with a modest ^2^*J*_P–P_ = 18 Hz was observed (as expected for a *cis*-PP-configuration). In the ^1^H NMR spectrum, a hydride resonance displaying disparate coupling to two ^31^P nuclei (^2^*J*_H–P_ = 29 (*cis*) and 108 (*trans*) Hz) was recorded at δ – 10.29 ppm. Over several hours, the resonances belonging to intermediate **12** decayed, with the concomitant rise of the resonances of the final product **2**. Thermolysis of this mixture for 3 h at 100 °C led to the complete conversion of all the compounds into **2**.

These observations are gratifyingly consistent with the DFT reaction barrier predictions. The addition of pyridine to **4** to form **11** is favorable and rapid, and the subsequent barrier (**T11–12**) for the insertion of Ir into the C–H bond is only 13.2 kcal/mol. This indeed suggests that the pyridine adduct **11** should not be observed at ambient temperature as it converts to **12** too rapidly. The isomerization of **12** into **2** was calculated to proceed with a local barrier of 22.9 kcal/mol, a good match to the apparent experimental ca. 1.5 h half-life (corresponds to ca. 22.5 kcal/mol in Gibbs energy barrier).

### Experimental Analysis: Competition between Different Pyridines

**Chart 1:**
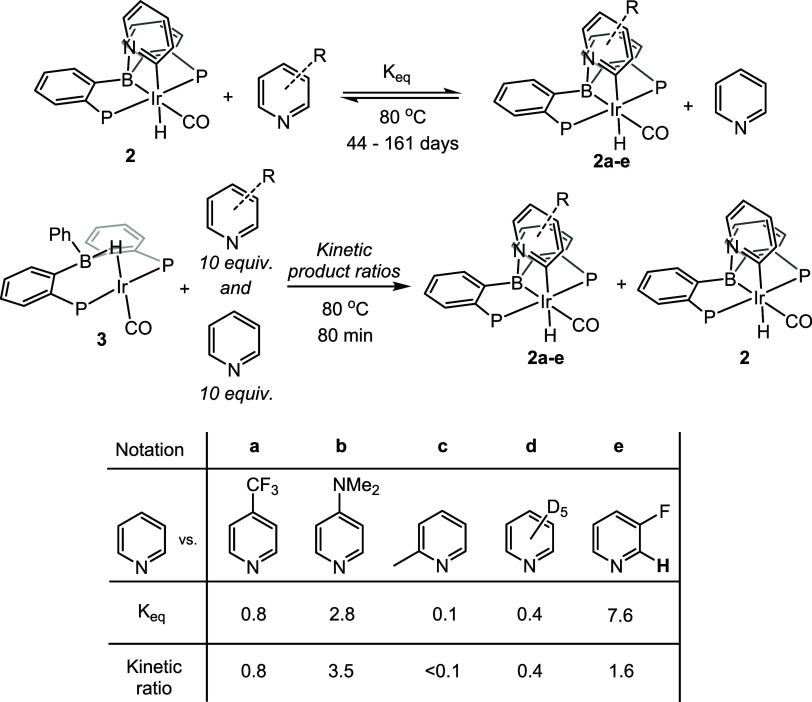
Determination of Equilibrium Constants and Kinetic Selectivity in the Activation of Various Pyridinesa

The formation of the pyridine activation product **2** is reversible: thermolysis of **2** in the presence of another pyridine generates a new product **2a**–**e** (Chart 1). These reactions proceed without decomposition, but are slow. At 80 °C, the establishment of the equilibrium takes months, but we were able to achieve them for several pairings (Chart 1).

a Top: conditions of the *K*_eq_ determination. Middle: conditions for the determination of kinetic product ratios. Bottom: summary of results. *K*_eq_ and kinetic ratio values greater than 1 indicate preference over the parent pyridine (C_5_H_5_N).

The relative thermodynamic preferences in these equilibria can be considered to arise from a combination of the relative strength of the N → B dative bond and the relative strength of the covalent Ir–C bond. The former would be buttressed by the greater basicity of the pyridine/pyridyl (such as in DMAP), but the latter likely benefits from the presence of an electron-withdrawing group (such as in 4-F_3_CC_5_H_4_N).^49^ These effects ostensibly counteract each other in a number of cases, resulting in equilibrium constants not far from unity for compounds **2a**–**e**. In considering the two possible isomers (2- vs 6- positions on the ring) for the addition of 3-fluoropyridine (to give **2e**), the strength of the N → B bond should not differ much, thus the strong preference for the only observed isomer **2e** must derive from the stronger Ir–C bond. Indeed, the strengthening of a metal-C(aryl) bond by an *ortho*-fluorine is well understood.^50^

In order to probe the kinetic preference for a particular pyridine after the rate-limiting benzene elimination, **3** was thermolyzed at 80 °C for 80 min in the presence of different pairings of the parent pyridine with other pyridines. The partial conversion at this time point and the presence of an equal 10-fold excess of each pyridine allowed us to neglect the changes in their concentration during the time course. Because the time scale of this experiment is 3–4 orders of magnitude lesser than the time needed for establishing the equilibrium among **2/2a-e**, the ratios reported in Chart 1 can be viewed as ratios of the rates of formation of the corresponding products. In the reaction with 3-fluoropyridine, again, only one isomer of **2e** corresponding to the activation of the 2-CH position was observed at any point of the reaction.

The ca. 0.4 kinetic ratio of **2d** vs **2** (KIE = *k*_H_/*k*_D_ = ca. 2.5) suggested that the kinetic product-selecting process is not merely the coordination of a particular pyridine to boron, but also involves the C–H activation step. The modest ratios in entries for **2a**–**e** are also consistent with the notion that the formation of the N → B adduct **11** alone is not guiding the kinetic product selection, as in that case it would have been expected to see much more pronounced differences owing to the very different basicity of F_3_C/H/Me_2_N-substituted pyridines.^51^

DFT offers a convincing explanation of the observed trends. Pyridine coordination to **4** leads to **11**, which is followed by low-barrier insertion of Ir into the C–H bond (via **T11–12**) to give **12**. The barrier to proceed from **12** to the final product **2** is higher than the barrier for pyridine loss from **11**. This suggests that a pre-equilibrium is established for the dissymmetric intermediates **12** in the presence of two different pyridines. The “kinetic” ratios we observe are then products of the equilibrium ratios among the various analogs of **12** and the ratios of rates of isomerization of the various analogs of **12** into the final products **2/2a-e**. From this perspective, and especially if the (undetermined) rates of **12a**–**e** → **2a**–**e** isomerization do not differ significantly among the various pyridines, it is not surprising that the “equilibrium” ratios are quite similar (but not identical) to the respective “kinetic” ratios (Chart 1). Consistent with this, the DFT-calculated equilibrium ratio of **2** and **2d** is 2.8, close to the observed KIE value of 2.5.

### Experimental Analysis: Kinetic Studies of the Reverse Reaction

**Scheme 4:**
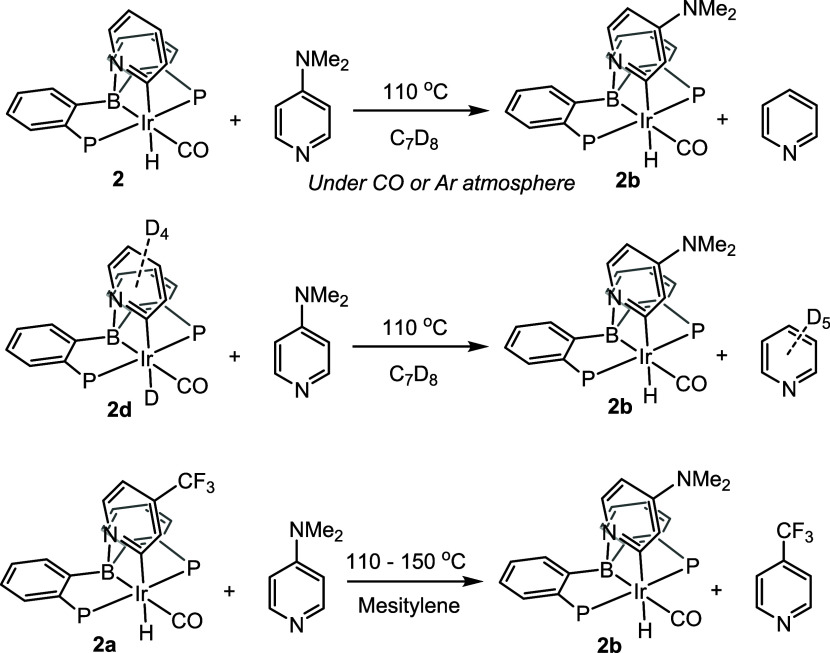
Experiments for the Mechanistic Analysis of Loss of Pyridines from Compounds **2**, **2a**, **2d**

The reaction of the product **2** with another pyridine presented an attractive opportunity to take advantage of the principle of microscopic reversibility and probe the mechanistic picture in the reverse direction. Thermolysis of **2** at 110 °C in the presence of 10-fold excess DMAP^52^ (Scheme 4) was followed by NMR spectroscopy in two experiments: one under argon atmosphere, another under atmosphere of CO (Scheme 4). In both cases, the reaction followed a first-order profile and the rate showed no dependence on the presence or absence of CO. An analogous experiment was set up for the thermolysis of **2d** (under Ar) with DMAP. The rates for the protio vs deuterio reactions were 2.0(2) and 1.7(1) × 10^–5^ s^–1^, for the KIE of 1.2(1). Given the slow rates and the high temperatures needed for the Eyring analysis, we opted for the thermolysis of **2a** with DMAP. **2a** is slightly less favorable (vide supra) relative to **2** and was anticipated to give a slightly faster reaction, as well as an additional convenient ^19^F NMR spectroscopic handle. Determination of the rate constants of the decay of **2a** in the 110–150 °C range yielded Δ*H*^‡^ = 29.2(12) kcal/mol and Δ*S*^‡^ = −5(3) cal/mol·K (for a Δ*G*_298_^‡^ = 30.7(15) kcal/mol). We conclude that the rate-limiting step for the loss of a pyridine from **2** is unimolecular, does not require CO dissociation, and does not involve significant Ir–H bond cleavage. This is qualitatively in accord with the computational findings. Quantitatively, the ejection of pyridine from **2** was calculated (via **T12–2**) to possess a Gibbs energy barrier of 33.4 kcal/mol and a calculated KIE = 1.03. Permitting that the barrier starting from **2a** should be slightly smaller, the agreement with the experiment is reasonable.

## Conclusions

In summary, the combined application of DFT theoretical methods and of experimental rate studies has allowed us to sketch out a detailed picture of the reactivity of (PBP)Ir complexes with pyridines. The active, pyridine-coordinating and -activating species was determined to be (PBP)Ir(CO) (**4**). It can be generated in situ by either the unfavorable CO dissociation from (PBP)Ir(CO)_2_ (**1**) or the approximately ergoneutral loss of benzene from (PB^Ph^P)IrHCO (**3**). Analysis of the mechanistic options for this loss of benzene led to the discovery of an unprecedented mechanism whereby the Ph–H bond formation happens “on boron”, without contact between Ph and the transition metal. The transition metal instead is involved in essentially accepting greater electron density that is left behind by the reductive elimination of Ph–H from boron, which can be described as transition metal-assisted reductive elimination (TMARE). TMARE is a process in which formation of both the C–H bond of benzene and a strong B–M bond takes place in a concerted fashion. Theoretical analysis concluded that for Ir, TMARE possesses a barrier slightly higher than the more conventional Pathway II that leads to the reductive elimination of Ph–H from the transition metal. However, for Rh, theory predicted the preference for TMARE, which was corroborated by the experimental determination of the predicted kinetic isotope effect. TMARE represents an unusual and novel pathway for the formation of C–H bonds in boryl-metal systems. It is also tempting to consider that it may have broader implications for the formation of other element–element bonds, for oxidative addition of element–element bonds (by the principle of microscopic reversibility), and for related reactions involving main group–transition metal pairs other than B–Rh or B–Ir.

(PBP)Ir(CO) (**4**) is observable, but under the conditions of its generation from **3**, it accesses complicated equilibria related to CO exchange and benzene solvent activation, and could not be prepared in pure form. Nonetheless, experimental and computational investigations are consistent with the notion of pyridine binding to the empty orbital at boron in (PBP)Ir(CO) (**4**) to be the necessary directing step that determines the selectivity of C–H activation.

The insertion of Ir into the *ortho*-CH bond of a pyridine coordinated to boron curiously produces a dissymmetric kinetic product (compound **12**) in which the two phosphine arms are approximately *cis* to each other and the C and H of the former pyridine are, as well. The formation of this dissymmetric product is rapid at ambient temperature and was observed experimentally. Its isomerization into the final product **2**, in which the two phosphines are *trans*, and the C and H are *trans*, proceeds via a concerted intramolecular reorganization of the six-coordinate Ir complex that does not require any ligand dissociation or dramatic weakening of the Ir–H or other Ir-ligand bonds.

On the whole, this study indicates that the principle of capture of the directing nitrogenous donor by a boron in a pincer ligand is indeed operative in this system. The study also suggests that increased flexibility of the pincer framework may be advantageous for more facile oxidative addition and reductive elimination reactions.

## Supporting Information Available

Details of experimental procedures, NMR spectroscopic characterization, X-ray crystallography experiment, and DFT calculations (PDF)
Coordinate files for the DFT-optimized structures (ZIP)
