# Supplementary material for: C–H Activation of Pyridines by Boryl Pincer Complexes: Elucidation of Boryl-Directed C–H Oxidative Addition to Ir and Discovery of Transition Metal-Assisted Reductive Elimination from Boron at Rh
Source: J Am Chem Soc. 2024 Oct 30;146(45):31281–94. doi: 10.1021/jacs.4c12143 (PMC11565645; doi:10.1021/jacs.4c12143)
Supplement: Supplementary file 1 — ja4c12143_si_001.pdf [file ja4c12143_si_001.pdf]

Supporting Information

C-H Activation of Pyridines by Boryl Pincer Complexes: Elucidation of Boryl-Directed C-H Oxidative Addition to Ir, and Discovery of Transition Metal-Assisted Reductive Elimination from Boron at Rh

Vinh T. Nguyen[a], R. Noah Sladek[a], Yihan Cao[a], Nattamai Bhuvanesh[a], Jia Zhou[b], and Oleg V. Ozerov[a]**

[a] Department of Chemistry, Texas A&M University, College Station, TX 77842, USA.
[b] State Key Laboratory of Urban Water Resource and Environment, School of Science, Harbin Institute of Technology, Shenzhen 518055, China.

ozerov@chem.tamu.edu, jiazhou@hit.edu.cn

Table of Contents

1. General Considerations. S3

2. DFT Calculations and Results S5

3. X-Ray Structural Determination Details. S9

4. Synthesis and Characterization of Ir Compounds. S10

5. Synthesis of Rh Compounds and Thermolysis Observations. S15

6. Kinetic and Mechanistic Studies with Ir. S24

6.1. Van't Hoff Analysis of Pyridine-Boron Binding S24

6.2. Experiments Analyzing the Thermolysis of **3**. S28

6.2.1. Rate Law Study S28

6.2.2. KIE Studies in the Thermolysis of **3**. S33

6.2.3. Eyring Study for the Thermolysis of **3**. S34

6.3. Experiments Analyzing the Thermolysis of **9**. S36

6.4. Experiments Examining the Product Determining Step S40

6.4.1. Equilibrium Study. S40

6.4.2. Competition Study. S42

6.5. In Situ Analysis of the Thermolysis of **3** in Benzene. S44

6.6. Experiments Analyzing the Thermolysis of **2**, **2a**, and **2d**. S53

6.6.1. Rate Law and KIE study S53

6.6.2. Eyring Study of the Thermolysis of **2a** with DMAP. S57

7. Kinetic Studies with Rh Compounds. S61

7.1. Rate determination for the thermolysis of **3-Rh**. S61

7.2. H/D Exchange Experiments. S63

7.3. KIE Experiments S67

8. Additional NMR Spectra for Ir Compounds. S71

9. Additional NMR Spectra for Rh Compounds. S86

10. SI References S99

1. General Considerations.

Unless otherwise specified, all manipulations were performed under an atmosphere of argon using either a glovebox or Schlenk techniques. Toluene, pentane, diethyl ether (Et_2O), isooctane, and tetrahydrofuran (THF) solvents were dried and deoxygenated (by purging) using a solvent purification system (Innovative Technology Pure Solv MD-5 Solvent Purification System). Pyridine, pyridine-d_5, 4-trifluoromethyl pyridine, 2-methyl pyridine, 3-fluoro pyridine, n-butyl ether, mesitylene, benzotrifluoride, benzene-d_6 (C_6D_6) toluene-d_8 (C_7D_8), cyclooctane, mesitylene, dimethoxyethane (DME), quinoline, Ph_2O, and 4-dimethylaminopyridine (DMAP) were dried over calcium hydride, vacuum transferred, and stored over molecular sieves in an Ar-filled glovebox. Celite and silica were dried at 180 °C under vacuum overnight and stored in an Ar-filled glovebox. Iridium compounds **1, 2, 2a, 2d, 2e, 3**, **5** and **17** were all prepared and identified according to previously published procedures.[1,2] The experimental section (below) reports NMR data in a new solvent (C_7D_8). Rhodium compounds **2-Rh** and **8-Rh** and were also synthesized according to literature precedent.[3,4] All other chemicals were used as received from commercial vendors. All NMR spectra were recorded on a Varian Inova 500 NMR spectrometer (^{1}H NMR, 500 MHz; ^{13}C{^{1}H} NMR, 126 MHz; ^{31}P{^{1}H} NMR, 202 MHz; ^{19}F NMR, 470 MHz), a Varian VnmrS 500 NMR spectrometer (^{1}H NMR, 500 MHz; ^{13}C{^{1}H} NMR, 126 MHz; ^{31}P{^{1}H} NMR, 202 MHz; ^{19}F NMR, 470 MHz; ^{11}B{^{1}H} NMR, 160 MHz; ^{2}H NMR, 77 MHz), Bruker Avance Neo 400 (^{1}H NMR, 400 MHz; ^{13}C NMR, 101 MHz; ^{31}P NMR, 162 MHz; ^{11}B NMR, 128 MHz), and Bruker Avance Neo 500 (^{1}H NMR, 500 MHz; ^{2}H NMR, 77 MHz; ^{13}C NMR, 126 MHz; ^{31}P NMR, 202 MHz; ^{11}B NMR, 161 MHz) spectrometers. All spectra were recorded at ambient temperature unless otherwise noted. Chemical shifts are reported in δ (ppm). For ^{1}H NMR spectra, the residual solvent peak was used as an internal reference (C_6D_6 at δ 7.16; C_7D_8 at δ 2.08 or 6.97).

$^{13}C\{^{1}H\}$ NMR spectra were internally referenced (C_6D_6 at δ 128.06; C_7D_8 at 20.43). $^{11}B\{^{1}H\}$ NMR spectra were referenced externally using neat $BF_3 \cdot OEt_2$ at δ 0, $^{31}P\{^{1}H\}$ NMR spectra were referenced externally using 85% phosphoric acid at δ 0, and ^{19}F NMR spectra were referenced internally (benzotrifluoride, ^{19}F NMR chemical shift at -63.7 ppm) or externally using neat trifluoroacetic acid at -78.5 ppm. Errors for rate constants were defined as double the standard deviation of the slope provided by the LINEST function in Microsoft Excel. Error for other values were calculated by doubling the value obtained from error propagation formulas, unless otherwise noted. Elemental analysis was performed by Robertson Microlit Laboratories, Ledgewood NJ.

2. DFT Calculations and Results.

The Gaussian suite of programs[5] was used for the ab initio electronic structure calculations. All structures were fully optimized by the B97D3[6] functional in the gas phase, and harmonic vibrational frequency calculations were performed to ensure that a minimum was obtained unless stated otherwise. The Los Alamos basis set and the associated effective core potential (ECP) was used for Ir and Rh atoms, and an all-electron 6-31G(d) basis set was used for all the other atoms. The solvation energies in benzene at room temperature were calculated on the B97D3-optimized geometries via the SMD model with the double hybrid dispersion-corrected functional, B2PLYP-D3, and the SDD/6-311+G(d,p) basis set.[6,7]

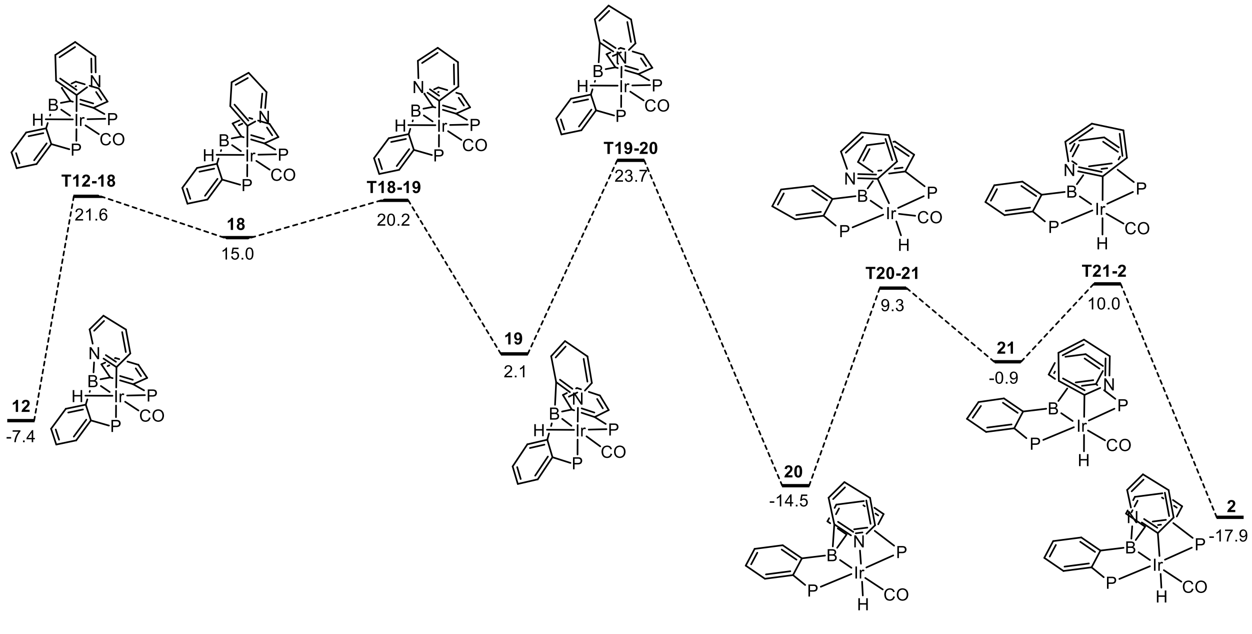

Figure S1. DFT-calculated pathway for the isomerization **12** into **2** via the migration of $C_{pyridyl}$ from Ir to B.

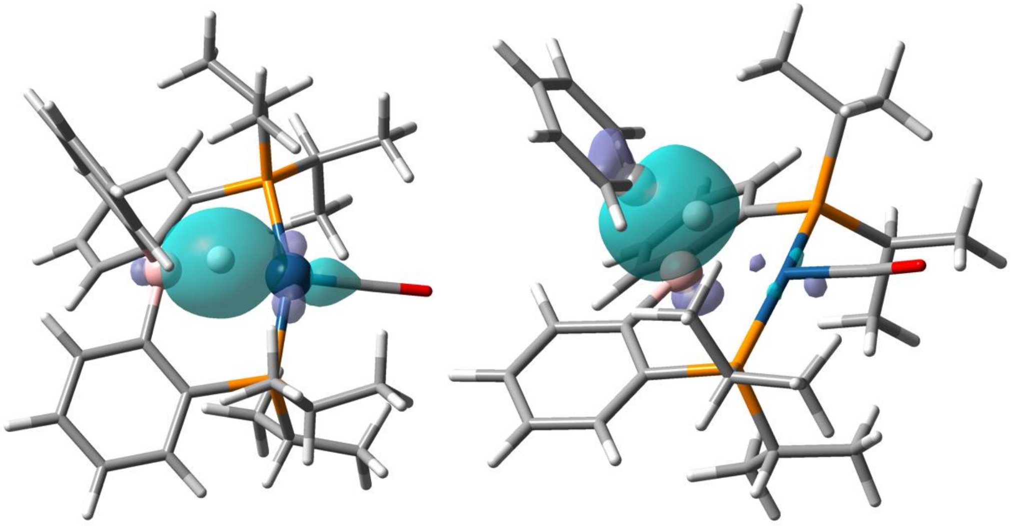

Figure S2. Representation of MOs involving bridging hydride for **3** (left) and **T3-4** (right). In **3**, it is a clear 3-center-2-electron bond with ca. 22% B, 35% H and 31% Ir contribution. In **T3-4**, it shows a strong C/B/H interaction, consisting of ca. 44% C, 18% B, 22% H.

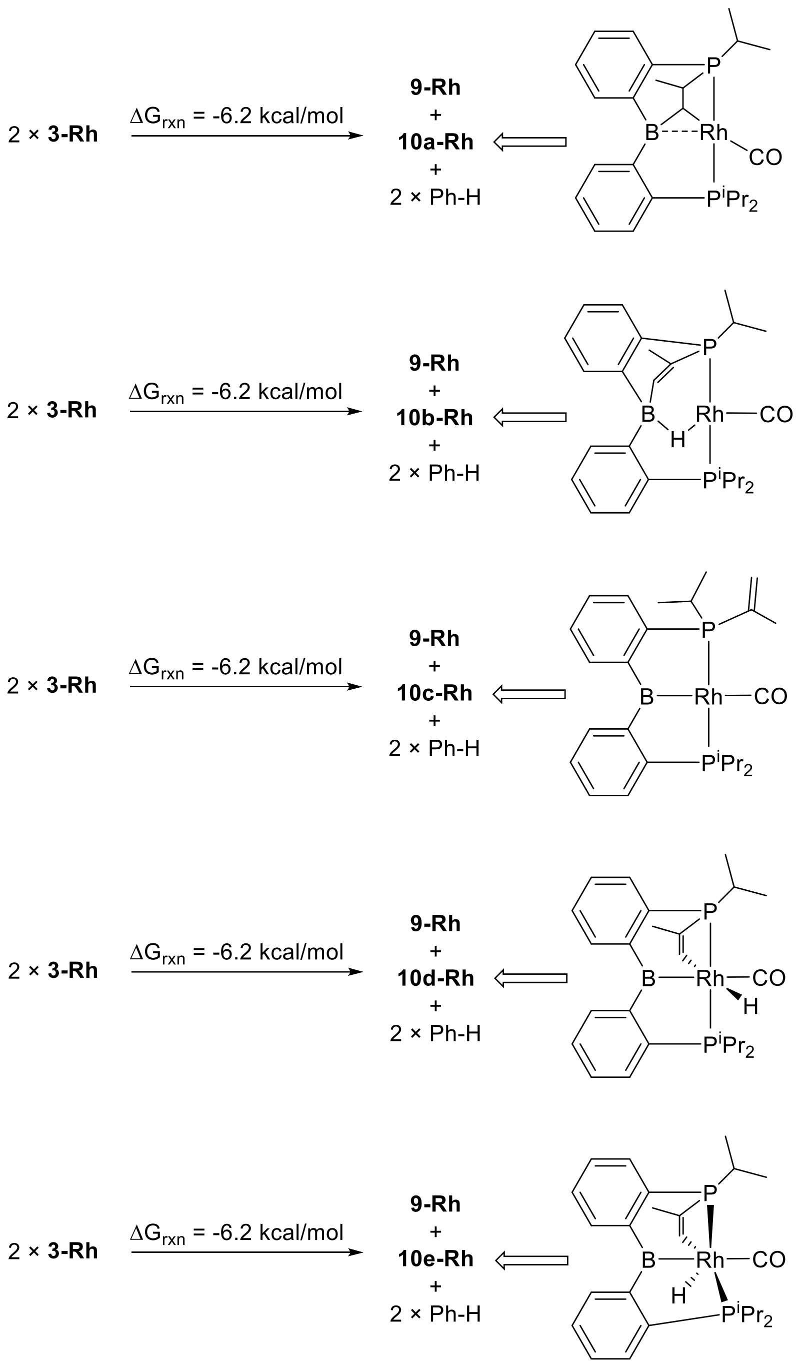

Figure S3. Selected candidates for **10-Rh** whose structure was optimized by DFT methods and the corresponding calculated energies of their putative formation.

Table S1. Gibbs energy values (in kcal/mol) for the Ir and Rh compounds calculated (relative to **4** or **4-Rh** at zero) using the following methods (Method 1 is used in the main text):

1) SMD(Bz)-B2PLYPD3/SDD/6-311+G(d,p)//B97D3/LANL2DZ/6-31G(d);
2) SMD(Bz)-B97D3/SDD/6-311+G(d,p)//B97D3/LANL2DZ/6-31G(d);
3) SMD(Bz)-B97D3/LANL2DZ/6-31G(d)//B97D3/LANL2DZ/6-31G(d);
4) SMD(Bz)-B97D3/LANL2DZ/6-31G(d)//SMD(Bz)-B97D3/LANL2DZ/6-31G(d)

Compound	Method 1	Method 2	Method 3	Method 4
**3**	0.5	1.1		
**T3-7**	28.7	34.1	33.4	33.0
**T3-7-Rh**	37.0		42.9	42.9
**7**	18.3	23.7		
**7-Rh**	30.6			
**T7-4**	25.8	30.8		
**T7-4-Rh**	30.7			
**4**	0.0	0.0		
**4-Rh**	0.0		0.0	0.0
**T4-11**	5.5	6.2		
**11**	-3.4	-0.5		
**T11-12**	9.8	17.0		
**12**	-7.4	0.5		
**T3-4**	29.9	28.9	28.1	27.8
**T3-4-Rh**	29.9		28.4	28.4
**1**	-23.3	-19.4		
**16**	-23.6	-17.4		
**T12-2**	15.5	23.0		
**2**	-17.9	-9.1		
**14**	20.2	25.4		
**T12-18**	21.6	27.1		
**18**	15.0	20.2		
**T18-19**	20.2	27.0		
**19**	2.1	9.6		
**T19-20**	23.7	29.2		
**20**	-14.5	-6.3		
**T20-21**	9.3	16.4		
**21**	-0.9	5.2		
**T21-2**	10.0	16.2		
**2**	-17.9	-9.1		
**15**	10.4	17.4		

3. X-Ray Structural Determination Details.

X-Ray data collection, solution, and refinement for 9-Rh (CCDC 2380713). A Leica M80 microscope was used to identify a suitable single colorless block-shaped crystal of **9-Rh** showing well defined faces with dimensions 0.26 × 0.14 × 0.08 mm^3 from a representative sample of crystals of the same habit. The crystal mounted on a nylon loop was then placed in a cold nitrogen stream (Oxford) maintained at T = 100.00(10) K. Crystal screening, unit cell determination, and data collection were carried out using a XtaLAB Synergy, Dualflex, HyPix diffractometer. The diffraction pattern was indexed and the total number of runs and images was based on the strategy calculation from the program CrysAlisPro system (CCD 42.101a 64-bit (release 31-08-2023)).[8] Data were measured using ω scans with Cu K_α radiation. Data was collected to a maximum resolution of Θ = 80.014° (0.78 Å). The unit cell was refined using CrysAlisPro 1.171.43.68a on 26507 reflections, 95% of the observed reflections. Integrated Intensity information for each reflection was obtained by reduction of data frames using CrysAlisPro 1.171.43.68a.[8] The final completeness is 100.00 % out to 80.014° in Θ. A gaussian absorption correction was performed using CrysAlisPro 1.171.43.68a.[8] Numerical absorption correction based on gaussian integration over a multifaceted crystal model Empirical absorption correction using spherical harmonics, implemented in SCALE3 ABSPACK scaling algorithm. The absorption coefficient μ of this material is 6.730 mm^{-1} at this wavelength (λ = 1.54184Å) and the minimum and maximum transmissions are 0.101 and 0.786. Systematic reflection conditions and statistical tests of the data suggested the space group P21 (# 4) and was confirmed by ShelXT structure solution program using dual methods.[9] The structure was refined by full matrix least squares minimization on F2 using version 2018/3 of ShelXL.[9] All non-hydrogen atoms were refined anisotropically. Hydrogen atom positions were calculated geometrically and refined using the riding model.

4. Synthesis and Characterization of Ir Compounds.

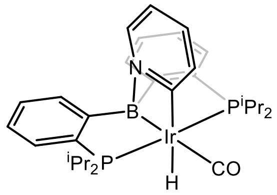

2

C_7D_8 NMR data for 2. ^{1}H NMR (500 MHz, C_7D_8): δ 8.43 (d, $J_{H,H}$ = 7.5 Hz, 2H, Ar-*H*), 7.54 (d, $J_{H,H}$ = 7.6 Hz, 1H, Py-*H*), 7.29 (m, 2H, Ar-*H*), 7.19 (d, $J_{H,H}$ = 5.3 Hz, 1H, Py-*H*), 7.09 (m, 4H, Ar-*H*), 6.48 (t, $J_{H,H}$ = 7.7 Hz, 1H, Py-*H*), 5.90 (t, $J_{H,H}$ = 6.6 Hz, 1H, Py-*H*), 2.05 (m, 2H, C*H*Me$_2$), 1.99 (m, 2H, C*H*Me$_2$), 1.21 (m, 12H, CH*Me$_2$*), 0.78 (dvt, $J_{H,P} \approx J_{H,H}$ = 7.5 Hz, 6H, CH*Me$_2$*), 0.28 (dvt, $J_{H,P} \approx J_{H,H}$ = 7.5 Hz, 6H, CH*Me$_2$*), -14.27 (t, $J_{H,P}$ = 19.3 Hz, Ir-*H*).

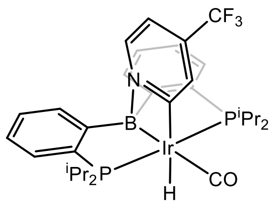

2a

Synthesis of 2a. In a 25-mL Schlenk flask, 226 mg (0.32 mmol) of **3** was dissolved in 4 mL of toluene and 39 µL (0.34 mmol) of 4-trifluoro methyl pyridine. The reaction mixture was heated at 100 °C for 2.5 h before filtering through a pad of Celite. The filtrate was evaporated to dryness to yield **3** as a light-yellow solid (170 mg, 66% yield). ^{1}H NMR (500 MHz, C_7D_8): δ 8.40 (d, $J_{H,H}$ = 7.8 Hz, 2H, Ar-*H*), 8.06 (s, 1H, Py-*H*), 7.31 (t, 2H, $J_{H,H}$ = 7.3 Hz, Ar-H), 7.11 (m, 3H), 7.05 (m, 2H), 6.07 (d, 1H, $J_{H,H}$ = 6 Hz, Py-*H*), 2.01 (m, 2H, C*H*Me$_2$), 1.92 (m, 2H, C*H*Me$_2$), 1.13 (m, 12H, CH*Me$_2$*), 0.73 (dvt, $J_{H,P} \approx J_{H,H}$ = 7 Hz, 6H, CH*Me$_2$*), 0.23 (dvt, $J_{H,P} \approx J_{H,H}$ = 7 Hz, 6H, CH*Me$_2$*), -14.37 (t, $J_{H,P}$ = 18.9 Hz, 1H, Ir-*H*). ^{13}C{^{1}H} NMR (126 MHz, C_7D_8): δ 178.2 (s, CO), 170.1 (s, Ir-C), 162.9 (br s, B-C), 146.7 (vt, $J_{C,P}$ = 26.4 Hz, C-P), 142.3 (s), 134.5 (s), 134.2 (q, $J_{C,F}$ = 32.6 Hz, *C*-CF$_3$), 131.8 (m), 129.9 (s), 129.3 (s), 126.3 (t, $J_{C,P}$ = 3.9 Hz), 123.6 (q, $J_{C,F}$ = 274 Hz, *C*-F), 110.9 (m), 31.3 (vt, $J_{C,P}$ = 13.6 Hz, *C*HMe$_2$), 30.8 (vt, $J_{C,P}$ = 19.0 Hz, *C*HMe$_2$), 20.3 (vt, $J_{C,P}$ = 2.2 Hz, CH*Me$_2$*), 19.7 (s, CH*Me$_2$*), 19.1 (s, CH*Me$_2$*). ^{31}P{^{1}H} NMR (202 MHz, C_7D_8): δ 56.0. ^{19}F NMR (470 MHz, C_7D_8): δ -65.9.

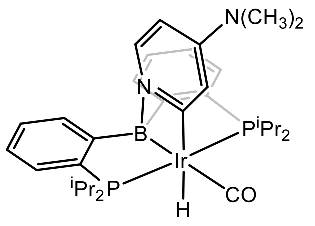

2b

C_7D_8 NMR data for 2b. ^{1}H NMR (500 MHz, C_7D_8): δ 8.50 (d, $J_{H,H}$ = 7.5 Hz, 2H, Ar-*H*), 7.32 (t, 2H, $J_{H,H}$ = 7.3 Hz, Ar-H), 7.14 (m, 4H, Ar-H), 6.98 (d, 1H, $J_{H,H}$ = 7 Hz, Py-H), 6.93 (s, 1H, Py-H), 5.30 (m, 1H, Py-H), 2.19 (s, 6H, N*Me2*), 2.09 (m, 4H, C*H*Me2), 1.34 (dvt, $J_{H,P}$ ≈ $J_{H,H}$ = 7 Hz, 6H, CH*Me2*), 1.24 (dvt, $J_{H,P}$ ≈ $J_{H,H}$ = 7 Hz, 6H, CH*Me2*), 0.84 (dvt, $J_{H,P}$ ≈ $J_{H,H}$ = 7 Hz, 6H, CH*Me2*), 0.53 (dvt, $J_{H,P}$ ≈ $J_{H,H}$ = 7 Hz, 6H, CH*Me2*), -14.17 (t, $J_{H,P}$ = 19.1 Hz, 1H, Ir-*H*).

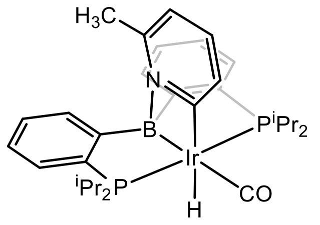

2c

Synthesis of 2c. To a J. Young NMR tube, 17 mg (0.02 mmol) of **3** was dissolved in 560 µL of C_7D_8 and 2 µL (0.02 mmol) of 2-methyl pyridine. After heating at 100 ºC in an oil bath for 1.5 h, *in situ* ^{31}P{^{1}H} NMR spectroscopy indicated **2c** was formed in >99% purity. Benzene was also present as a product of thermolysis of **3**. NMR values presented are for **2c** as part of an *in situ* J. Young NMR tube reaction mixture. ^{1}H NMR (500 MHz, C_7D_8): δ 8.56 (d, $J_{H,H}$ = 7.5 Hz, 2H, Ar-*H*), 7.54 (d, $J_{H,H}$ = 7.2 Hz, 1H, Py-H), 7.26 (t, 2H, $J_{H,H}$ = 6.5 Hz, Ar-H), 7.09 (m, 4H, Ar-H), 6.50 (t, 1H, $J_{H,H}$ = 7.2 Hz, Py-H), 5.81 (d, 1H, $J_{H,H}$ = 7.2 Hz, Py-H), 2.01 (s, 3H, Ar-*Me*), 1.99 (m, 4H, C*H*Me2), 1.26 (dvt, $J_{H,P}$ ≈ $J_{H,H}$ = 7 Hz, 6H, CH*Me2*), 1.20 (dvt, $J_{H,P}$ ≈ $J_{H,H}$ = 7 Hz, 6H, CH*Me2*), 0.72 (dvt, $J_{H,P}$ ≈ $J_{H,H}$ = 7 Hz, 6H, CH*Me2*), 0.35 (dvt, $J_{H,P}$ ≈ $J_{H,H}$ = 7 Hz, 6H, CH*Me2*), -13.92 (t, $J_{H,P}$ = 18.8 Hz, 1H, Ir-*H*). ^{31}P{^{1}H} NMR (202 MHz, C_7D_8): δ 54.5.

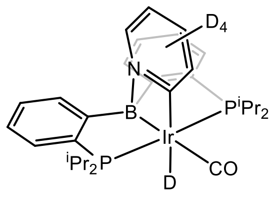

2d

Synthesis of 2d. In a 25-mL Schlenk flask, 120 mg (0.17 mmol) of **3** was dissolved in 4 mL of toluene and 20 µL (0.17 mmol) pyridine-d_5. The reaction mixture was heated at 100 ºC for 3 h before filtering through a pad of Celite. The filtrate was evaporated to dryness to yield **2d** as a light-yellow solid (85 mg, 67% yield). ^{1}H NMR (500 MHz, C_6D_6): δ 8.50 (d, $J_{H,H}$ = 7.7 Hz, 2H), 8.34 (br s, 2H), 7.13 (br s, 4H, $J_{H,H}$ = 7.3 Hz), 2.10 (m, 2H, C*H*Me$_2$), 2.00 (m, 2H, C*H*Me$_2$), 1.25 (m, 12H, CH*Me$_2$*), 0.84 (dvt, $J_{H,P} \approx J_{H,H}$ = 7 Hz, 6H, CH*Me$_2$*), 0.34 (dvt, $J_{H,P} \approx J_{H,H}$ = 7 Hz, 6H, CH*Me$_2$*).

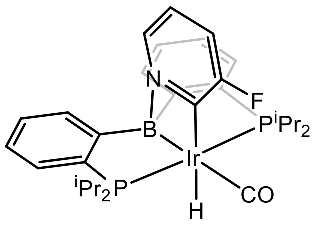

2e

Synthesis of 2e. To a J. Young NMR tube, 25 mg (36 mmol) of **3** was dissolved in 600 µL of C_7D_8 and 3 µL of 3-fluoropyridine (37 mmol). After heating at 100 ºC for 1.5 h, *in situ* ^{31}P{^{1}H} NMR spectroscopy indicated **2e** was formed in >96% purity. Benzene chemical signals are also present as a product of thermolysis of **3**. NMR values presented are for **2e** as part of the *in situ* J. Young NMR tube reaction mixture. ^{1}H NMR (500 MHz, C_7D_8): δ 8.42 (d, $J_{H,H}$ = 7.7 Hz, 2H, Ar-*H*), 7.29 (t, $J_{H,H}$ = 7.2 Hz, 2H, Ar-*H*), 7.08 (m, 5H), 6.26 (m, 1H, Py-*H*), 5.75 (dt, $J_{H,F}$ = 6 Hz, $J_{H,H}$ = 5 Hz, 1H, Py-*H*), 2.04 (m, 2H, C*H*Me$_2$), 1.94 (m, 2H, C*H*Me$_2$), 1.27 (dvt, $J_{H,H} \approx J_{H,P}$ = 7 Hz, 6H, CH*Me$_2$*), 1.18 (dvt, $J_{H,H} \approx J_{H,P}$ = 7 Hz, 6H, CH*Me$_2$*), 0.76 (dvt, $J_{H,H} \approx J_{H,P}$ = 7 Hz, 6H, CH*Me$_2$*), 0.26 (dvt, $J_{H,H} \approx J_{H,P}$ = 7 Hz, 6H, CH*Me$_2$*), -14.36 (td, $J_{H,F}$ = 6 Hz, $J_{H,P}$ = 18.7 Hz, 1H, Ir-*H*). ^{13}C{^{1}H} NMR (126 MHz, C_7D_8): δ 177.6 (s, *C*O), 166.9 (d, $J_{C,F}$ = 238 Hz, *C*-F), 163.3 (br s, B-*C*), 154.3 (br d, $J_{C,F}$ = 65.1 Hz, Ir-*C*), 147.2 (vt, $J_{C,P}$ = 26.5 Hz, *C*-P), 138.7 (s), 131.8 (m), 129.8 (s), 129.2 (s), 128.5 (s), 126.1 (t, $J_{C,P}$ = 3.9 Hz), 117.0 (d, $J_{C,F}$ = 24.1 Hz), 116.3 (d, $J_{C,F}$ = 4.8 Hz), 31.2 (vt, $J_{C,P}$ = 18.9 Hz, *C*HMe$_2$), 31.0

(vt, $J_{C,P}$ = 13.8 Hz, *C*HMe$_2$), 20.8 (vt, $J_{C,P}$ = 3 Hz), 20.4 (s), 19.6 (s), 19.2 (s). ^{31}P{^{1}H} NMR (202 MHz, C_7D_8): δ 51.0. ^{19}F NMR (471 MHz, C_7D_8): δ -110.6 (q, $J_{F,H} \approx J_{F,P}$ = 6 Hz).

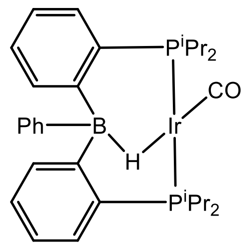

3

C_7D_8 NMR data for 3. ^{1}H NMR (500 MHz, C_7D_8): δ 7.53 (d, $J_{H,H}$ = 7.6 Hz, 2H), 7.12 (m, 6H), 7.05 (m, 5H), 2.43 (m, 2H, C*H*Me$_2$), 2.17 (m, 2H, C*H*Me$_2$), 1.07 (m, 18H, CH*Me$_2$*), 0.91 (vtd, $J_{H,P} \approx J_{H,H}$ = 7 Hz, 6H, CH*Me$_2$*), -4.20 (br, Ir-*H*).

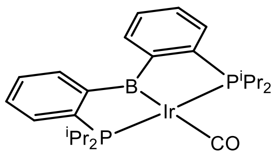

6 (*in situ*)

***In situ* observation of 4.** In an Ar-filled glovebox, 25 mg **3** (36 mmol) was dissolved in 600 µL C_6D_6 in a J. Young NMR tube. The sample was heated at 100 ºC for 2 h in an oil bath, resulting in a dark brown mixture. The sample was analyzed via ^{1}H{^{31}P}, ^{13}C{^{1}H}, and ^{31}P{^{1}H} NMR spectroscopy (see Figure S48, Figure S49, and Figure S50, respectively). Selected NMR data for **4** follow: ^{1}H{^{31}P} NMR (500 MHz, C_6D_6): δ 8.02 (m, 4H), 7.26 (m, 4H), 2.43 (m, 4H, C*H*Me$_2$), 1.00 (d, $J_{H,H}$ = 7 Hz, 24H, CH*Me$_2$*). ^{13}C{^{1}H} NMR (126 MHz, C_6D_6): 210.0 (s, CO), 164.5 (br s), 152.3 (vt, J_{CP} = 24 Hz), 130.8 (s), 130.2 (s), one C_{Ar} signal is likely overlapping with solvent or other *in situ* product signals. ^{31}P{^{1}H} NMR (202 MHz, C_6D_6): δ 81.7.

In this experiment, the intensity of the Ir-H and Ph-derived ^{1}H NMR resonances for **1**, **3**, **17** were either diminished or not detected (extensive overlap makes it difficult to ascertain this for all of the aromatic resonances). Likewise, in the ^{13}C{^{1}H} NMR spectra, the Ph-derived resonances were diminished or not detected because of lower S/N ratio. These observations are consistent with the dominant presence of C_6D_5 and Ir-D groups derived from the exchange with C_6D_6.

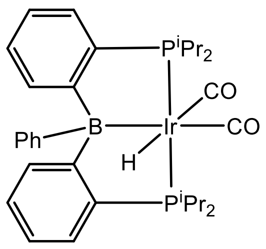

9

C_7D_8 NMR data for 5. ^{1}H NMR (500 MHz, C_7D_8): δ 7.68 (d, $J_{H,H}$ = 7.6 Hz, 2H), 7.13 (t, $J_{H,H}$ = 7.6 Hz, 2H), 7.09 (m, 2H), 7.05 (m, 2H), 6.99 (t, $J_{H,H}$ = 7.8 Hz, 2H), 6.87 (t, $J_{H,H}$ = 7.6 Hz, 1H), 6.76 (d, $J_{H,H}$ = 7.4 Hz, 2H), 2.34 (m, 2H, C*H*Me$_2$), 2.25 (m, 2H, C*H*Me$_2$), 1.14 (dvt, $J_{H,H} \approx J_{H,P}$ = 7 Hz, 6H, CH*Me$_2$*), 0.99 (m, 12H, CH*Me$_2$*), 0.67 (dvt, $J_{H,H} \approx J_{H,P}$ = 7 Hz, 6H, CH*Me$_2$*), -10.76 (t, $J_{H,P}$ = 15.1 Hz, 1H, Ir-*H*). ^{31}P{^{1}H} NMR (202 MHz, C_7D_8): δ 51.8.

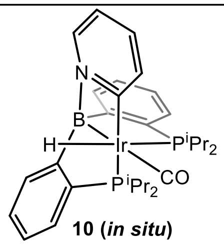

10 (*in situ*)

***In situ* observation of 12.** ^{1}H NMR (500 MHz, C_6D_6): δ 8.41 (m, 1H), 8.01 (m, 2H), 7.65 (m, 2H), 7.6 (m, 2H), 6.51 (t, 1H, $J_{H,H}$ = 8 Hz), 6.01 (t, 1H, $J_{H,H}$ = 7 Hz), 1.98 (m, 4H, C*H*Me$_2$), 1.44 (m, 6H), 1.35 (m, 12H, CH*Me$_2$*), 0.23 (m, 6H, CH*Me$_2$*), -10.29 (d, $J_{H,P}$ = 29 Hz, 108 Hz, 1H, Ir-H). 3H are indistinguishable from the *in situ* sample. ^{31}P{^{1}H} NMR (202 MHz, C_6D_6): δ 37.0 ($J_{P,P}$ = 19 Hz), 31.2 ($J_{P,P}$ = 19 Hz). In an Ar-filled glovebox, 8.3 mg (0.01 mmol) of **3** and 600 µL C_6D_6 were introduced into a J. Young NMR tube. The sample was heated at 100 ºC for 15 h in an oil bath. Pyridine (1 µL, 0.01 mmol) was added via microsyringe to the reaction mixture, causing an immediate color change to orange-red. This sample was analyzed via ^{1}H and ^{31}P{^{1}H} NMR spectroscopy 10 min after pyridine addition. See Figures S51-53 for the NMR spectra.

5. Synthesis of Rh Compounds and Thermolysis Observations.

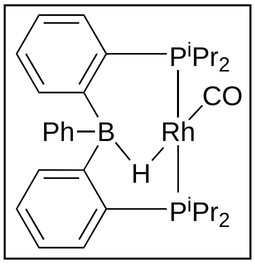

Synthesis of 3-Rh. In a 50 mL Schlenk flask, $NaBH_4$ (30 mg, 0.78 mmol) was added to a solution of **8-Rh** (500 mg, 0.78 mmol) in 8 mL DME. The mixture was stirred at room temperature for 1 h before filtering through a pad of Celite. The filtrate was evaporated to dryness affording a pale-yellow solid (402 mg, 85%). ^{1}H NMR (500 MHz, C_6D_6): δ 7.65 (d, $J_{H–H}$ = 7.4 Hz, 2H, Ar-*H*), 7.14 (m, 6H Ar-*H*, overlap with C_6D_6), 7.05 – 6.99 (m, 5H, Ar-*H*), 2.26 (m, 2H, C*H*Me$_2$), 2.11 (m, 2H, C*H*Me$_2$), 1.05 (m, 18H, CH*Me$_2$*), 0.90 (dvt, $J_{H–H} \approx J_{H–P}$ = 7.0 Hz, 6H, CH*Me$_2$*), -5.29 (br, 1H, Rh-*H*). ^{31}P{^{1}H} NMR (202 MHz, C_6D_6): δ 67.8 (d, $J_{P–Rh}$ = 115.3 Hz). ^{11}B{^{1}H} NMR (160 MHz, C_6D_6): δ -0.6 (br). ^{13}C{^{1}H} NMR (126 MHz, C_6D_6): δ 196.1 (dt, $J_{C–Rh}$ = 77.0 Hz, $J_{C,P}$ = 12.3 Hz, Rh-*C*O), 161.3 (br, B-C_{Ar}), 157.1 (br, B-C_{Ph}), 136.0 (vt, $J_{C–P}$ = 8.2 Hz, C_{Ar}), 135.1 (vt, $J_{C–P}$ = 21.0 Hz, C_{Ar}), 134.8 (s), 129.6 (s), 129.4 (vt, $J_{C–P}$ = 2.3 Hz), 127.5 (s), 125.5 (vt, $J_{C–P}$ = 3.5 Hz, *C*HMe$_2$), 125.3 (s), 27.6 (vt, $J_{C–P}$ = 11.7 Hz, *C*HMe$_2$), 24.1 (vt, $J_{C–P}$ = 13.1 Hz, *C*HMe$_2$), 20.0 (vt, $J_{C–P}$ = 2.8 Hz, CH*Me$_2$*), 19.2 (s, CH*Me$_2$*), 19.1 (vt, $J_{C–P}$ = 3.0 Hz, CH*Me$_2$*), 18.3 (s, CH*Me$_2$*). ATR-IR: ν_{CO} 1974 cm^{-1}. Anal. Calcd for $C_{31}H_{42}BOP_2Rh$: C, 61.41; H, 6.98; N, 0. Found: C, 61.27; H, 6.69; N, <0.10.

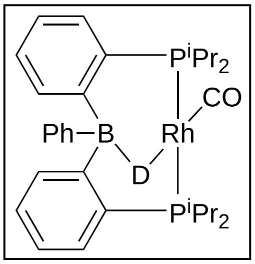

Synthesis of 3-Rh-d. In a 10-mL Schlenk flask, $NaBD_4$ (3.12 mL, 0.156 mmol, 0.050 M in DME) was added to a solution of **8-Rh** (190 mg, 0.156 mmol) in 2 mL DME. The mixture was stirred at room temperature for 1 h before filtering through a pad of Celite. The filtrate was evaporated to dryness affording a pale-yellow solid (147 mg, 82%). ^{1}H NMR (500 MHz, C_6D_6): δ 7.69 (d, $J_{H–H}$ = 7.5 Hz, 2H, Ar-*H*), 7.28 (m, 6H Ar-*H*, overlap with C_6D_6), 7.16 (m, 5H, Ar-*H*), 2.39 (m, 2H, C*H*Me$_2$), 2.25 (m, 2H, C*H*Me$_2$), 1.18 (m, 18H, CH*Me$_2$*), 1.04 (dvt, $J_{H–H} \approx J_{H–P}$ = 7.3 Hz, 6H,

CH*Me*$_2$). ^{2}H NMR (77 MHz, C_6D_6): δ -5.35 (s, Rh-*D*). ^{31}P{^{1}H} NMR (202 MHz, C_6D_6): δ 67.7 (d, $J_{P–Rh}$ = 115.4 Hz). ^{11}B{^{1}H} NMR (160 MHz, C_6D_6): δ -0.8 (br). ^{13}C{^{1}H} NMR (101 MHz, C_6D_6): δ 196.0 (dt, $J_{C–Rh}$ = 77.1 Hz, $J_{C–P}$ = 12.1 Hz, Rh-*C*O), 161.3 (br, B-C_{Ar}), 157.2 (br, B-C_{Ph}), 135.9 (vt, $J_{C–P}$ = 8.3 Hz, C_{Ar}), 135.1 (vt, $J_{C–P}$ = 21.1 Hz, C_{Ar}), 134.8 (s), 129.6 (s), 129.4 (s), 127.5 (s), 125.5 (vt, $J_{C–P}$ = 3.5 Hz, *C*HMe$_2$), 125.3 (s), 27.6 (vt, $J_{C–P}$ = 11.8 Hz, *C*HMe$_2$), 24.1 (vt, $J_{C–P}$ = 13.1 Hz, *C*HMe$_2$), 20.0 (vt, $J_{C–P}$ = 2.8 Hz, CH*Me*$_2$), 19.2 (s, CH*Me*$_2$), 19.1 (vt, $J_{C–P}$ = 3.11 Hz, CH*Me*$_2$), 18.3 (s, CH*Me*$_2$).

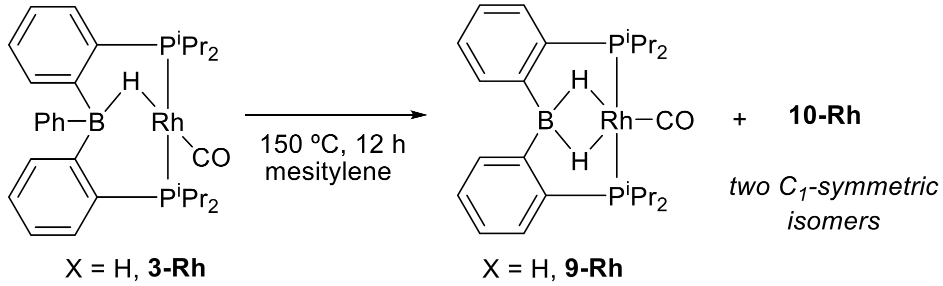

Thermolysis of 3-Rh. In a J. Young tube, a solution of **3-Rh** (50 mg, 0.083 mmol) in 600 µL mesitylene was heated at 150 ˚C for 12 h to afford a dark brown solution. Volatiles were removed and the residue redissolved in toluene-*d$_8$*, at which point ^{31}P{^{1}H} NMR analysis revealed the presence of **9-Rh** (ca. 50%), and two isomers of **10-Rh** (ca. 4:1 ratio) (Figure S4).

9-Rh: ^{31}P{^{1}H} NMR (162 MHz, toluene-*d$_8$*): δ 83.0 (d, $J_{P–Rh}$ = 108.3 Hz).

10-Rh (major isomer): ^{31}P{^{1}H} NMR (162 MHz, toluene-*d$_8$*): δ 65.4 (dd, $J_{P–P}$ = 275.1 Hz, $J_{P–Rh}$ = 134.4 Hz), 8.5 (dd, $J_{P–P}$ = 275.1 Hz, $J_{P–Rh}$ = 100.4 Hz).

10-Rh (minor isomer): ^{31}P{^{1}H} NMR (162 MHz, toluene-*d$_8$*): δ 65.6 (dd, $J_{P–P}$ = 275.9 Hz, $J_{P–Rh}$ = 136.8 Hz), -5.7 (dd, $J_{P–P}$ = 275.9 Hz, $J_{P–Rh}$ = 98.2 Hz).

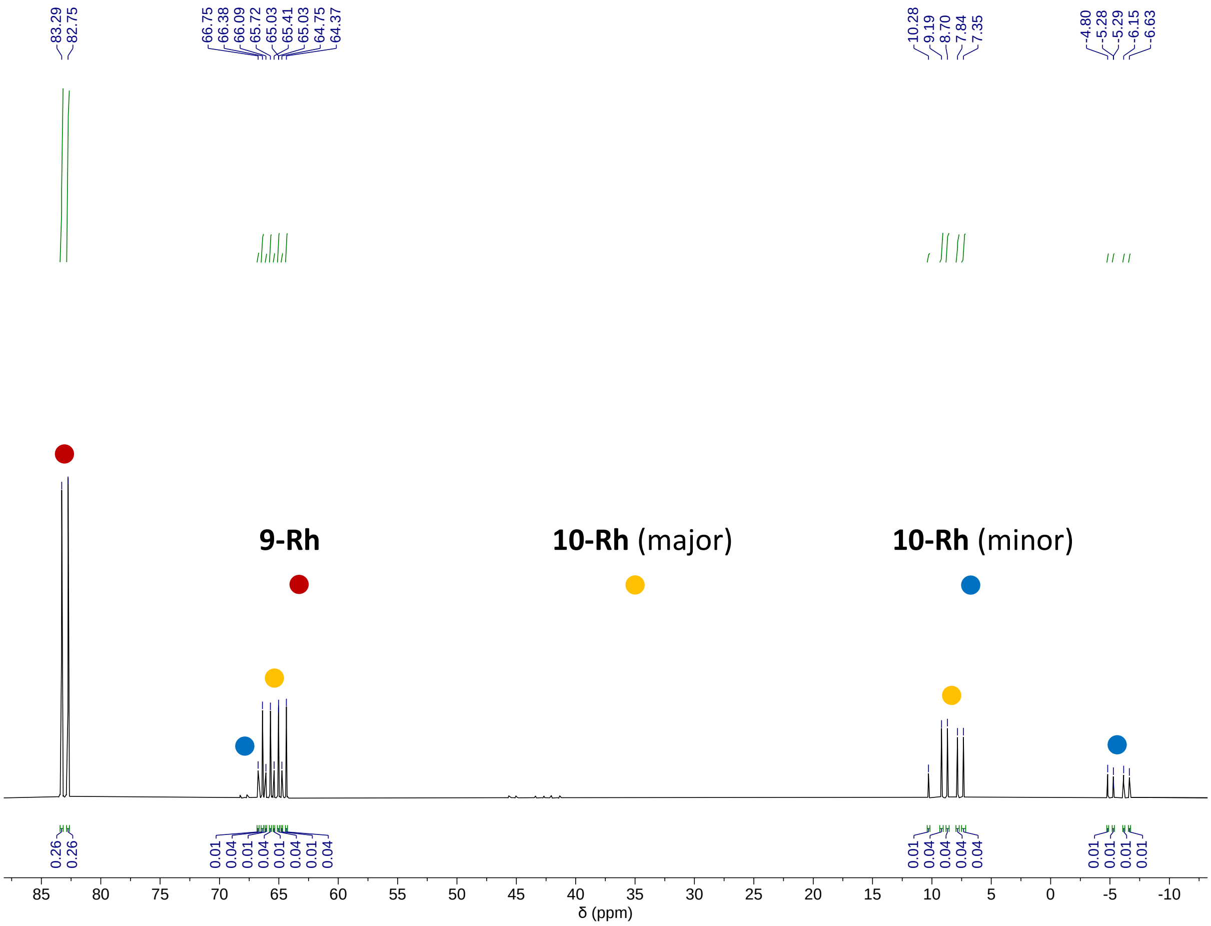

Figure S4. $^{31}P\{^{1}H\}$ NMR (202 MHz) spectrum for the thermolysis of **3-Rh** in toluene-d_8.

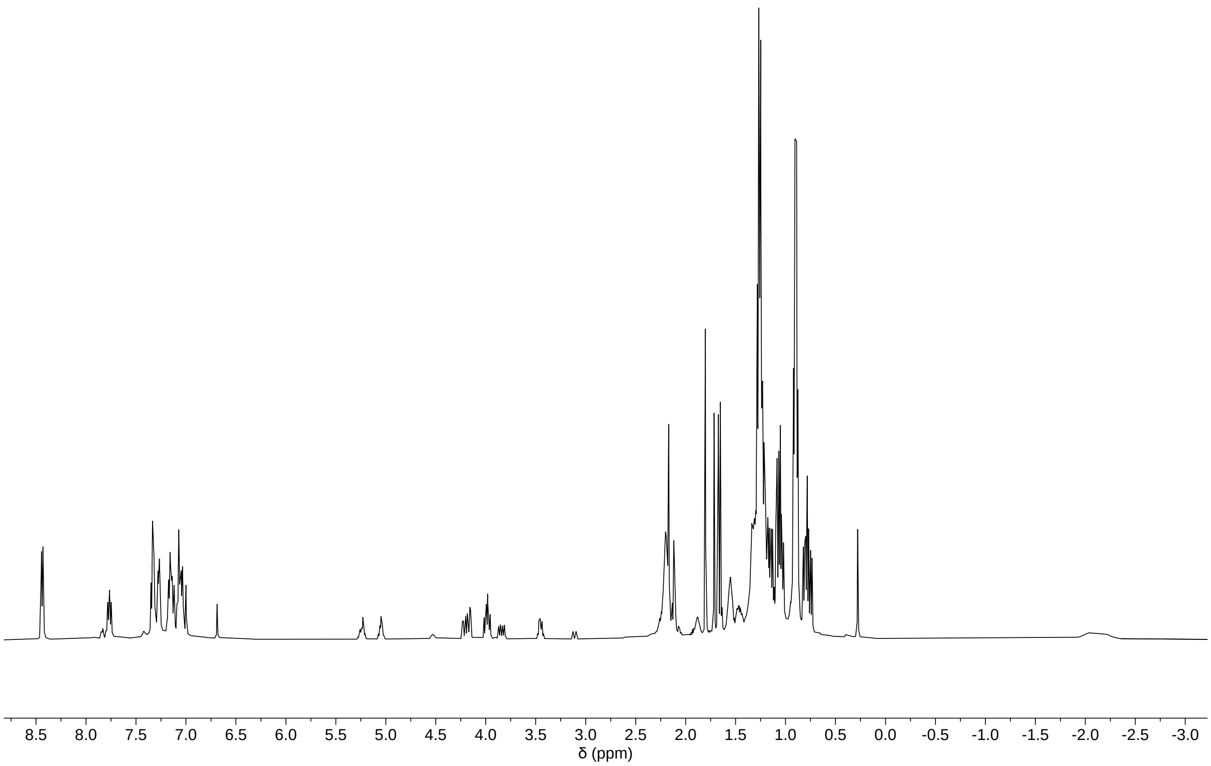

Figure S5. ^{1}H NMR (500 MHz) spectrum for the thermolysis of **3-Rh** in toluene-*d*$_8$.

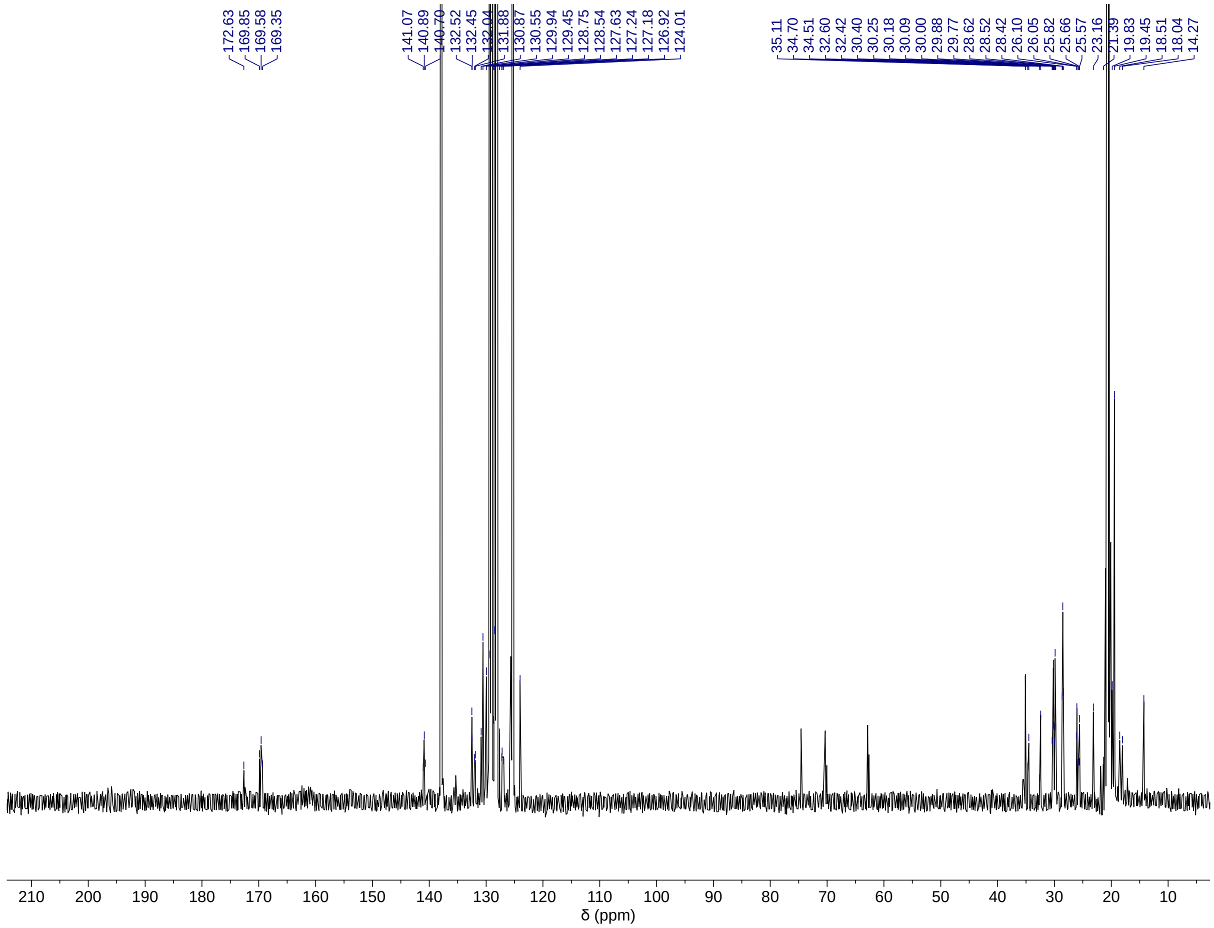

Figure S6. $^{13}C\{^{1}H\}$ NMR (126 MHz, 378 K) spectrum for thermolysis of **3-Rh** in toluene-d_8.

Thermolysis of 3-Rh with H_2. In a J. Young tube, 1 atm of H_2 was added to a solution of **1** (10 mg, 0.017 mmol) in 600 μL mesitylene after three rounds of freeze-pump-thaw. The resulting mixture was heated at 150 ˚C for 16 h. $^{31}P\{^1H\}$ NMR analysis revealed formation of **9-Rh** (Figure S7).

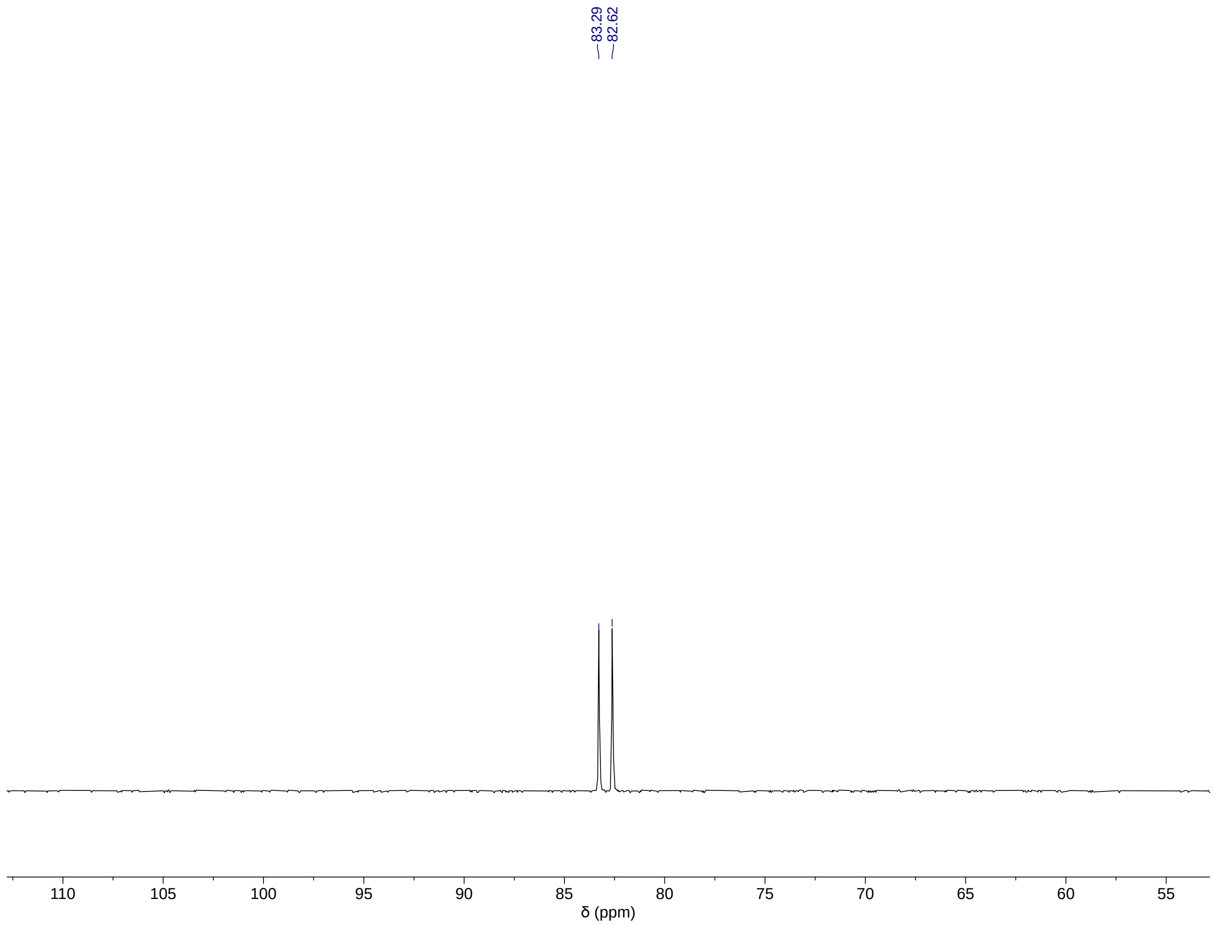

Figure S7. $^{31}P\{^1H\}$ NMR (162 MHz) spectrum for the thermolysis of **3-Rh** with H_2 in mesitylene (unlocked, unshimmed).

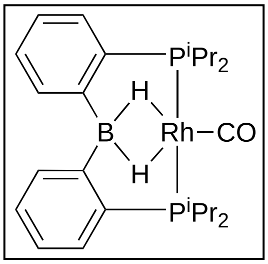

Synthesis of 9-Rh. In a 25-mL Schlenk flask, **3-Rh** (225 mg, 0.37 mmol) was heated at 150 ˚C for 24 h to afford a dark brown solid. After cooling to room temperature, the solid was recrystallized from pentane yielding bright yellow crystals (102 mg, 52%). ^{1}H NMR (400 MHz, C_6D_6): δ 8.53 (d, $J_{H–H}$ = 7.6 Hz, 2H, Ar-*H*), 7.36 (td, $J_{H–H}$ = 7.6 Hz, $J_{H–H}$ = 1.1 Hz, 2H, Ar-*H*), 7.28 (m, 2H, Ar-*H*), 7.08 (td, $J_{H–H}$ = 7.4 Hz, $J_{H,H}$ = 1.4 Hz), 2.20 (m, 4H, C*H*Me$_2$), 1.26 (dvt, $J_{H–H} \approx J_{H–P}$ = 6.9 Hz, 12H, CH*Me$_2$*), 1.04 (dvt, $J_{H–H} \approx J_{H–P}$ = 7.0 Hz, 12H, CH*Me$_2$*), -1.98 (br, 2H, B-*H*-Rh). ^{31}P{^{1}H} NMR (162 MHz, C_6D_6): δ 83.0 (d, $J_{P–Rh}$ = 108.3 Hz). ^{11}B{^{1}H} NMR (128 MHz, C_6D_6): δ -1.4 (br). ^{13}C{^{1}H} NMR (101 MHz, C_6D_6): δ 161.9 (br, B-C_{Ar}), 140.3 (vt, $J_{C–P}$ = 22.6 Hz, C_{Ar}), 132.3 (vt, $J_{C–P}$ = 8.6 Hz, C_{Ar}), 130.4 (s, C_{Ar}), 129.8 (s, C_{Ar}), 123.9 (vt, $J_{C–P}$ = 3.7 Hz, C_{Ar}), 27.9 (vt, $J_{C–P}$ = 12.8 Hz, *C*HMe$_2$), 20.3 (vt, $J_{C–P}$ = 2.9 Hz, CH*Me$_2$*), 19.9 (s, CH*Me$_2$*) (Rh–*C*O signal was not observed due to broadness). ATR-IR: ν_{CO} 1930 cm^{-1}.

Thermolysis of 3-Rh with DMAP. In a J. Young tube, DMAP (10 mg, 0.082 mmol) was added to a solution of **1** (50 mg, 0.082 mmol) in 600 µL mesitylene. The resulting mixture was heated at 150 ˚C for 6 h. $^{31}P\{^{1}H\}$ NMR analysis revealed PhH elimination and formation of complexes **9-Rh**, and two isomers of **10-Rh**, signifying no reaction with DMAP (Figure S8).

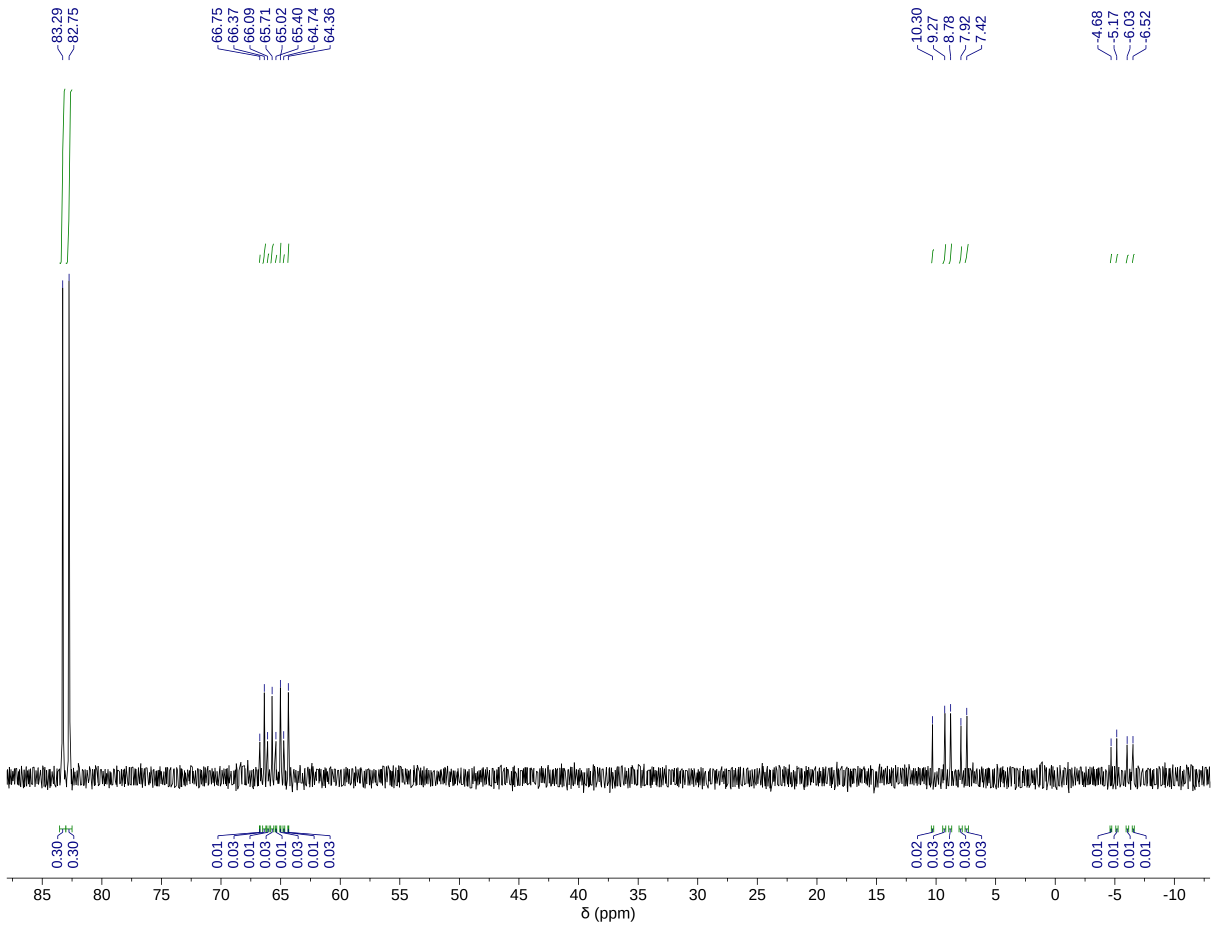

Figure S8. $^{31}P\{^{1}H\}$ NMR (202 MHz) spectrum for the thermolysis of **3-Rh** with DMAP in mesitylene (unlocked, unshimmed).

Thermolysis of 2-Rh/2'-Rh. In a J. Young tube, a solution of **2-Rh/2'-Rh** (20 mg, 0.035 mmol) in 600 µL Ph_2O/C_6D_6 (9:1) was heated at 150 ˚C for 1 h. $^{31}P\{^1H\}$ NMR analysis showed the formation of **9-Rh** and the two isomers of **10-Rh**, along with a few minor unidentified products (Figure S9).

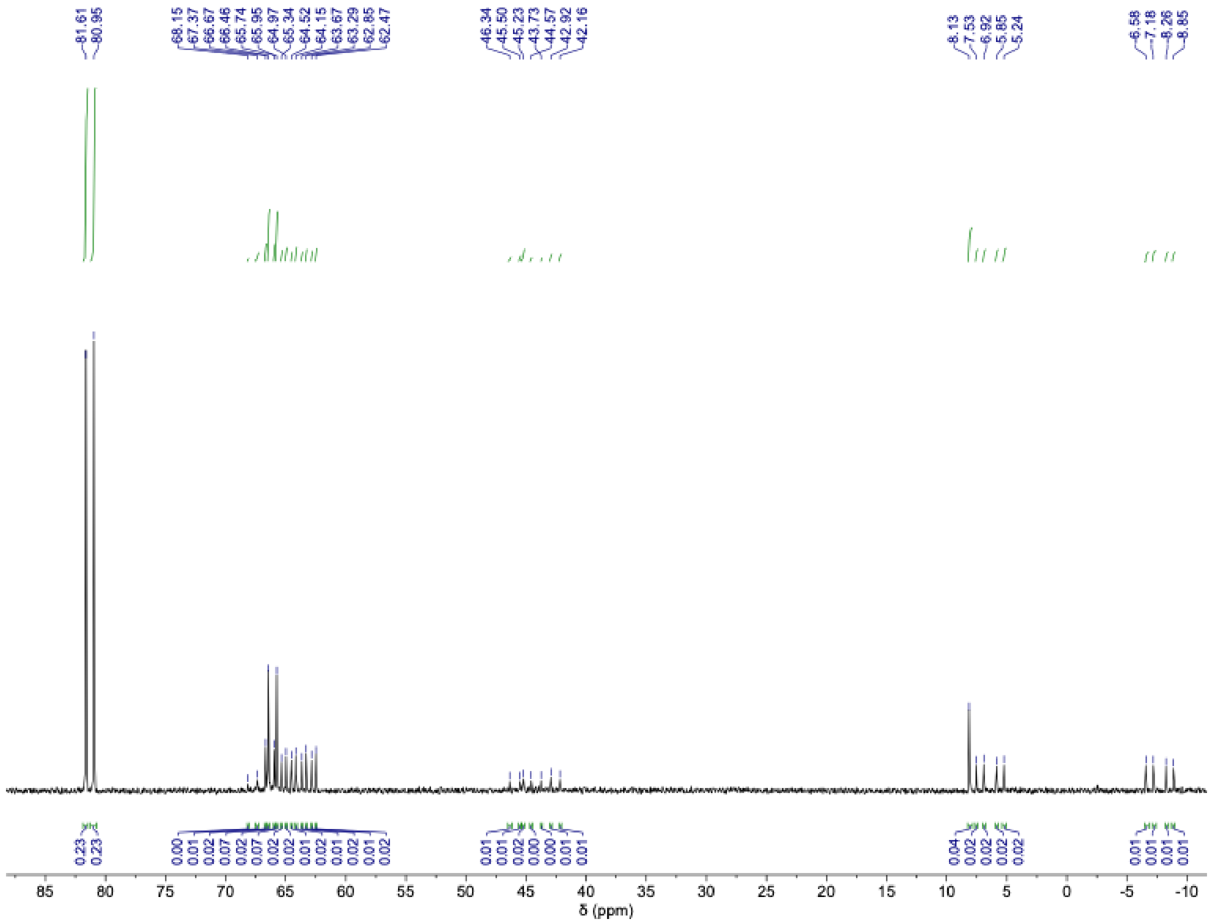

Figure S9. $^{31}P\{^1H\}$ NMR (162 MHz) spectrum for the thermolysis of **2-Rh/2'-Rh** in Ph_2O/C_6D_6 (9:1), (unlocked, unshimmed).

6. Kinetic and Mechanistic Studies with Ir.

6.1. Van't Hoff Analysis of Pyridine-Boron Binding

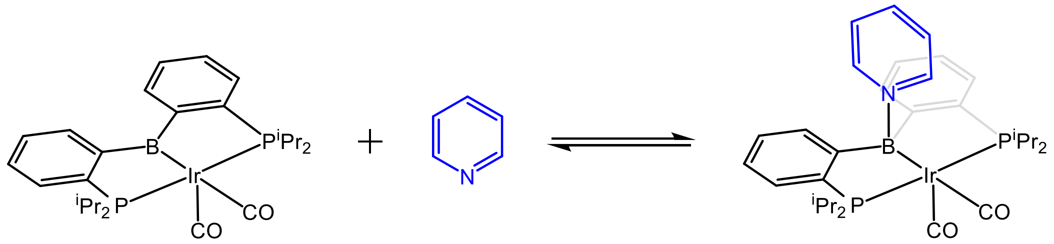

	**1**	**16**
$^{31}P\{^1H\}$:	61.0 ppm	54.7 ppm
$^{11}B\{^1H\}$:	99.4 ppm	20.3 ppm
ATR-IR ν_{CO}:	1960, 1913 cm^{-}	1949, 1882 cm^{-}

Three J. Young tubes were charged according to Table S2. The $^{31}P\{^1H\}$ chemical shifts for **1** and **16** in C_6D_6 were observed to be 61.0 ppm and 54.7 ppm, respectively. A $^{31}P\{^1H\}$ NMR analysis of a sample of **1** (0.020 mmol, 0.10 M in C_6D_6, 0.20 mL), pyridine (0.40 mmol, 32 uL), and C_6D_6 (0.20 mL) was analyzed in 10 °C increments from 10 °C to 70 °C. See Tables S2 and S3, Figure S10. Results from the Van't Hoff analysis were plotted in Figure S11.

Table S2. Composition of J. Young NMR tubes with **1**, pyridine, and C_6D_6.

	0.10 M of **1** in C_6D_6 (mL)	C_6D_6 (mL)	Pyridine (mL)	$^{31}P\{^1H\}$ NMR Shift	Final Pyridine Conc. (M)
J. Young Tube 1	0.20	0.20	0.20	55.2	4.1 (125 equiv)
J. Young Tube 2	0.20	0.10	0.30	54.7	6.2 (187 equiv)
J. Young Tube 3	0.20	0	0.40	54.7	8.3 (250 equiv)

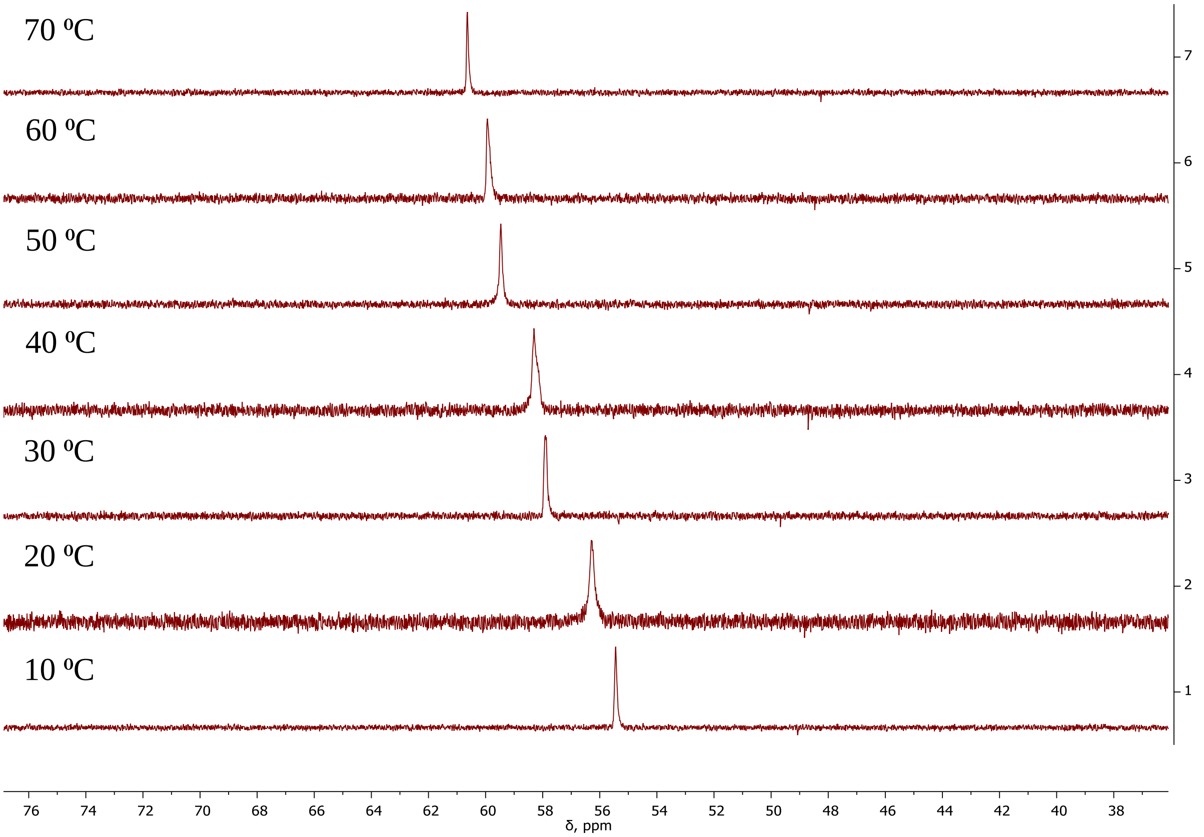

Figure S10. $^{31}P\{^1H\}$ NMR (202 MHz, C_6D_6) analysis of a solution of **1** and pyridine. Sample was analyzed in 10 °C increments from 10 °C to 70 °C.

Table S3. $^{31}P\{^1H\}$ chemical shifts of **1** in the presence pyridine. Sample was analyzed in 10 °C increments from 10 °C to 70 °C.

Temperature (°C)	$^{31}P\{^1H\}$ Chemical Shift (ppm)
70	60.4
60	59.9
50	59.2
40	58.4
30	57.5
20	56.3
10	55.6

The concentration of **1**, pyridine, and **16** were calculated using the following equations. K_{eq} values at each temperature were calculated based on these concentrations.

$$0.020\ \text{mmol}/0.43\ \text{ml} = C_{total}$$

$$\eta_{\mathbf{1}}\delta_{\mathbf{1}} + (1-\eta_{\mathbf{16}})\delta_{\mathbf{1}} = \delta_{mix}$$

$$C_{\mathbf{1}} = \eta_{\mathbf{1}}C_{total}$$

$$C_{py} = (0.40\ \text{mmol}/0.43\ \text{ml}) - C_{\mathbf{16}}$$

$$C_{\mathbf{16}} = \eta_{\mathbf{16}}C_{tot}$$

$$C_{total} = [\mathbf{16}] + [\mathbf{1}]$$

C = concentration

η = mole fraction

δ = $^{31}P\{^1H\}$ chemical shift in ppm

$$K_{eq} = \frac{[\mathbf{1}]}{[\mathbf{16}][\text{Pyridine}]}$$

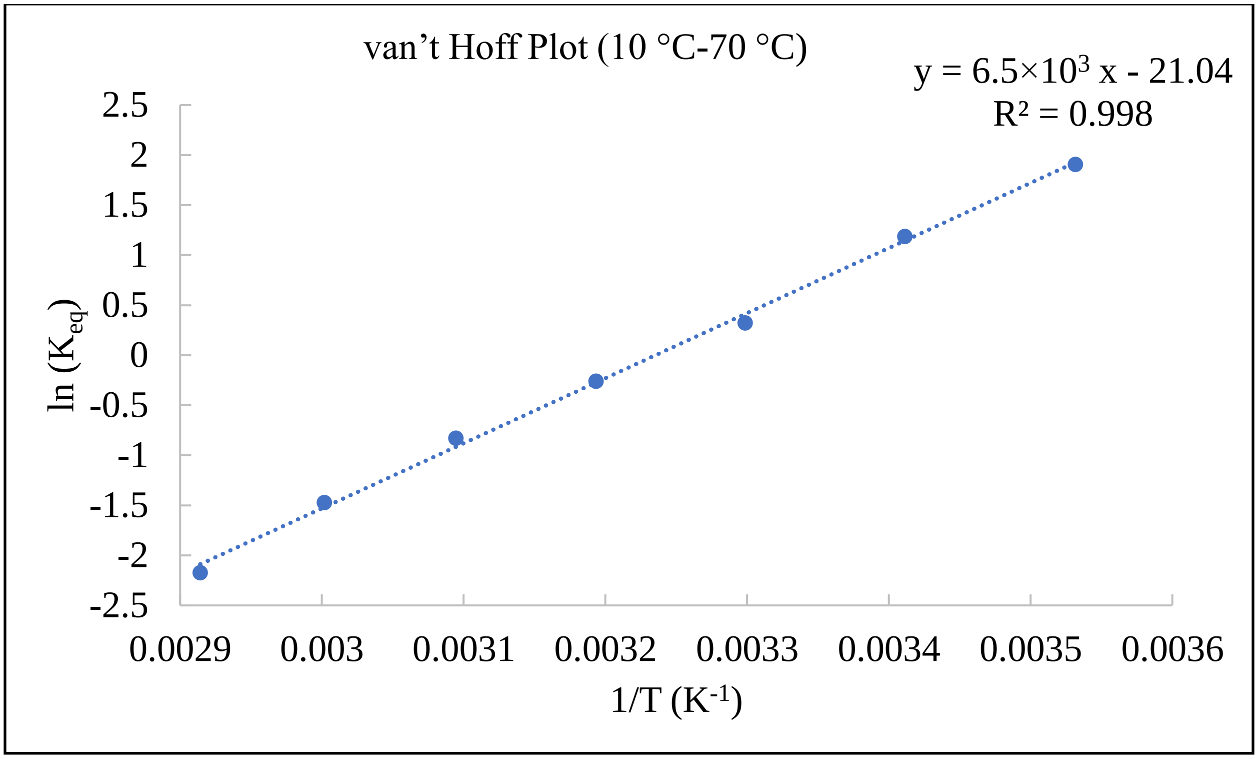

Figure S11. van't Hoff plot with 10 ºC increments from 10-70 °C.

Plotting $\ln(K_{eq})$ vs 1/T using the van't Hoff equation, listed below, yielded a linear relationship.

$$\ln(K_{eq}) = \left(\frac{-\Delta H}{R}\right)\left(\frac{1}{T}\right) + \left(\frac{\Delta S}{R}\right)$$

The slope and Y-intercept values obtained were utilized to extract ΔH and ΔS values, respectively. The following values were obtained: ΔH = -12.9 ± 0.6 kcal/mol and ΔS = -41.8 ± 1.8 cal/mol·K. Errors for ΔH and ΔS were defined as double the standard deviation of the slope and y-intercept provided by the LINEST function in Microsoft Excel, a factor of R (8.3145 J/mol·K), and conversion to kcal/mol and cal/mol·K, respectively.

6.2. Experiments Analyzing the Thermolysis of 3.

6.2.1. Rate Law Study

Four J. Young NMR tubes were charged with 500 μL C_7D_8 solutions containing **3** (40 mM), n-butyl ether (20 mM), and four different concentrations of pyridine (400 mM, 800 mM, 1200 mM, and 1600 mM). Samples were inserted into an NMR probe preheated to 90 °C. Temperatures inside the J. Young NMR tubes were estimated before and after analysis of each sample using neat ethylene glycol as a chemical shift thermometer.[10] Before and after temperatures for all samples varied by less than 1 ºC and reported temperatures are an average of both values. The thermolysis of **3** was monitored by ^{1}H NMR spectroscopy at regular intervals for 80 min (approximately three half-lives). Representative ^{1}H NMR spectra shown in Figures S12 and S13. Thermolysis was assessed by integration of **3**'s C*H*Me_2 multiplet peak at 2.55 ppm. The n-butyl ether signal (triplet at 3.27 ppm) was used as an internal standard. Rate constants at varying pyridine concentrations displayed in Table S4.

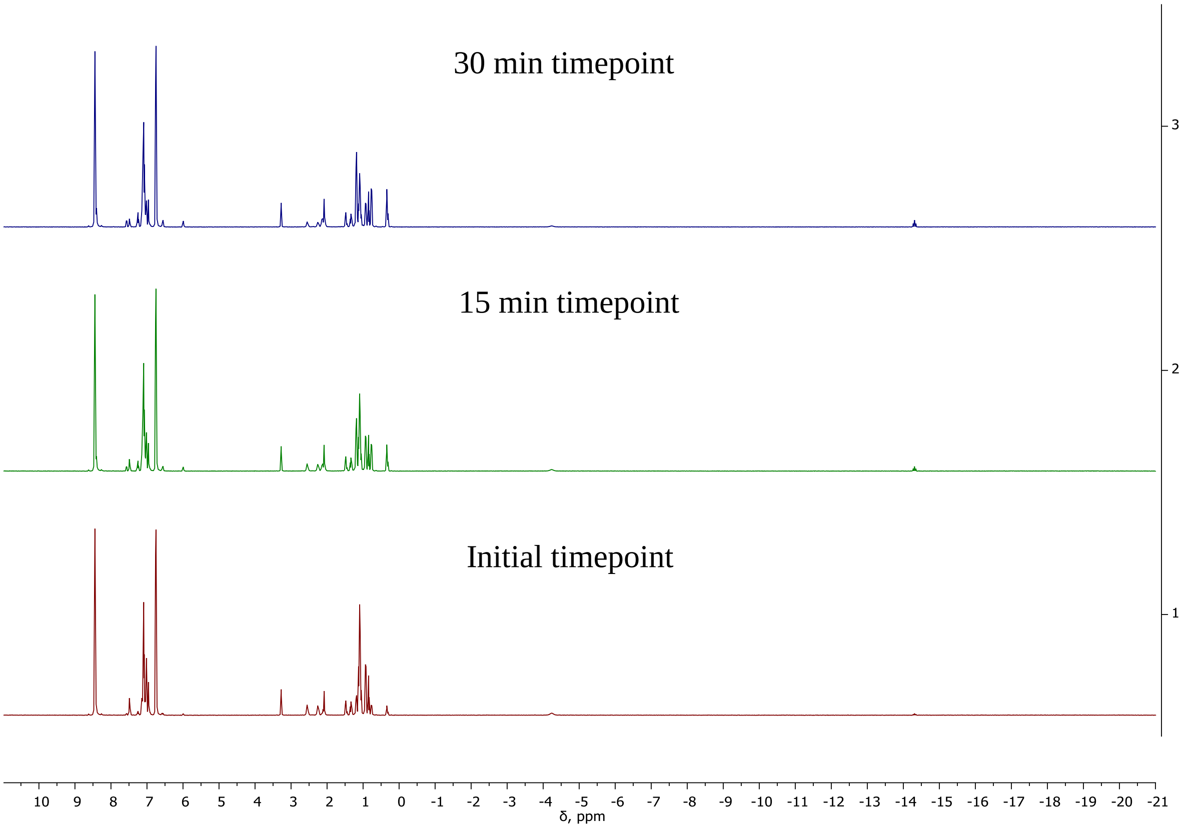

Figure S12. Representative ^{1}H NMR (500 MHz, C_7D_8) spectra from selected datapoints for the thermolysis of **3** at 90 ºC.

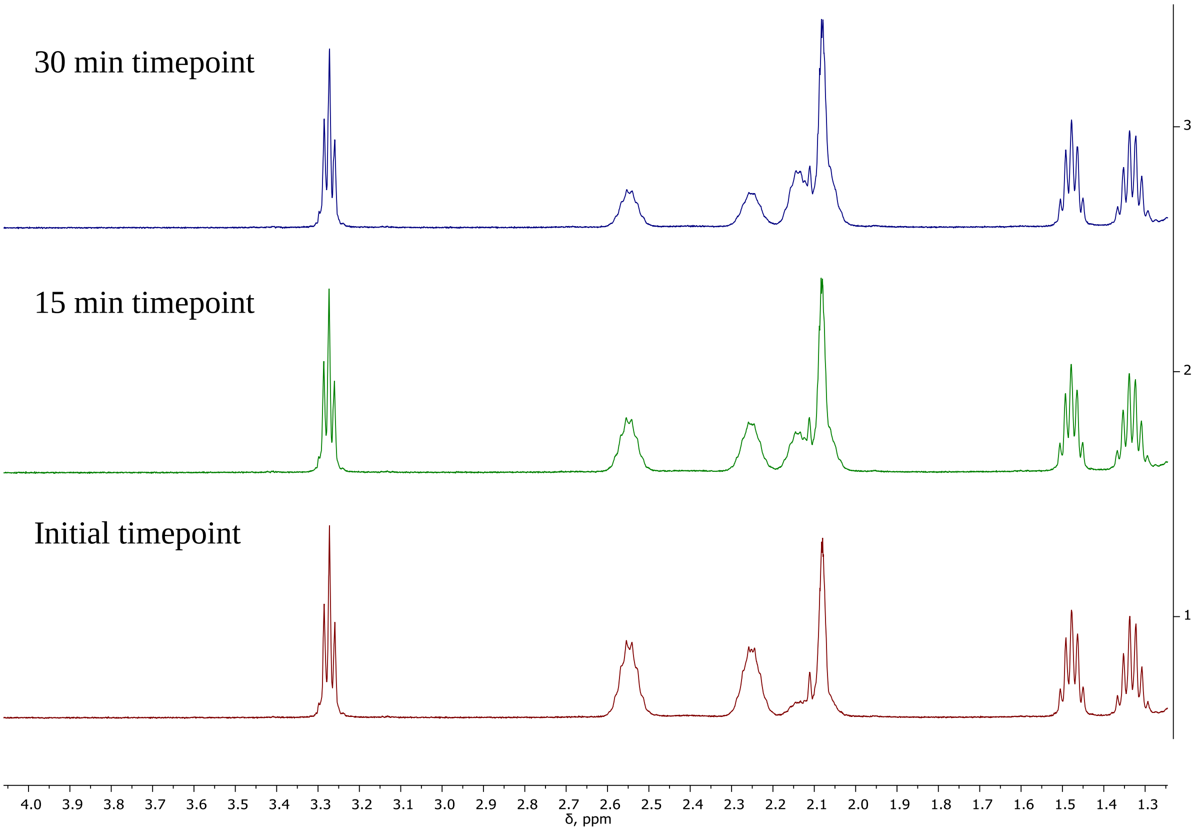

Figure S13. Expanded representative ^{1}H NMR (500 MHz, C_7D_8) spectra for the thermolysis of **3** at 90 ºC.

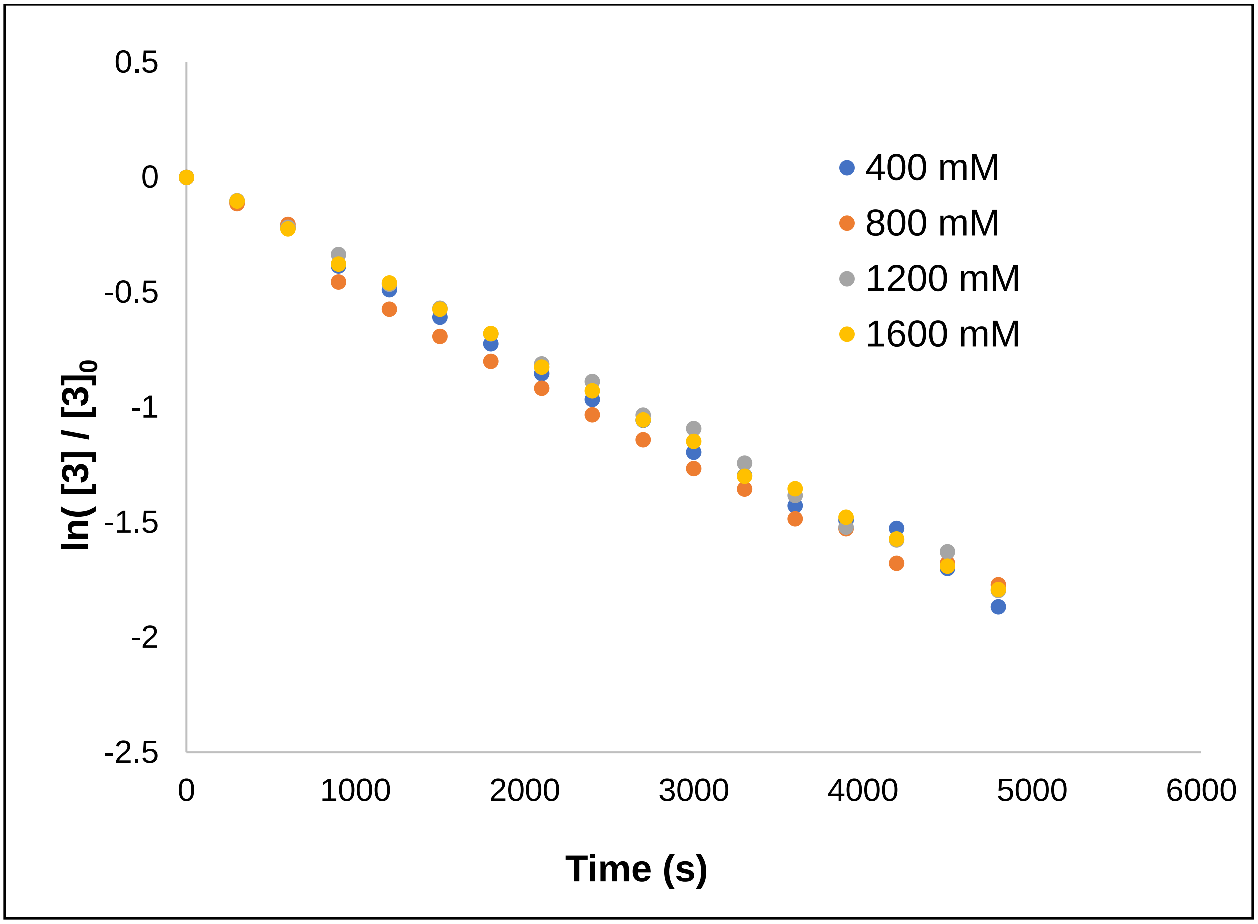

Figure S14. Plots of first order decay of compound **3** at 90 °C in the presence various concentrations of pyridine.

Table S4. Rate constants for the thermolysis of **3** at 90 °C in the presence of four different concentrations of pyridine: 400 mM (10 equiv), 800 mM (20 equiv), 1200 mM (30 equiv), and 1600 mM (40 equiv) in C_7D_8.

**Pyridine Concentration (mM)**	**k (×10^4 sec$^-$)**	**Temperature (^{o}C)**
400	3.8(2)	90.4(10)
800	3.8(3)	90.5(10)
1200	3.8(1)	90.5(10)
1600	3.8(1)	90.5(10)

6.2.2. KIE Studies in the Thermolysis of 3.

A J. Young NMR tube was charged with a 500 µL C_7D_8 solution containing of **3** (40 mM), n-butyl ether (20 mM), and pyridine-d_5 (400 mM). The sample was inserted into an NMR probe, which was preheated at 80 °C. Temperatures inside the J. Young NMR tubes were estimated before and after analysis of each sample using neat ethylene glycol as a chemical shift thermometer.[10] The average of these two measured values was 80.1(1) °C. The thermolysis of **3** was monitored by ^{1}H NMR spectroscopy at regular intervals for 4 h. The rate constant was determined to be 1.27(2) × 10^{-4} sec^{-}. The rate constant for the *protio* equivalent reaction was 1.25(2) x10^{-4} sec^{-}, giving a KIE = 1.02(3).

6.2.3. Eyring Study for the Thermolysis of 3.

Five J. Young NMR tubes were charged with 500 μL C_7D_8 solutions containing **3** (40 mM), n-butyl ether (20 mM), and pyridine (400 mM). Samples were inserted into NMR probes preheated at one of five different temperatures: 60 °C, 70 °C, 80 °C, 90 °C, and 100 °C. Temperatures inside the J. Young NMR tubes were estimated before and after analysis of each sample using neat ethylene glycol as a chemical shift thermometer.[10] Before and after temperatures for all samples varied by less than 1 ºC and reported temperatures are an average of both values. The thermolysis **3** was monitored by ^{1}H NMR spectroscopy at regular intervals for three half-lives. Rate constants and Eyring plot displayed in Table S5.

Table S5. Rate constants for the thermolysis of **3** with 10 equiv (400 mM) pyridine at five different temperatures within a range of 60-100 °C in C_7D_8.

**T (°C)**	**T (K)**	**k (×10^5 sec^{-1})**	**ln (k/T)**	**$t_{1/2}$**
59.9(10)	333.0(10)	1.37(2)	-17.01(1)	14.1(4) h
70.9(10)	344.0(10)	4.81(4)	-15.78(1)	4.00(6) h
80.1(10)	353.3(10)	12.5(2)	-14.85(1)	92(4) min
90.4(10)	363.5(10)	38(2)	-13.77(3)	30(2) min
100.5(10)	373.6(10)	108(3)	-12.75(3)	10.7(4) min

Plotting ln(k/T) vs 1/T using the Eyring equation, listed below, yielded a linear relationship.

$$\ln\left(\frac{k}{T}\right) = \left(\frac{-\Delta H^{\ddagger}}{R}\right)\left(\frac{1}{T}\right) + \ln\left(\frac{k_B}{h}\right) + \left(\frac{\Delta S^{\ddagger}}{R}\right)$$

The slope (-1.30x10^4 K) and Y-intercept (22.05) values obtained were utilized to extract $\Delta H^{\ddagger}$ and $\Delta S^{\ddagger}$ values, respectively. The following values were obtained: $\Delta H^{\ddagger}$ = 25.9 ± 1.0 kcal/mol and $\Delta S^{\ddagger}$

= -3.4 ± 2.9 cal/mol·K. Errors for ΔH‡ and ΔS‡ were determined using error propagation formulas presented by Girolami.[11] The uncertainty in temperature measurements was estimated to be 1 °C or 1 K.

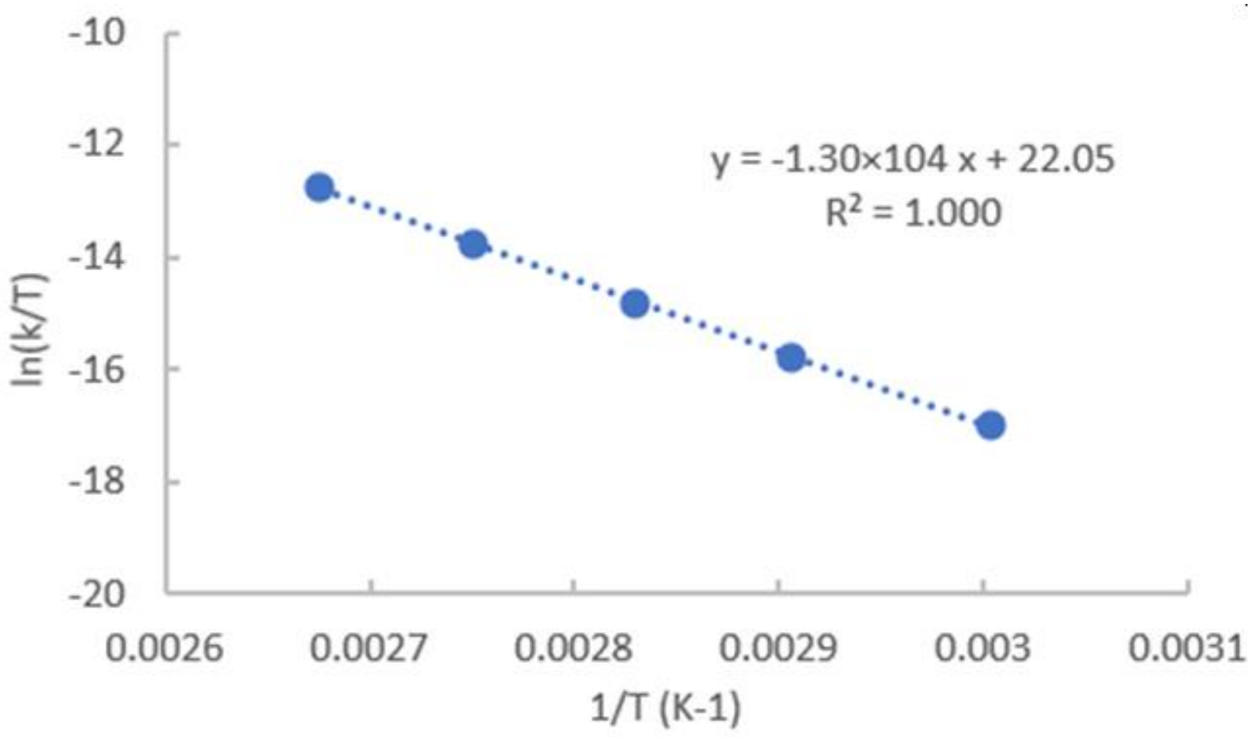

Figure S15. Eyring plot from the thermolysis of compound **3**.

6.3. Experiments Analyzing the Thermolysis of 5.

Two J. Young NMR tubes were charged with 500 µL C_7D_8 solutions containing **5** (50 mM) and n-butyl ether (100 mM). One sample was freeze-pump-thawed and backfilled with one atmosphere of CO gas. The second sample remained under an atmosphere of argon gas. Samples were heated in an oil bath at 105 °C. The thermolysis of **5** was monitored by ^{1}H NMR spectroscopy at 30 min intervals for 2 h. Thermolysis was assessed by integration of **5**'s Ir-*H* ^{1}H NMR chemical shift at 10.75 ppm in relation to an internal standard (*n*-butyl ether, $^{1}H\{^{31}P\}$ NMR chemical shift at 3.27 ppm). Representative spectra displayed as Figures S16 and S17. Plot of the first order decay displayed as Figure S18 and rate constants displayed in Table S6.

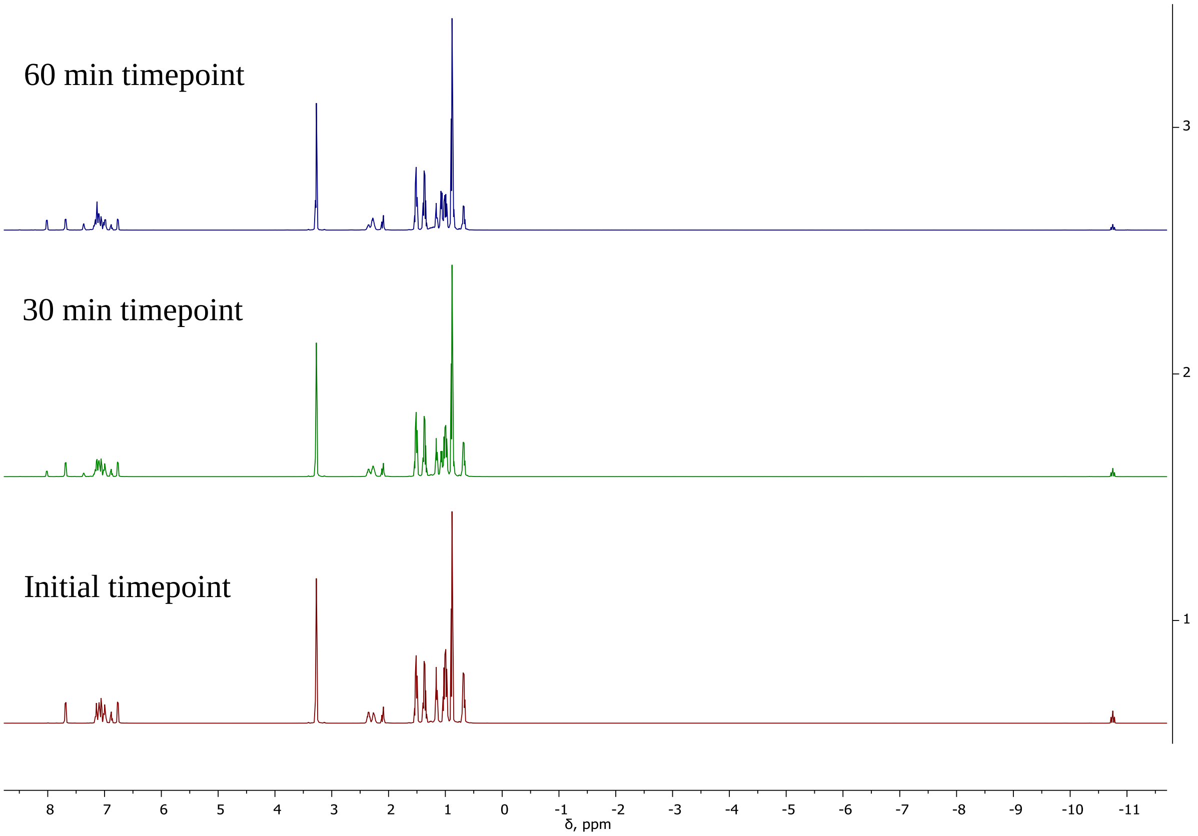

Figure S16. Representative ^{1}H NMR (500 MHz, C_7D_8) spectra for the thermolysis of **5** under an atmosphere of argon gas at 105 ºC over time.

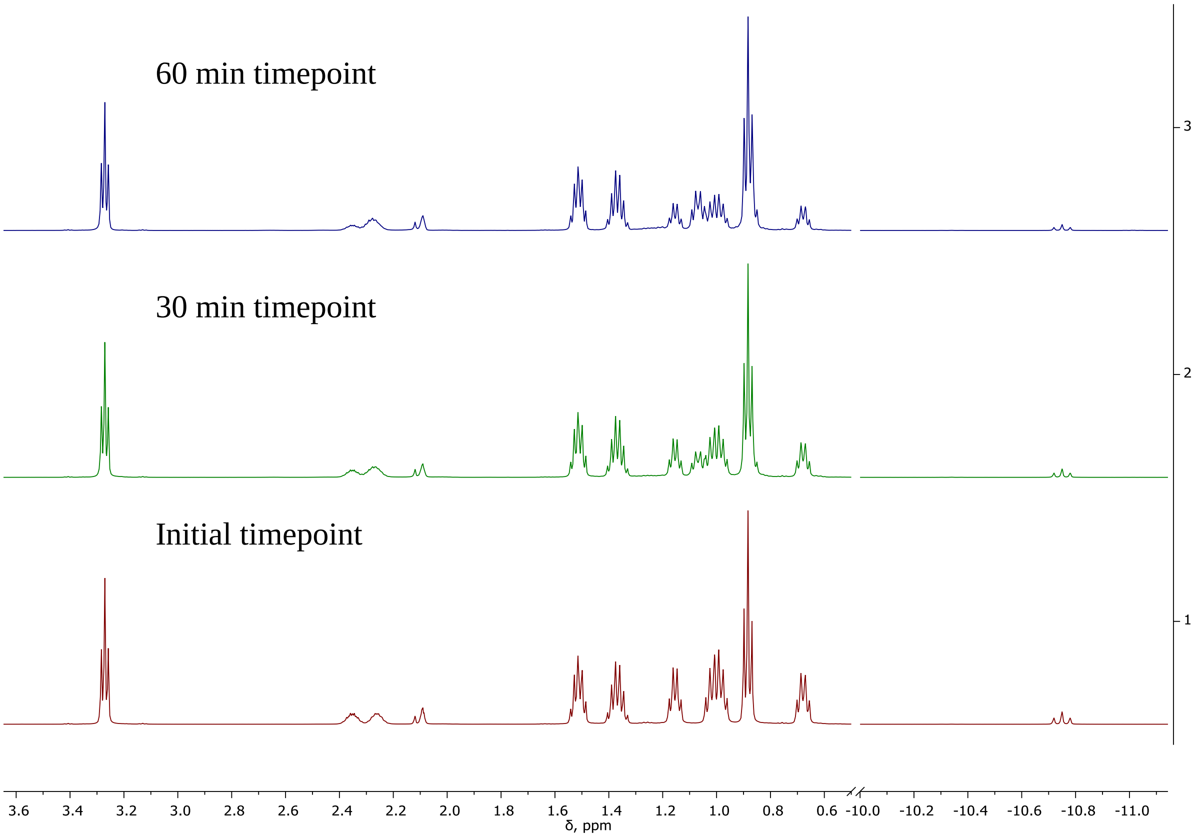

Figure S17. Expanded representative ^{1}H NMR (500 MHz, C_7D_8) spectra for the thermolysis of **5** under an atmosphere of argon gas at 105 ºC over time.

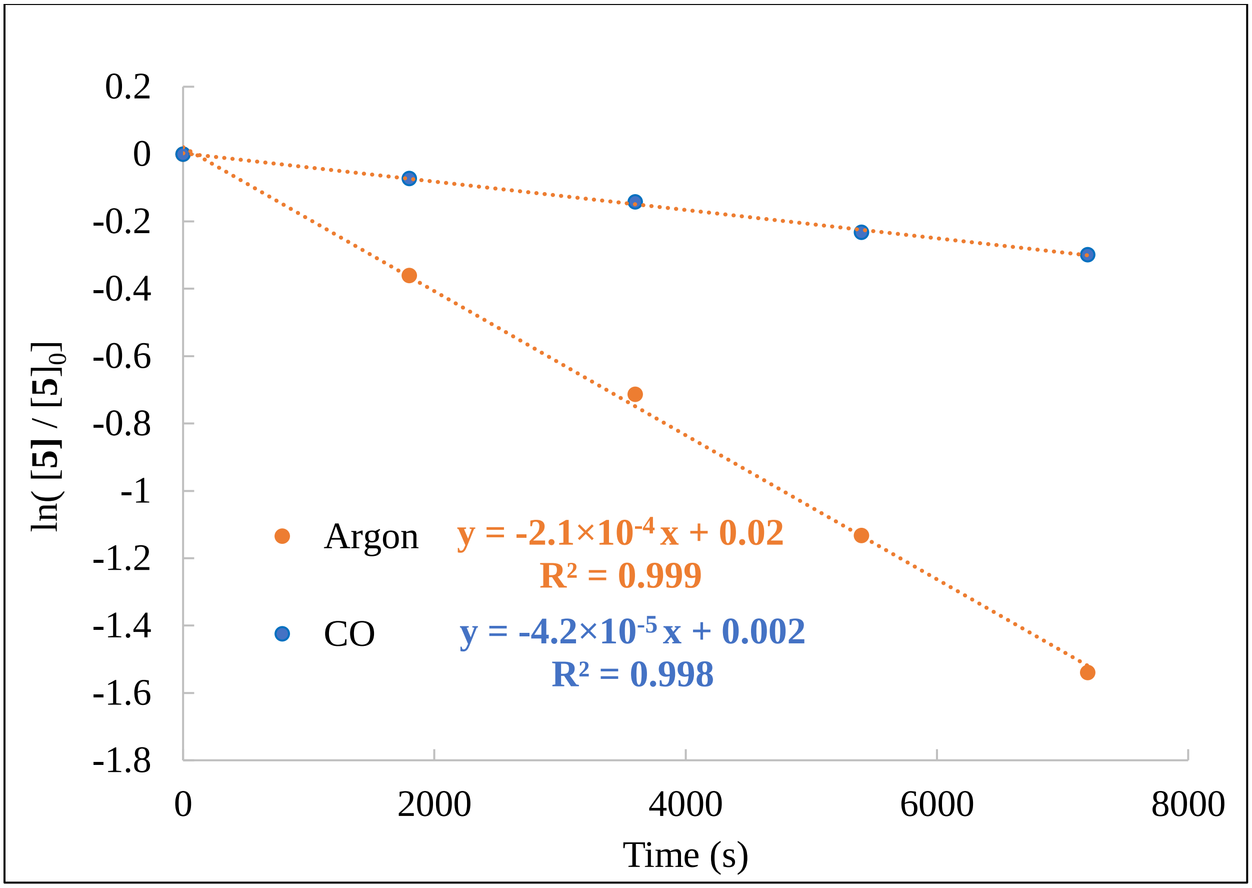

Figure S18. Plot of ln[**5**]/[**5**]$_0$ vs time (s) for the thermolysis of **9** at 105 °C under either an atmosphere of argon or CO.

Table S6. Rate constants for the thermolysis of **5** at 105 °C under an atmosphere of argon or CO.

Atmosphere	k ($\times 10^5$ sec^{-1})
Argon	21.4(10)
CO	4.2(3)

6.4. Experiments Examining the Product Determining Step

6.4.1. Equilibrium Study.

For Entries 1-4 (See Table S7), each entry was prepared using two J. Young NMR tubes. One J. Young NMR tube was charged with a 500 µL C_7D_8 solution containing **2** (40 mM), nine equiv pyridine (360 mM), n-butyl ether (20 mM), and ten equiv (400 mM) of one of the following pyridine derivatives: 4-trifluoromethyl pyridine, dimethylamino pyridine (DMAP), 2-methyl pyridine, pyridine-d_5, or 3-fluoropyridine. The second J. Young NMR tube was charged with a 500 µL C_7D_8 solution containing a (PBP)Ir complex (**2a-2e**) (40 mM), nine equiv (360 mM) of one of the aforementioned pyridines, ten equiv pyridine (400 mM), and n-butyl ether (20 mM). Both samples were heated at 80 °C in an oil bath and analyzed via ^{1}H NMR spectroscopy. Duration values listed in Table S7 were the first observed timepoint at which calculated K_{eq} values for both samples were within 0.1 of one another. K_{eq} values for Entry 1-5 were calculated according to Equation 1. Entry 6 was prepared using a single J. Young NMR tube charged with a 500 µL C_7D_8 solution containing **2** (40 mM), ten equiv 3-fluoro pyridine (400 mM), and n-butyl ether (20 mM). The sample was heated at 80 °C in an oil bath and analyzed via ^{1}H NMR spectroscopy. No chemical shift resonances for C-H activation at the 6-position for 3-fluoropyridine were detected.

Table S7. Thermodynamic ratios from equilibrium reactions.

Entry	Compound	Pyridine Derivative	Duration (d)	K_{eq}
1	**2a**	4-trifluoromethyl pyridine	77	0.8(1)
2	**2b**	DMAP	78	2.8(1)
3	**2c**	2-methyl pyridine	161	0.1(1)
4	**2d**	Pyridine-d_5	44	0.4(1)
5	**2e**	3-fluoro pyridine	37	7.6(1)

6.4.2. Competition Study.

Five J. Young NMR tubes were charged with 500 μL C_7D_8 solutions containing **3** (40 mM), n-butyl ether (20 mM), and 400 mM (10 equiv) of pyridine and 400 mM (10 equiv) of one of the following: 4-trifluoromethyl pyridine, dimethylamino pyridine (DMAP), 2-methyl pyridine, pyridine-d_5, or 3-fluoropyridine. A sixth J. Young NMR tube was charged with a 500 μL C_7D_8 solution containing **3** (40 mM) and 400 mM (10 equiv) of 3-fluoropyridine. Samples were inserted into an NMR probe, which was preheated at 80 °C. Temperatures inside the J. Young NMR tubes were estimated before and after analysis of each sample using neat ethylene glycol as a chemical shift thermometer.[10] Before and after temperatures for all samples varied by less than 1 °C and reported temperatures are an average of both values. The thermolysis **3** was monitored by ^{1}H NMR after 80 min. The ratio of products was determined according to Equation 1 and listed below in Table S8.

Equation 1:

$$\text{Ratio of Products} = \frac{[\mathbf{2}]}{[\mathbf{2x}]}, x = a, b, c, d, or\ e$$

The ratio between **2** and **2a** was determined by comparing the ^{1}H NMR chemical shift resonances at 5.90 ppm for **2** and 6.07 ppm for **2a**. The ratio between **2** and **2b** was determined by comparing the ^{1}H NMR chemical shift resonances at 0.28 ppm and 0.53 ppm for **2**'s CHMe_2 and **2b**'s CHMe_2, respectively. The ratio between **2** and **2c** was determined by comparing the ^{1}H NMR chemical shift resonances at -14.27 ppm and -13.92 ppm for **2**'s Ir-*H* and **2c**'s Ir-H, respectively. The ratio between **2** and **2e** was determined by comparing the integration of the ^{1}H chemical shift resonances at -14.27 ppm and -14.36 ppm for their respective Ir-H. The ratio between **2** and **2d** was determined

by comparing the integration of the ^{1}H chemical signal resonance of the Ir-*H* at -14.27 ppm to that of the CHMe_2 resonance at 0.27 ppm. Overlapping CHMe_2 signals for **2** and **2d**, but an absence of an Ir-*H* chemical signal for **2d**, allows for the calculation of the ratio. The major product was determined to be >98% **2e** (position 2), with no clear indication that position 6 was activated.

Table S8. Product ratio after thermolysis of **3** with 10 equiv of pyridine and 10 equiv of a pyridine derivative at 80 ºC for 80 min. Values for entries 1-5 are relative to concentration of **2**.

Entry	Compound	Product Ratio
1	**2a**	0.8
2	**2b**	3.5
3	**2c**	0.1>
4	**2d**	0.35
5	**2e**	1.6
6	**2e** (2-position/6-position)	>10

6.5. *In Situ* Analysis of the Thermolysis of 3 in Benzene.

In an Ar-filled glovebox, 8.3 mg (0.01 mmol) of **3** and 600 µL C_6D_6 were introduced into a J. Young NMR tube. After heating at 100 ºC for 2 h in an oil bath, the dark brown reaction mixture was analyzed via ^{1}H, $^{11}B\{^{1}H\}$, and $^{31}P\{^{1}H\}$ NMR spectroscopy. The sample was analyzed via ^{1}H and $^{31}P\{^{1}H\}$ NMR spectroscopy after further thermolysis at the 4 h and 15 h timepoints. Then, 1 µL (0.01 mmol) pyridine was added via microsyringe to the remaining reaction mixture, causing an immediate color change to orange-red. This mixture was analyzed via ^{1}H, $^{11}B\{^{1}H\}$, and $^{31}P\{^{1}H\}$ NMR spectroscopy 10 min after pyridine addition, 3 h after pyridine addition, and after thermolysis at 100 ºC for 3 h. See Figures S19-S22 and Table S9.

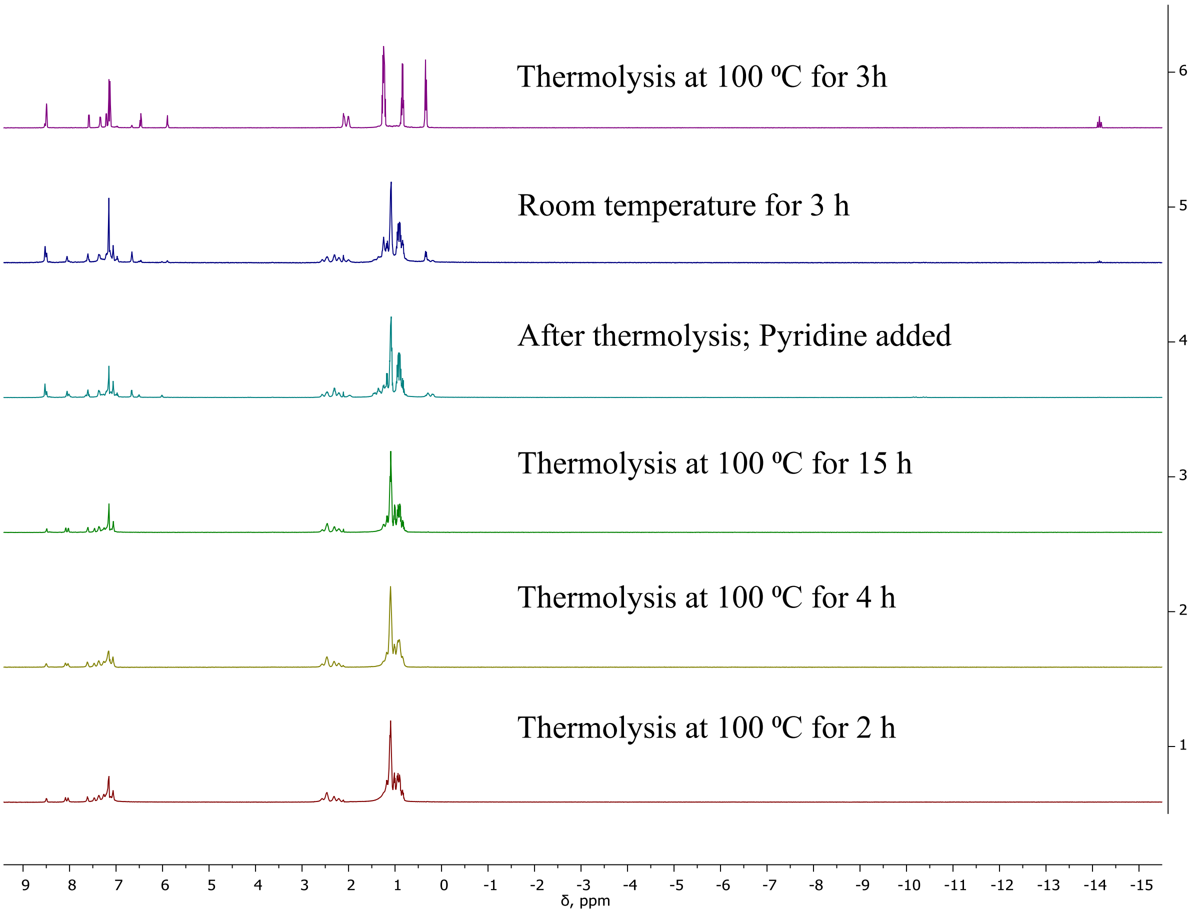

Figure S19. Representative ^{1}H NMR (500 MHz, C_6D_6) spectra for the thermolysis of **3** after (from bottom to top) thermolysis at 100 ºC for 2 h, thermolysis for 4 h, thermolysis for 15 h, addition of pyridine after remaining at room temperature for 3 h, and subsequent thermolysis at 100 ºC for 3 h.

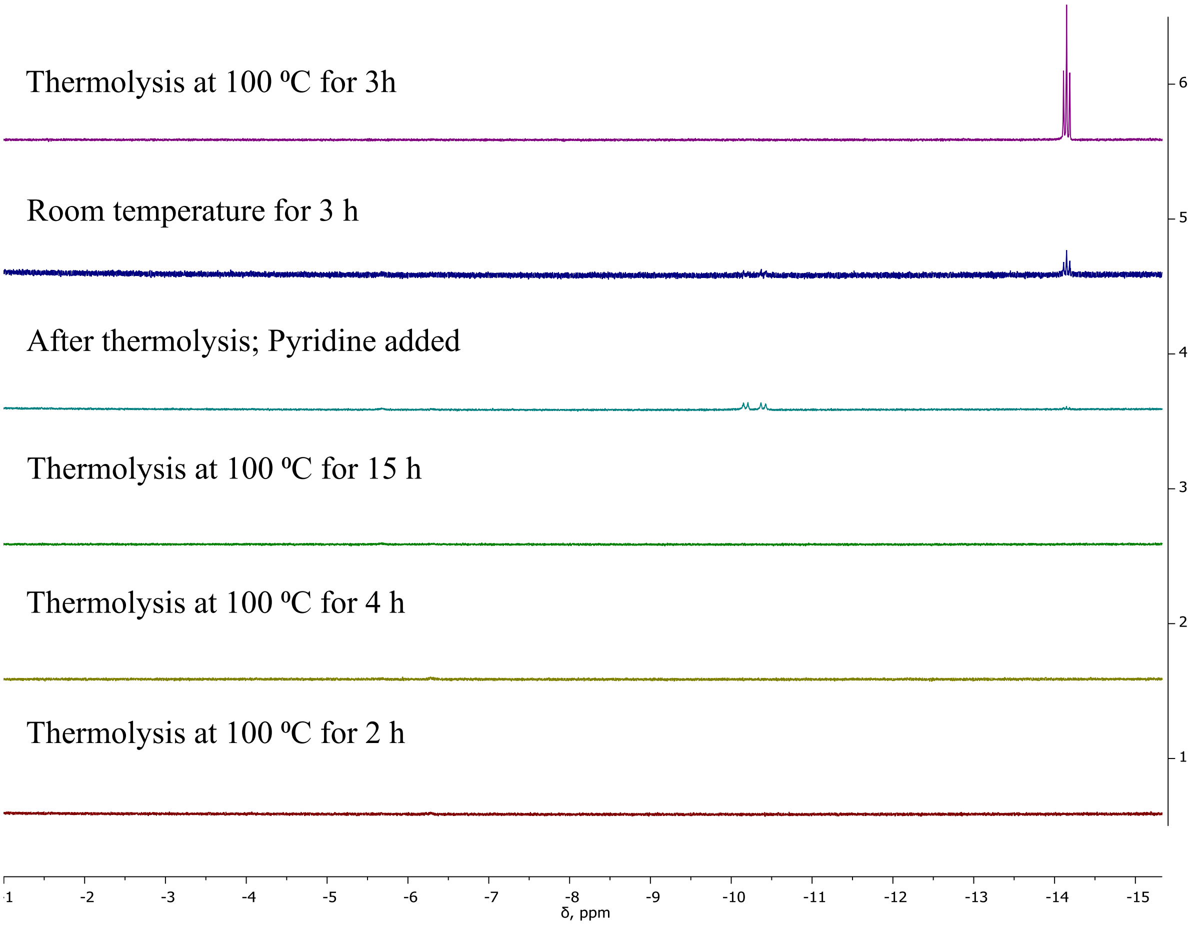

Figure S20. Expanded representative ^{1}H NMR (500 MHz, C_6D_6) spectra for the thermolysis of **3** after (from bottom to top) thermolysis at 100 ºC for 2 h, thermolysis for 4 h, thermolysis for 15 h, addition of pyridine, after remaining at room temperature for 3 h, and subsequent thermolysis at 100 ºC for 3 h.

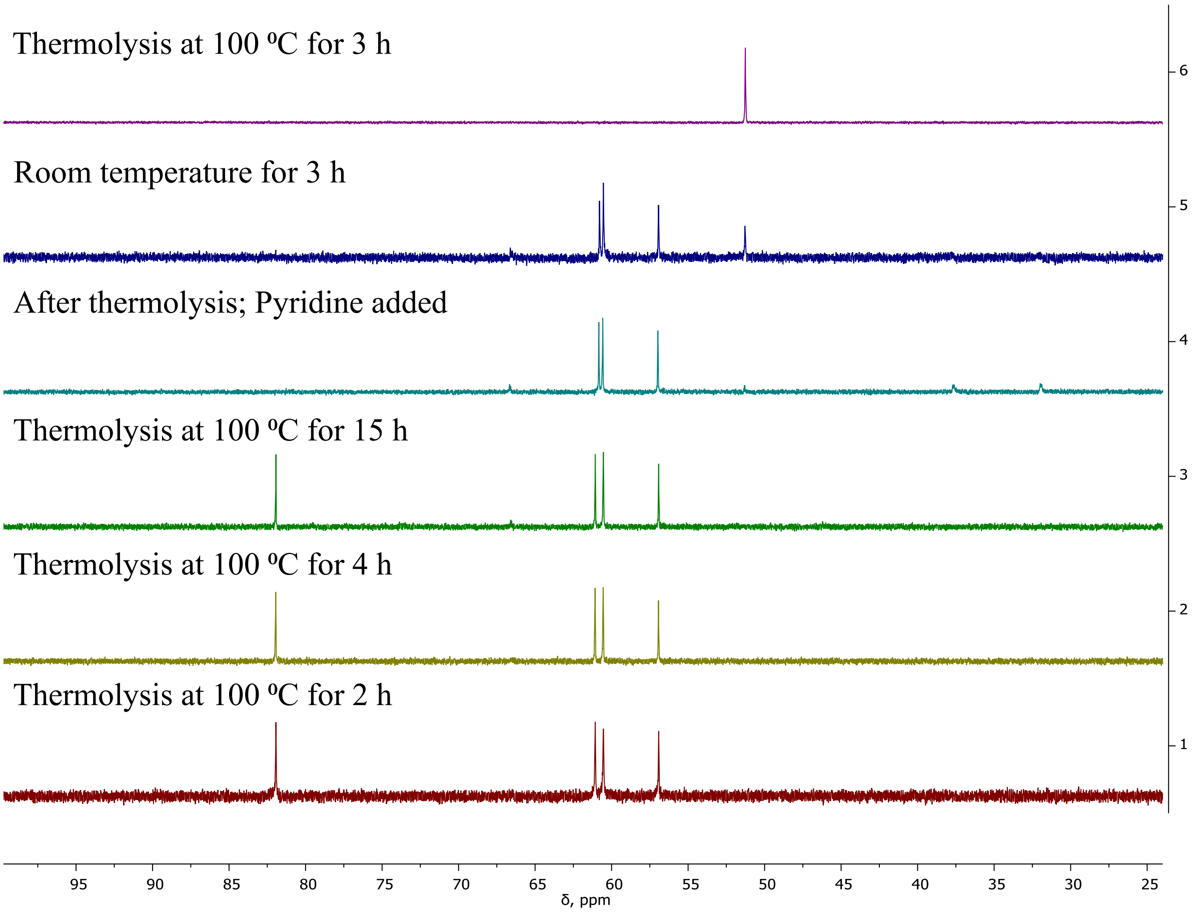

Figure S21. Representative $^{31}P\{^{1}H\}$ NMR (202 MHz, C_6D_6) spectra for the thermolysis of **3** after (from bottom to top) thermolysis at 100 ºC for 2 h, thermolysis for 4 h, thermolysis for 15 h, addition of pyridine, after remaining at room temperature for 3 h, and subsequent thermolysis at 100 ºC for 3 h.

Table S9. Percent composition of each compound based on the $^{31}P\{^1H\}$ NMR spectra of the reaction mixture. Note: a previously identified compound, (PBP)Ir$(H)_2$CO (**9**), appears in trace amounts as an impurity.[1]

**Compound**	**2 h at 100 ºC**	**4 h at 100 ºC**	**15 h at 100 ºC**	**py addition**	**3 h at RT**	**3 h at 100 ºC**
**3**	35%	33%	33%	32%	31%	-
**17**	21%	22%	21%	22%	22%	-
**1 + 16**	21%	22%	23%	22%	22%	-
**4**	23%	23%	23%	-	-	-
**12**	-	-	-	20%	6%	-
**2**	-	-	-	3%	19%	100%

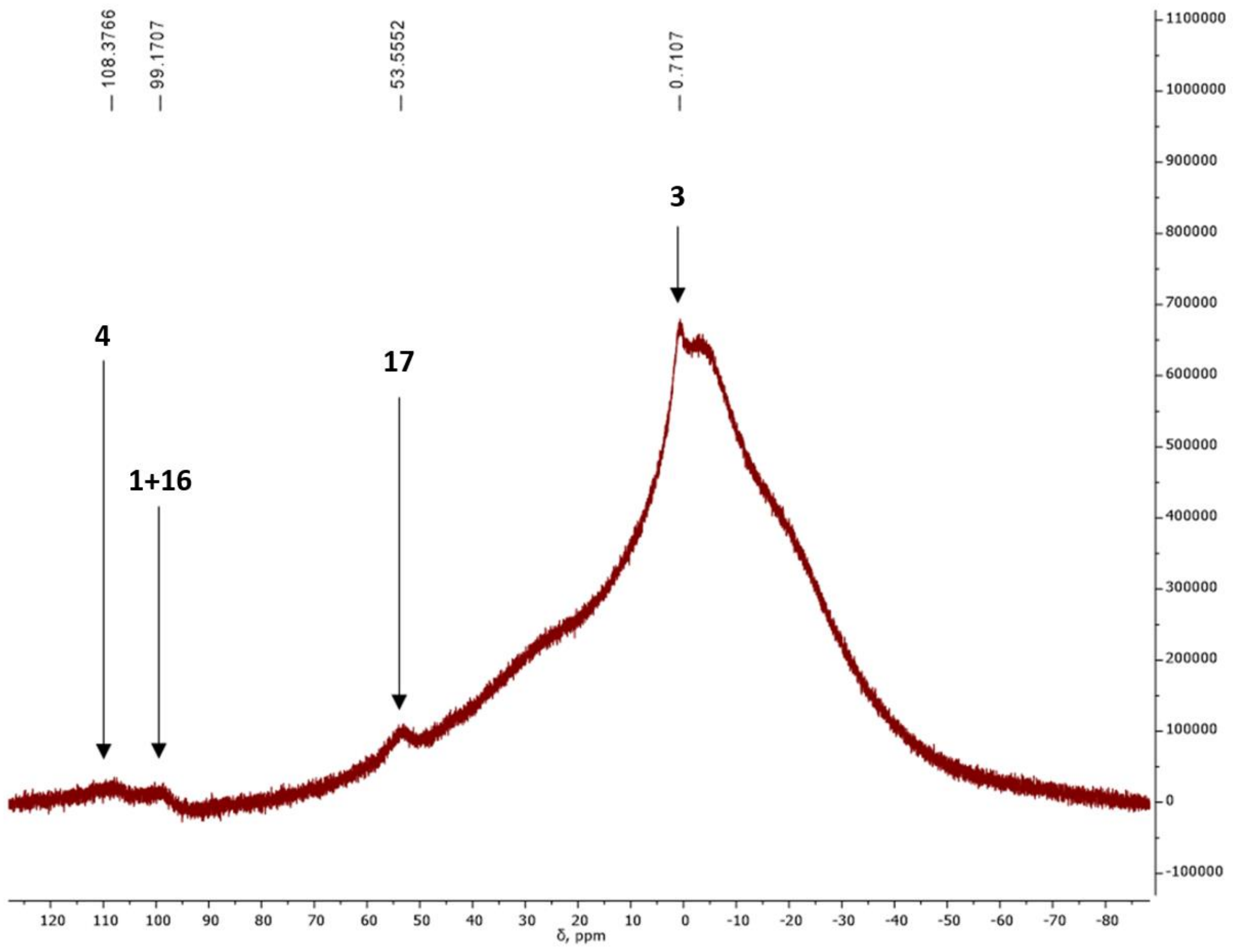

Figure S22. $^{11}B\{^{1}H\}$ NMR (160 MHz, C_6D_6) spectrum of the thermolysis of **3** at 100 ºC for 2 h.

In an Ar-filled glovebox, a 400 µL solution of **3** (40 mM in C_6D_6) was introduced into a J. Young NMR tube. The sample was heated at 100 ºC for 2 h in an oil bath and analyzed via $^{31}P\{^1H\}$ NMR spectroscopy. A 200 µL solution of **1** (40 mM in C_6D_6) was then added via microsyringe and the solution was immediately analyzed via $^{31}P\{^1H\}$ NMR spectroscopy. The sample was then heated at 100 ºC for an additional 2 h in an oil bath and analyzed via $^{31}P\{^1H\}$ NMR spectroscopy. See Figures S23 and S24, and Table S10. The concentration of benzene was calculated from the volume of the solution, excluding the volume of the dissolved solids. K_{eq} values were calculated according to the following equation:

$$K_{eq1} = \frac{[\mathbf{4}][\text{Benzene}]}{[\mathbf{3}]}$$

$$K_{eq2} = \frac{[\mathbf{17}][\mathbf{1}]}{[\mathbf{3}][\mathbf{4}]}$$

$$K_{eq3} = K_{eq1} * K_{eq2} = \frac{[\mathbf{17}][\mathbf{1}][\text{Benzene}]}{[\mathbf{3}]^2}$$

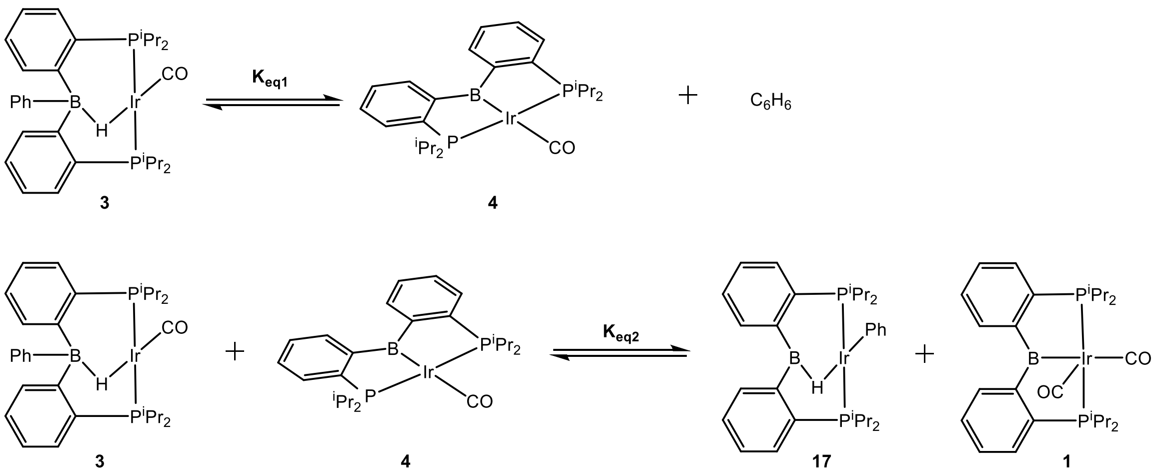

Figure S23. Proposed equilibria established by thermolysis of **3** at 100 ºC.

Table S10. Percent composition of each compound based on the $^{31}P\{^{1}H\}$ NMR spectra of the reaction mixture. The middle column in italic is a mixture not in equilibrium.

**Compound**	**2 h at 100 ºC**	***Compound 1 added***	**2 h at 100 ºC**
**3**	32%	*24%*	27%
**4**	24%	*14%*	20%
**1**	23%	*47%*	45%
**17**	21%	*15%*	8%
$\mathbf{K_{eq1}}$	8.63	*6.55*	8.28
$\mathbf{K_{eq2}}$	0.63	*2.22*	0.65
$\mathbf{K_{eq3}}$	5.43	*14.5*	5.35

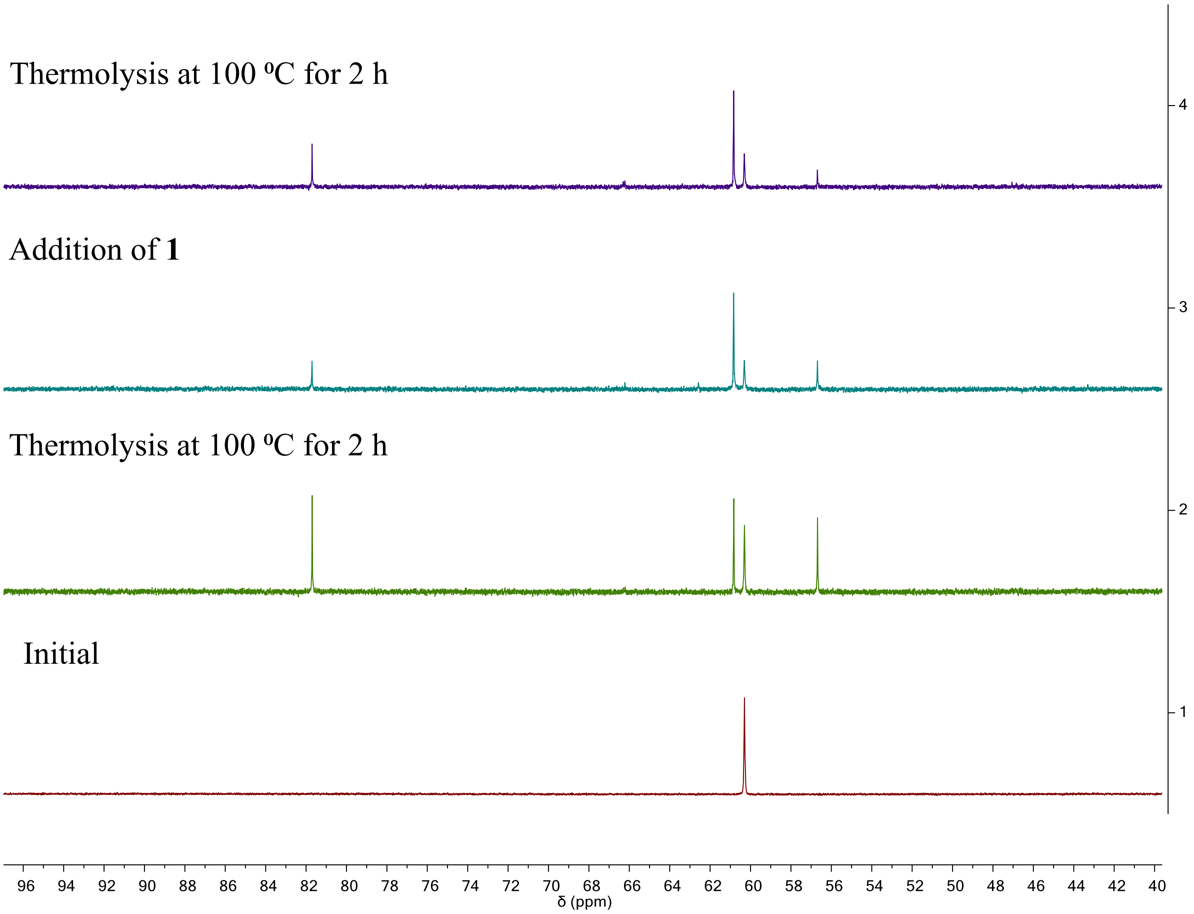

Figure S24. $^{31}P\{^{1}H\}$ NMR (202 MHz, C_6D_6) spectrum of the thermolysis of **3** (from bottom to top) prior to heating, after thermolysis at 100 ºC for 2 h, addition of **1**, and thermolysis at 100 ºC for an additional 2 h.

6.6. Experiments Analyzing the Thermolysis of 2, 2a, and 2d.

6.6.1. Rate Law and KIE study

Two J. Young NMR tubes were charged with 600 µL C_7D_8 solutions containing **2** (20 mM), ten equiv DMAP (200 mM), and n-butyl ether (30 mM). One sample was degassed three times using the freeze-pump-thaw technique and backfilled with one atmosphere of CO gas. The second sample remained under an atmosphere of argon gas. A third J. Young NMR tube was charged with 600 µL C_7D_8 solutions composed of ten equiv DMAP (200 mM), n-butyl ether (30 mM), and **2d** (20 mM). Samples were heated in an oil bath at 110 °C. The thermolysis of **2** and **2d** was monitored by $^{1}H\{^{31}P\}$ NMR spectroscopy. Data points were collected for 17.5 h of heating. Thermolysis was assessed by integration of **2** and **2d**'s CHMe_2 $^{1}H\{^{31}P\}$ NMR chemical shift at 0.27 ppm in relation to an internal standard (n-butyl ether, $^{1}H\{^{31}P\}$ NMR chemical shift at 3.26 ppm). The rate constant for the thermolysis of **2** (under argon) and **2d** was $2.0(2)\times10^{5}$ sec^{-1} and $1.7(1)\times10^{5}$ sec^{-1}, respectively, with a KIE value of 1.2(1). See Figure S25 and S26 for representative spectra. See Figure S27 for the plot of the first-order decay and Table S11 for tabulated rate constants.

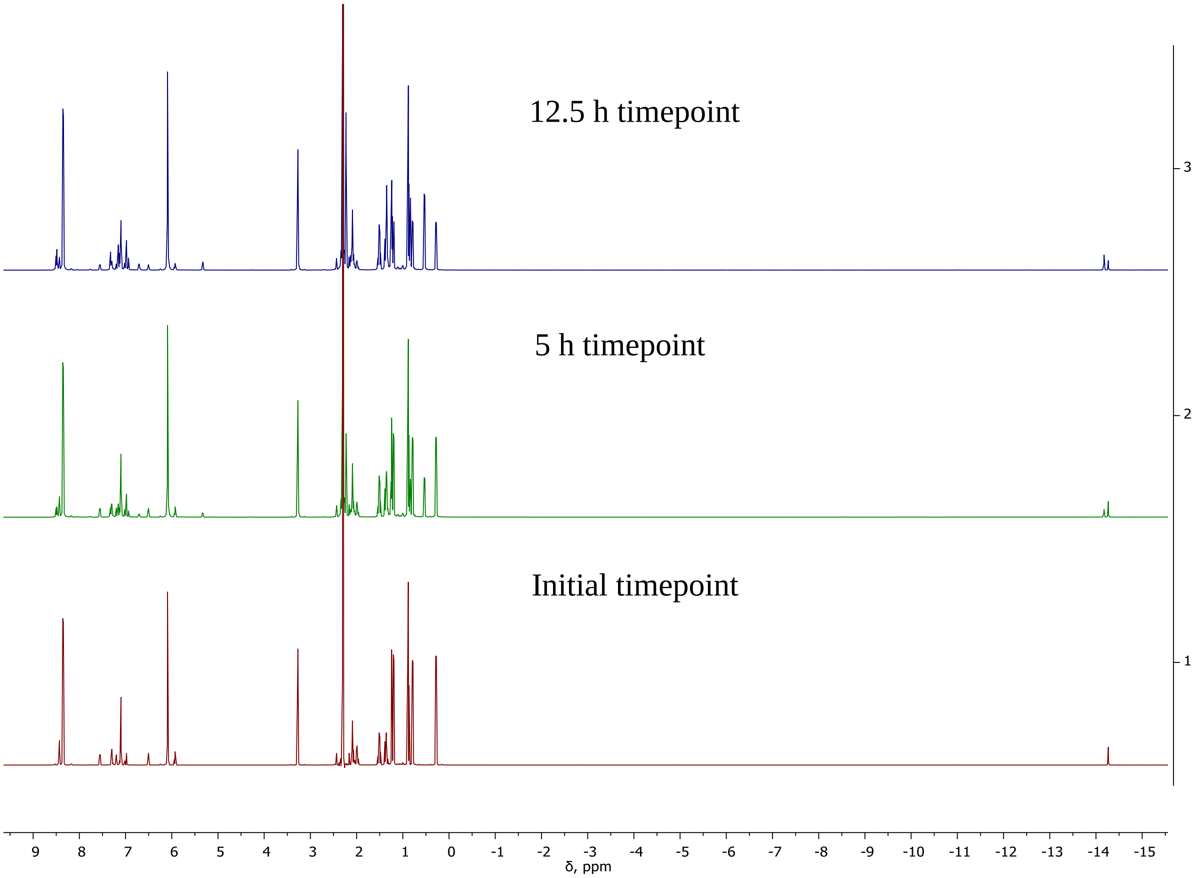

Figure S25. Representative $^{1}H\{^{31}P\}$ NMR (500 MHz, C_7D_8) spectra for the elimination of pyridine from compound **2** at 110 ºC.

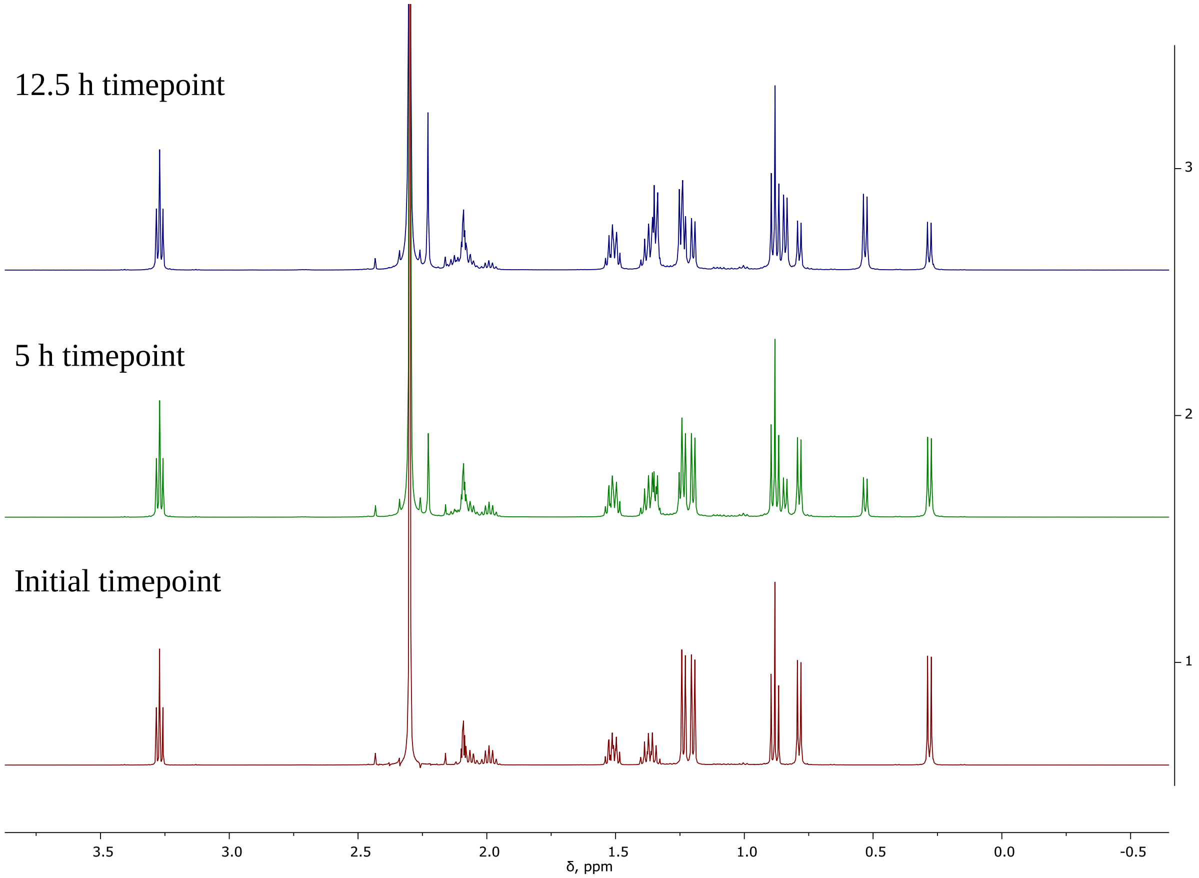

Figure S26. Expanded representative $^{1}H\{^{31}P\}$ NMR (500 MHz, C_7D_8) spectra for the elimination of pyridine from **2** at 110 ºC.

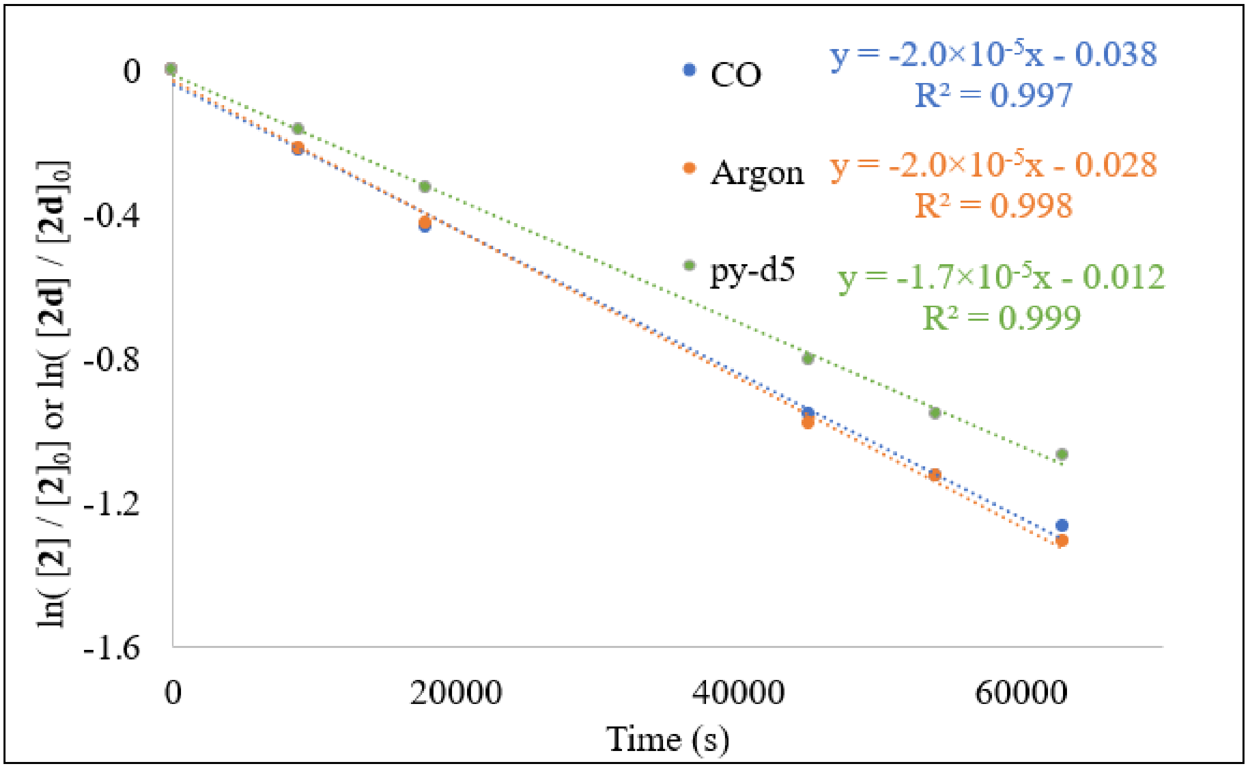

Figure S27. Plot of ln[**2**]/[**2**]$_0$ or ln[**2d**]/[**2d**]$_0$ vs time (s) for the thermolysis of **2** under an atmosphere of argon or carbon monoxide, and **2d** under an atmosphere of argon with ten equiv DMAP at 110 °C.

Table S11. Rate constants for the thermolysis of **2** or **2d** in the presence of ten equiv DMAP at 110 °C under an atmosphere of argon or carbon monoxide.

Condition	k ($\times 10^5$ sec^{-1})
Argon	2.0(2)
CO	2.0(2)
Argon, py-d_5	1.7(1)

6.6.2. Eyring Study of the Thermolysis of 2a with DMAP.

Five J. Young NMR tubes were charged with 600 μL mesitylene solutions containing **2a** (20 mM), ten equiv DMAP (200 mM), and benzotrifluoride (40 mM). Samples were heated in an oil bath at one of five different temperatures: 110 °C, 120 °C, 130 °C, 140 °C, and 150 °C. Thermolysis of **2a** was monitored by ^{19}F NMR at regular intervals for two half-lives (see Figure S28). Rate constants and the Eyring plot are displayed in Table S12 and Figure S29. Thermolysis was assessed by integration of **2a**'s CF_3 ^{19}F NMR chemical shift at -66.3 ppm in relation to an internal standard (benzotrifluoride, ^{19}F NMR chemical shift at -63.7 ppm). The emerging ^{19}F NMR chemical shift at -66.1 ppm belongs to free 4-trifluoromethyl pyridine.

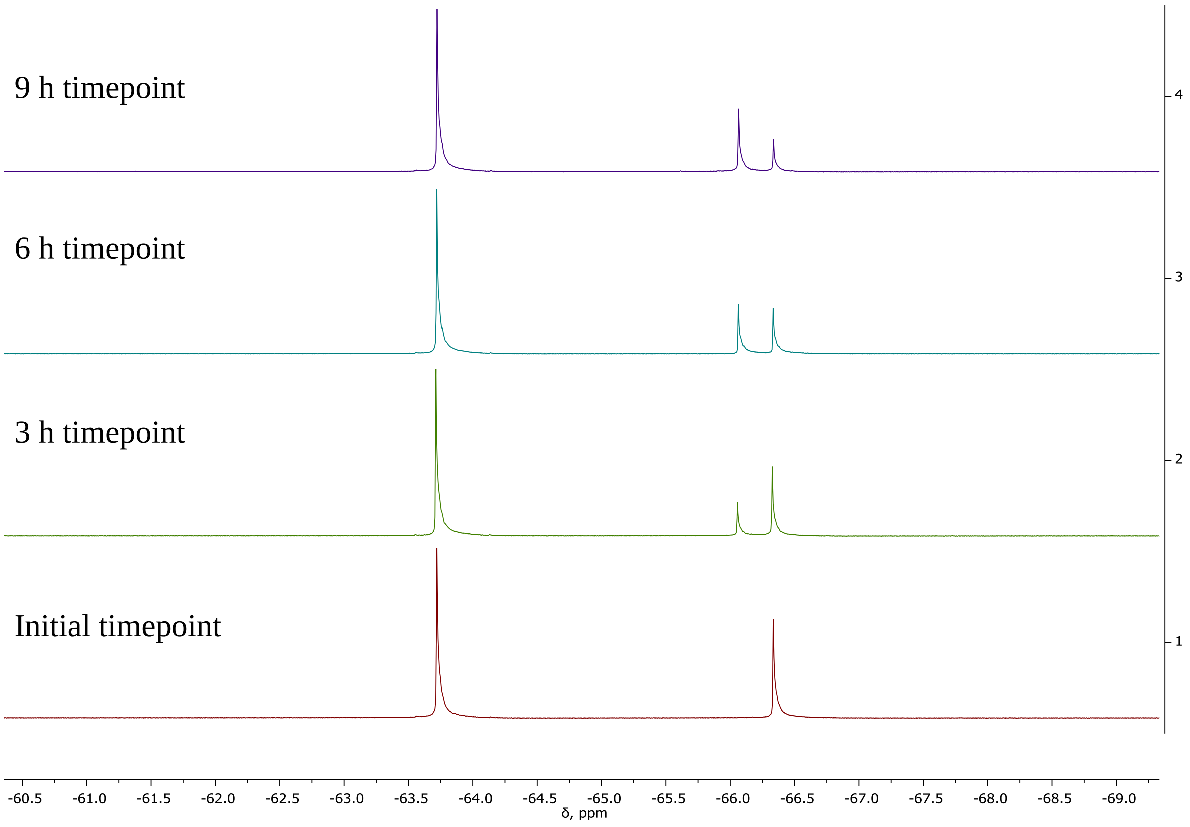

Figure S28. Representative ^{19}F NMR (471 MHz, mesitylene) spectra for the thermolysis of **2a** in the presence of 10 equiv DMAP at 120 ºC over time.

Table S12. Rate constants for the thermolysis of **2a** in the presence of ten equiv DMAP between a temperature range of 110-150 °C for two half-lives.

T (°C)	T (K)	k (×10^5 sec^{-1})	ln (k/T)	$t_{1/2}$
110(1)	383(1)	1.27(3)	-17.22(2)	15.2(4) h
120(1)	393(1)	2.9(2)	-16.42(2)	6.6(3) h
130(1)	403(1)	8.8(3)	-15.34(2)	2.2(1) h
140(1)	413(1)	23.4(7)	-14.38(2)	49(2) min
150(1)	423(1)	47.0(9)	-13.71(1)	24.6(5) min

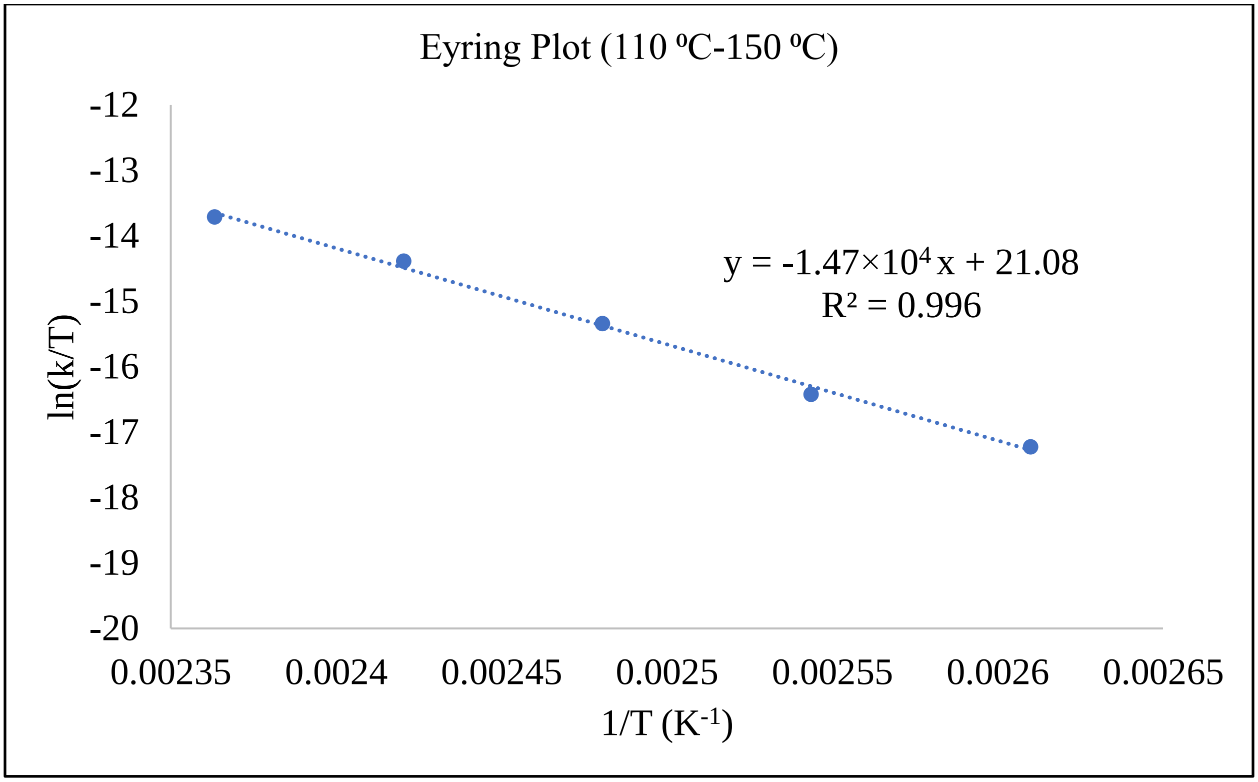

Figure S29. Eyring plot of the thermolysis of **2a** in the presence of ten equiv DMAP at five temperatures between a range of 110-150 °C for two half-lives.

Plotting ln(k/T) vs 1/T using the Eyring equation, listed below, yielded a linear relationship.

$$\ln\left(\frac{k}{T}\right) = \left(\frac{-\Delta H^{\ddagger}}{R}\right)\left(\frac{1}{T}\right) + \ln\left(\frac{k_B}{h}\right) + \left(\frac{\Delta S^{\ddagger}}{R}\right)$$

The slope and Y-intercept values obtained were utilized to extract $\Delta H^{\ddagger}$ and $\Delta S^{\ddagger}$ values, respectively. The following values were obtained: $\Delta H^{\ddagger}$ = 29.2 ± 1.2 kcal/mol and $\Delta S^{\ddagger}$ = -5.3 ± 3.0 cal/mol·K. Errors for $\Delta H^{\ddagger}$ and $\Delta S^{\ddagger}$ were determined using error propagation formulas presented by Girolami.[11] The uncertainty in temperature measurements was estimated to be 1 °C or 1 K.

7. Kinetic Studies with Rh Compounds.

7.1. Rate determination for the thermolysis of 3-Rh.

In a J. Young tube, a 540 µL cyclooctane solution of **3-Rh** (30 mg, 0.049 mmol) containing 60 µL of toluene-d_8 (for locking purposes) and a capillary tube with PPh_3 (50 µL, 0.025 mmol, 0.50 M in cyclooctane) was prepared. The sample was thermolyzed at 150 °C and monitored by $^{31}P\{^1H\}$ NMR spectroscopy in 45 min intervals for 315 min. The reaction was found to be 1st order with respect to **3-Rh** and the rate constant was determined to be 1.5(4) x 10^{-4} sec^{-1}. This is slightly faster than the experiments in Section 7.3, but those were set up separately, and in a different solvent (mesitylene). There is a possibility of a slightly different temperature of the oil bath in separately set up experiments.

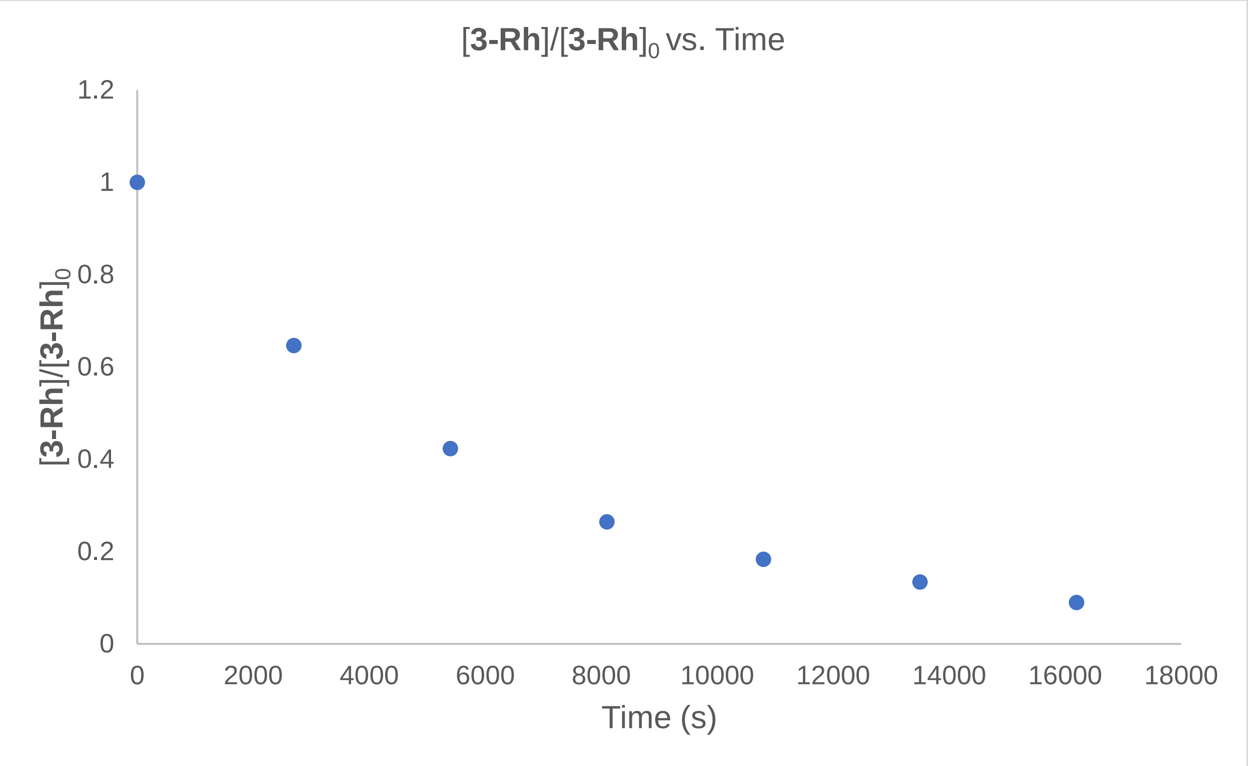

Figure S30. Concentration vs. time plot for the thermolysis of **3-Rh** in cyclooctane.

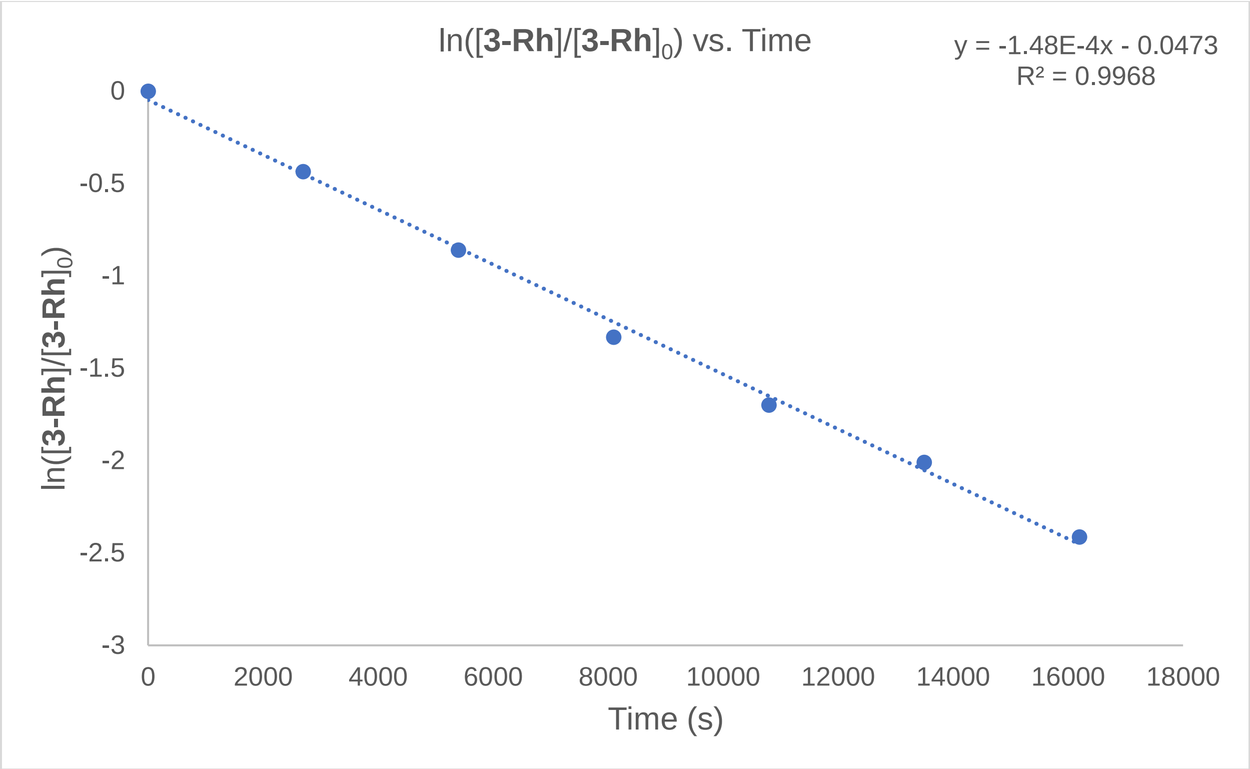

Figure S31. 1st order integrated rate law plot for the thermolysis of **3-Rh** in cyclooctane.

7.2. H/D Exchange Experiments.

Test for H/D exchange for the thermolysis of 3-Rh-d in cyclooctane. In a J. Young tube, a 600 μL cyclooctane solution of **3-Rh-d** (20 mg, 0.033 mmol) was thermolyzed at 150 ˚C for 6 h. The volatiles were vacuum transferred into a separate J. Young tube. ^{2}H NMR analysis revealed the presence of deuterated cyclooctane and benzene (Figure S32). ^{1}H NMR analysis of the non-volatiles revealed hydride signals corresponding to **9-Rh** (Figure S33), indicating H/D exchange with cyclooctane.

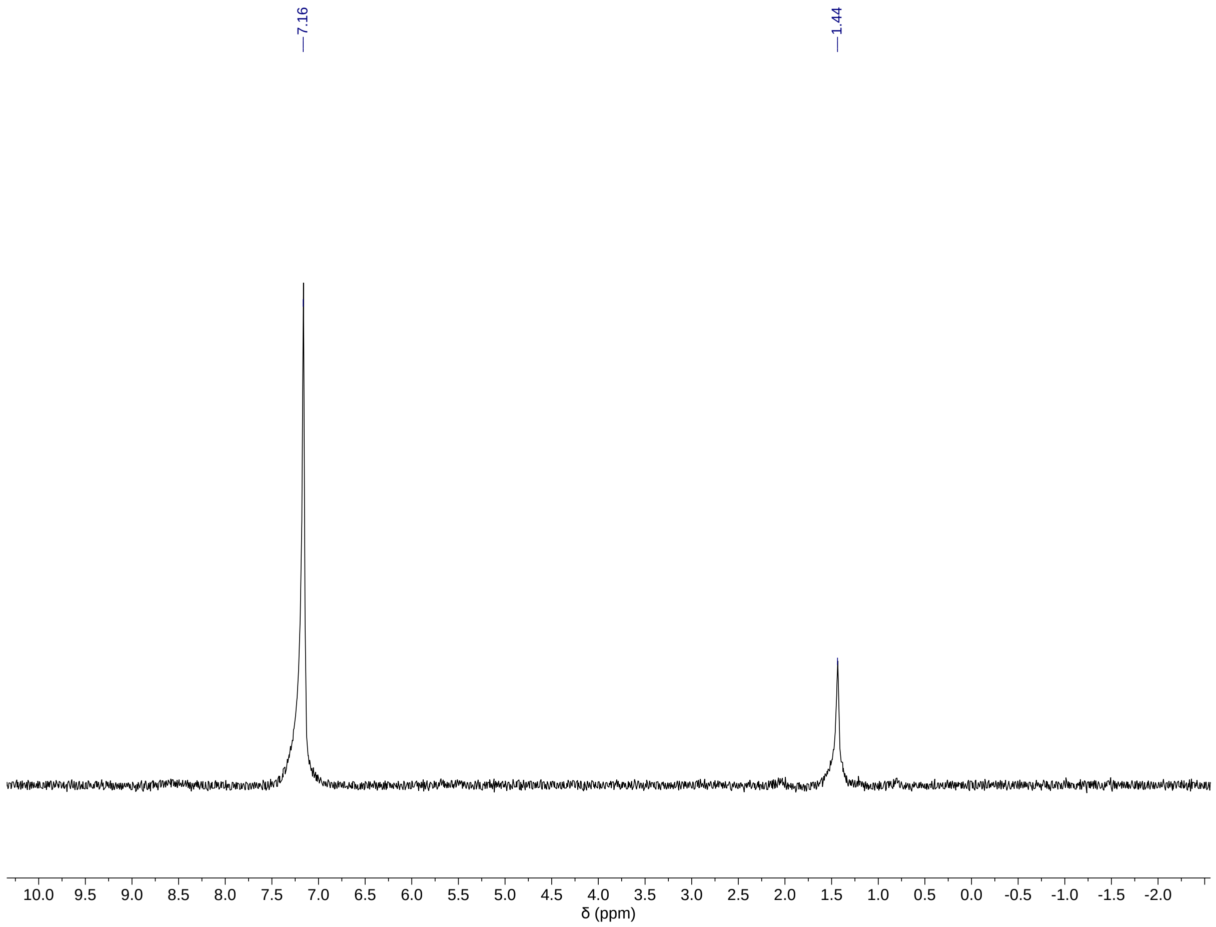

Figure S32. ^{2}H NMR (77 MHz) spectrum of the volatiles from the thermolysis of **3-Rh-d** in cyclooctane.

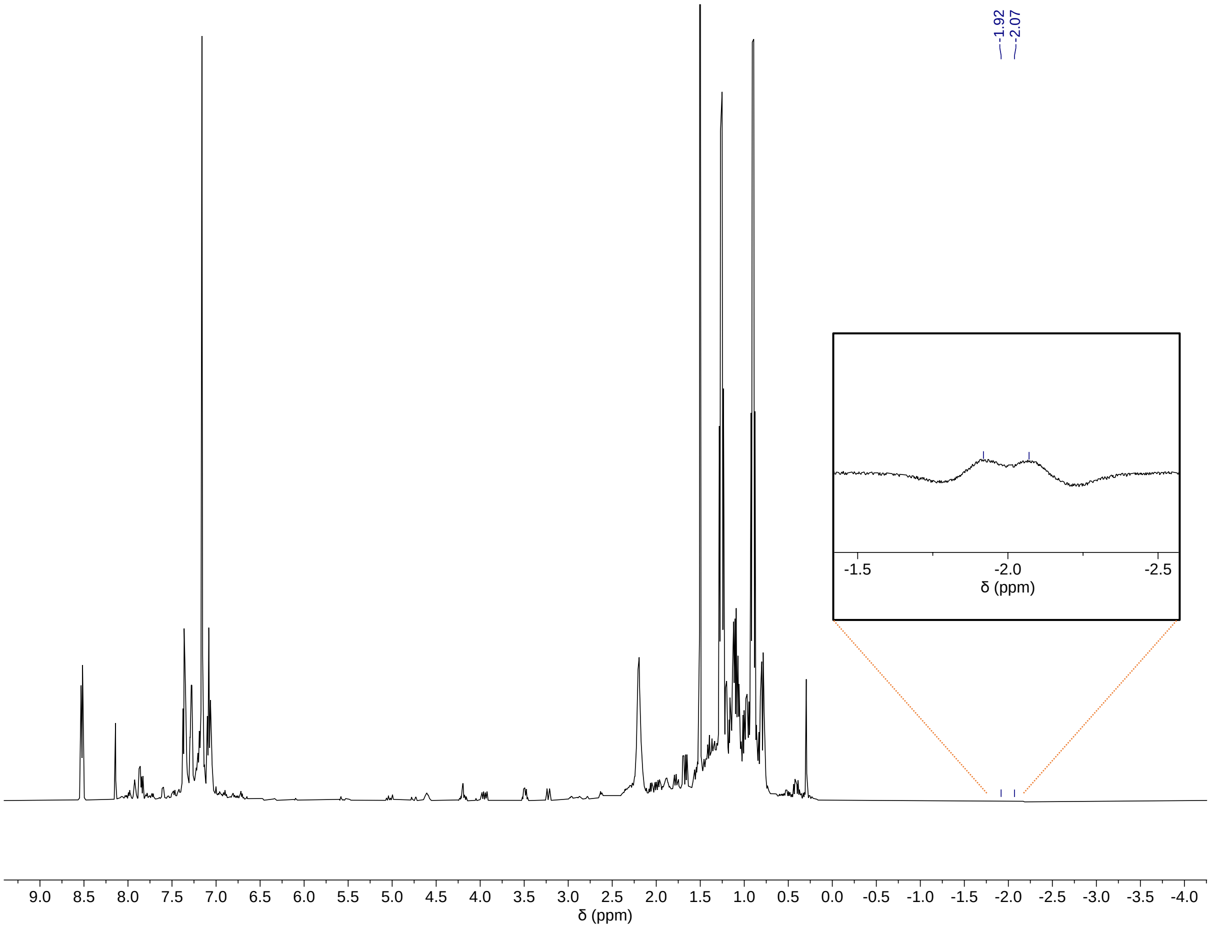

Figure S33. ^{1}H NMR (500 MHz, C_6D_6) spectrum of the non-volatiles from the thermolysis of **3-Rh-d** in cyclooctane. Sample contains residual cyclooctane and silicone grease.

Test for H/D exchange for the thermolysis of 3-Rh-d in mesitylene. In a J. Young tube, a 600 μL cyclooctane solution of **3-Rh-d** (20 mg, 0.033 mmol) was thermolyzed at 150 ˚C for 6 h. The volatiles were vacuum transferred into a separate J. Young tube. ^{2}H NMR analysis of the volatiles revealed the presence of deuterated mesitylene and benzene (Figure S34). ^{1}H NMR analysis of the non-volatiles revealed hydride signals corresponding to **9-Rh** (Figure S35), indicating H/D exchange with mesitylene.

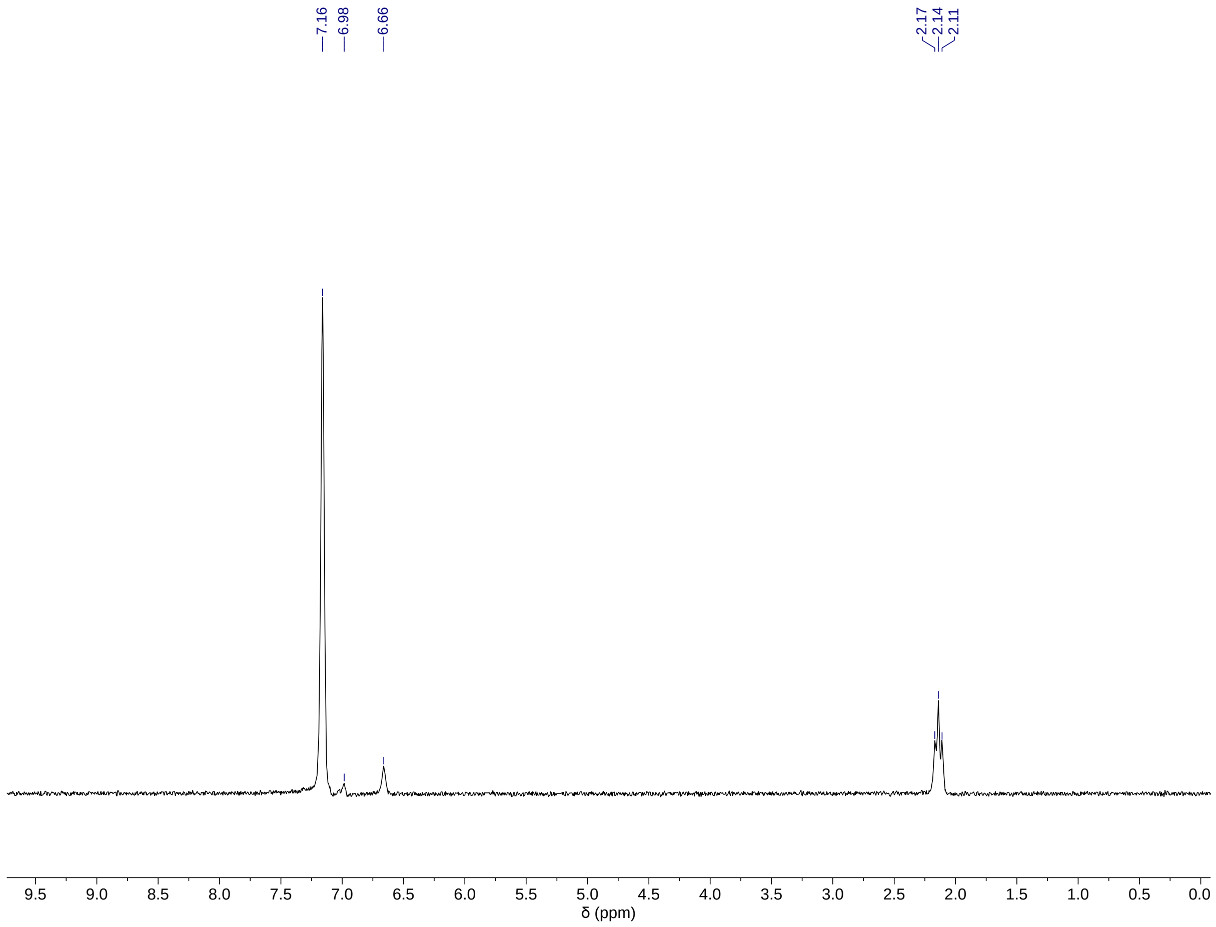

Figure S34. ^{2}H NMR (77 MHz) spectrum of the volatiles from the thermolysis of **3-Rh-d** in mesitylene.

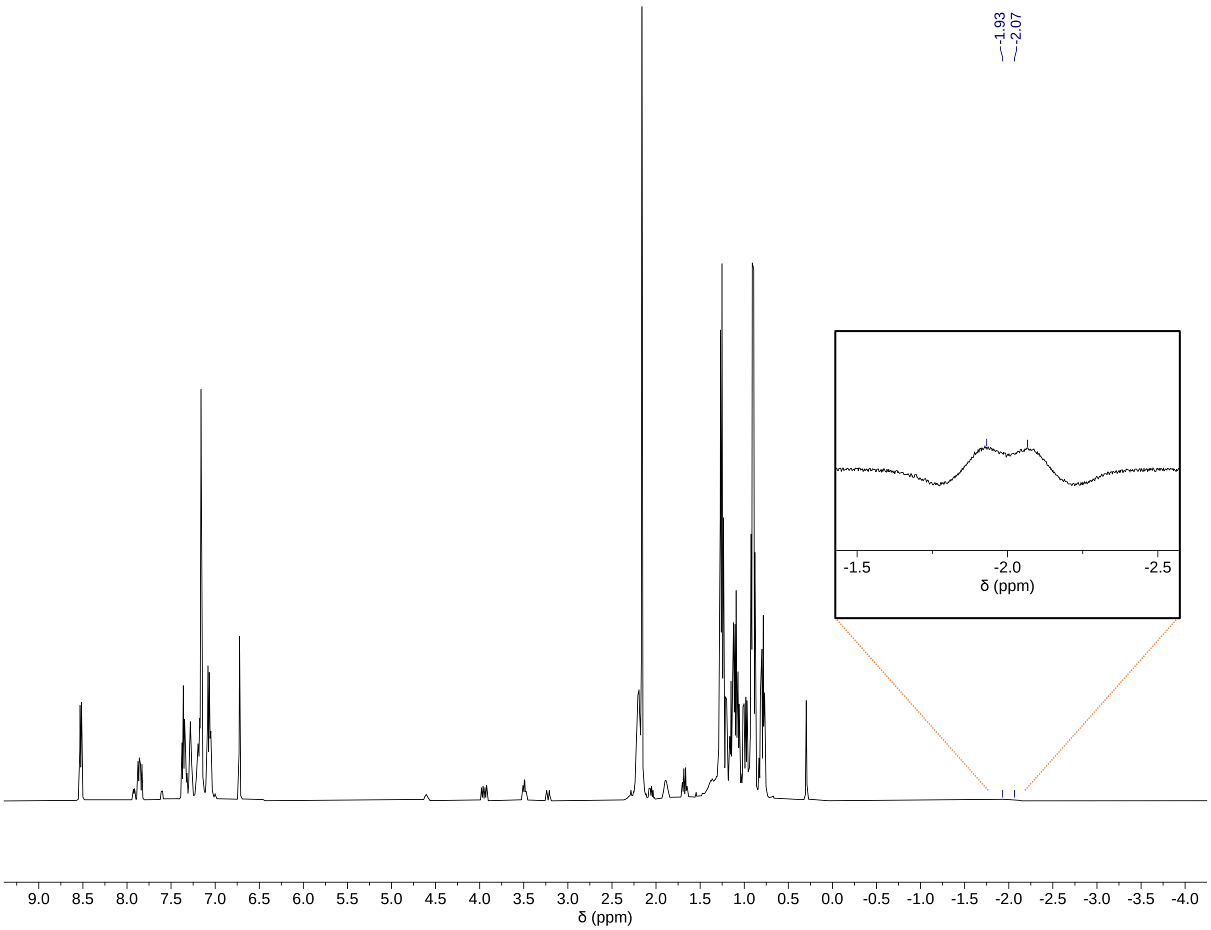

Figure S35. ^{1}H NMR (500 MHz, C_6D_6) spectrum of the non-volatiles from the thermolysis of **3-Rh-d** in mesitylene. Sample contains residual mesitylene and silicone grease.

7.3. KIE Experiments

Thermolysis of 3-Rh. Two parallel J. Young tube samples containing a 500 µL mesitylene-h_{12} solution of **3-Rh** (66 mM) and a capillary tube with $P(OPh)_3$ (33 µL, 0.016 mmol, 0.50 M in mesitylene-h_{12}) as an integration standard were prepared. The four samples were thermolyzed at 150 ˚C and monitored by $^{31}P\{^1H\}$ NMR spectroscopy in 45 min intervals for 7.5 h. The rate constants for the two *protio* thermolyses were determined to be 8.9(7) x 10^{-5} sec^{-1} and 10.9(8) x 10^{-5} sec^{-1}, providing an average rate constant of 9.9(3) x 10^{-5} sec^{-1}.

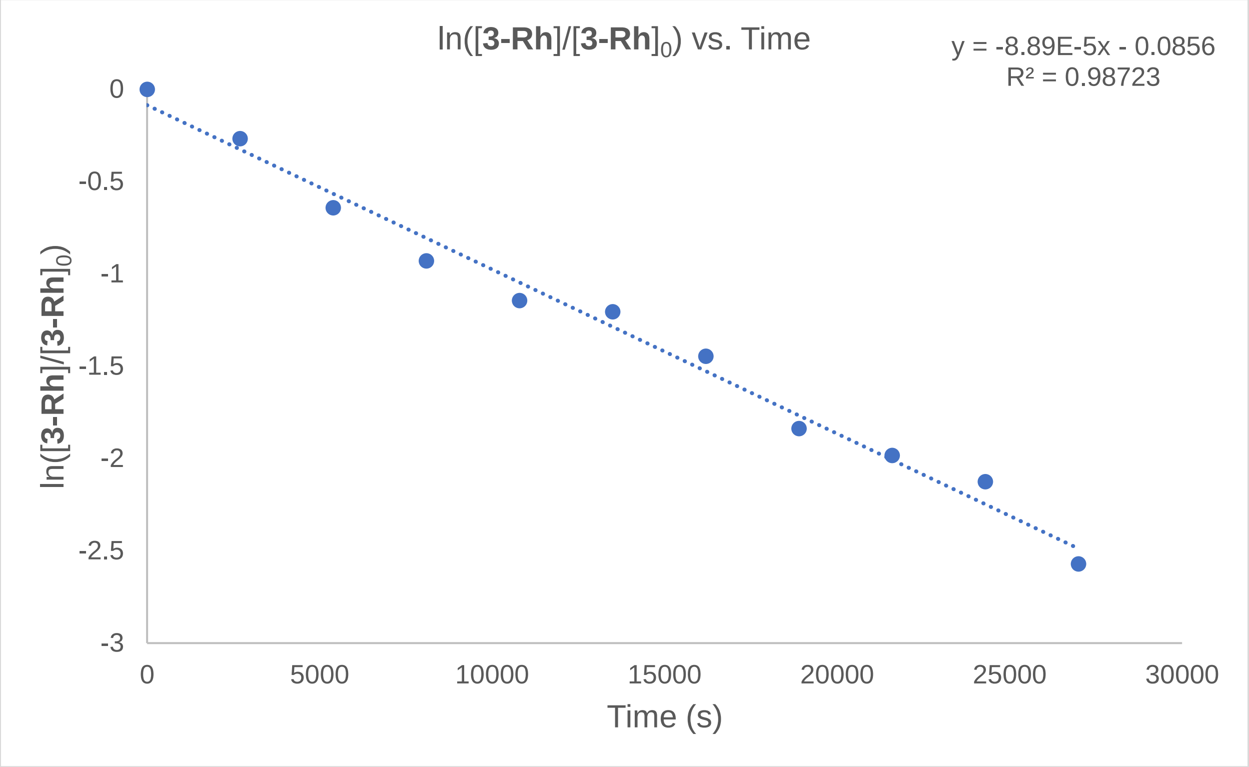

Figure S36. 1st order integrated rate law plot for the thermolysis of **3-Rh** (trial 1).

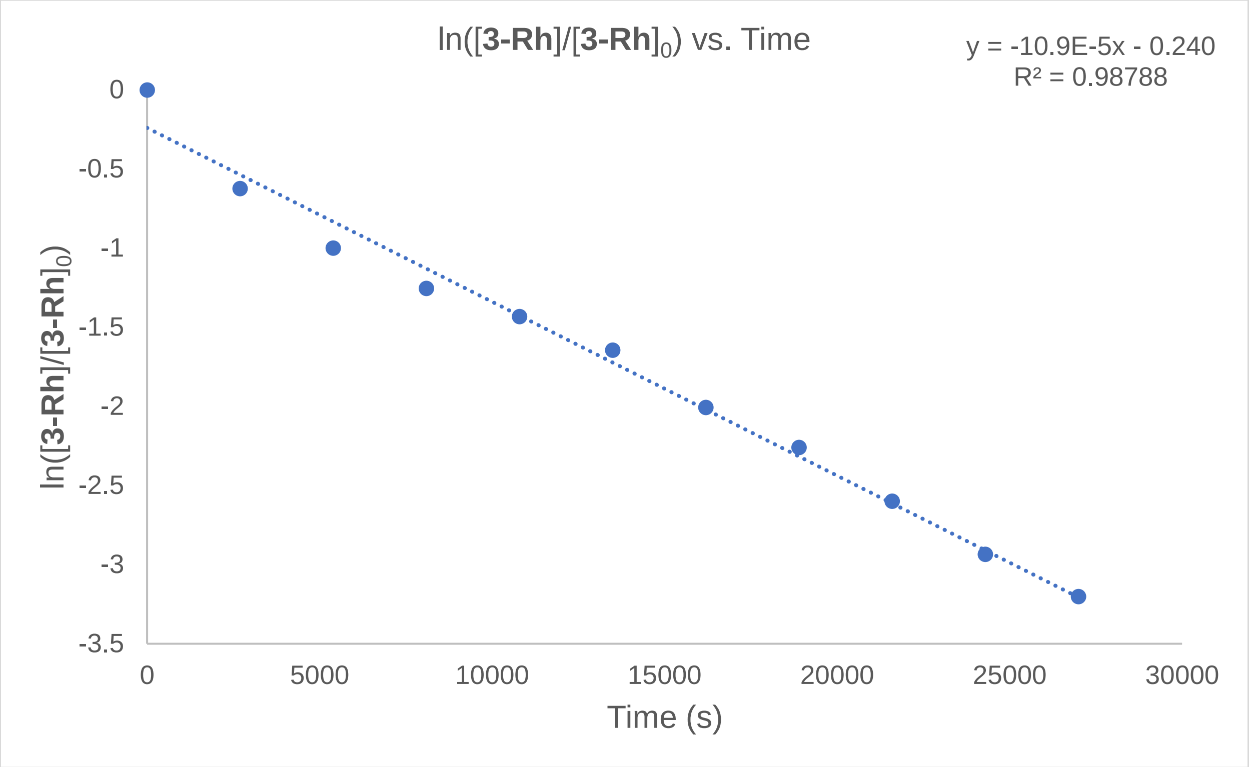

Figure S37. 1st order integrated rate law plot for the thermolysis of **3-Rh** (trial 2).

Thermolysis of 3-Rh-d. Two parallel J. Young tube samples containing a 500 µL mesitylene-d_{12} solution of **3-Rh-d** (66 mM) and a capillary tube with $P(OPh)_3$ (33 µL, 0.016 mmol, 0.50 M in mesitylene-h_{12}) as an integration standard were prepared. The four samples were thermolyzed at 150 ˚C and monitored by $^{31}P\{^{1}H\}$ NMR spectroscopy in 45 min intervals for 7.5 h. The rate constants for the two *deutero* thermolyses were determined to be 6.1(4) x 10^{-5} sec^{-1} and 5.9(5) x 10^{-5} sec^{-1}, providing an average rate constant of 6.0(2) x 10^{-5} sec^{-1}.

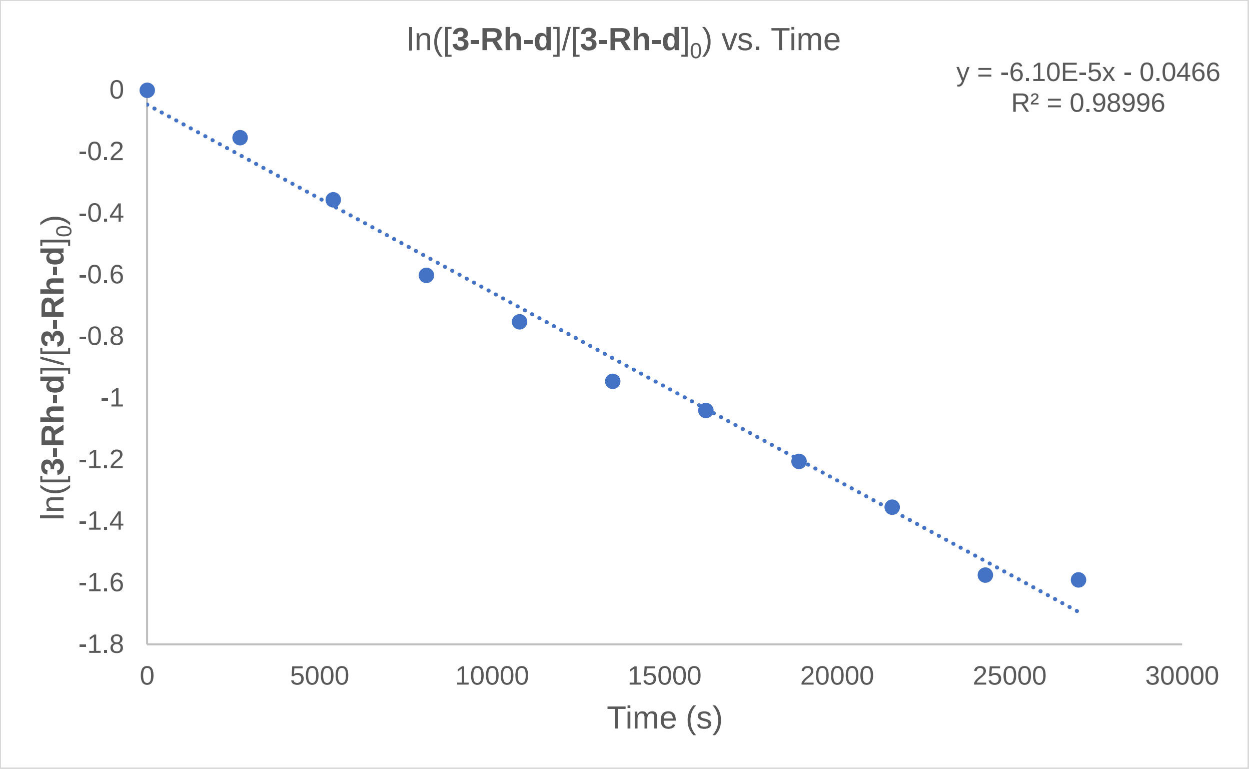

Figure S38. 1st order integrated rate law plot for the thermolysis of **3-Rh-d** (trial 1).

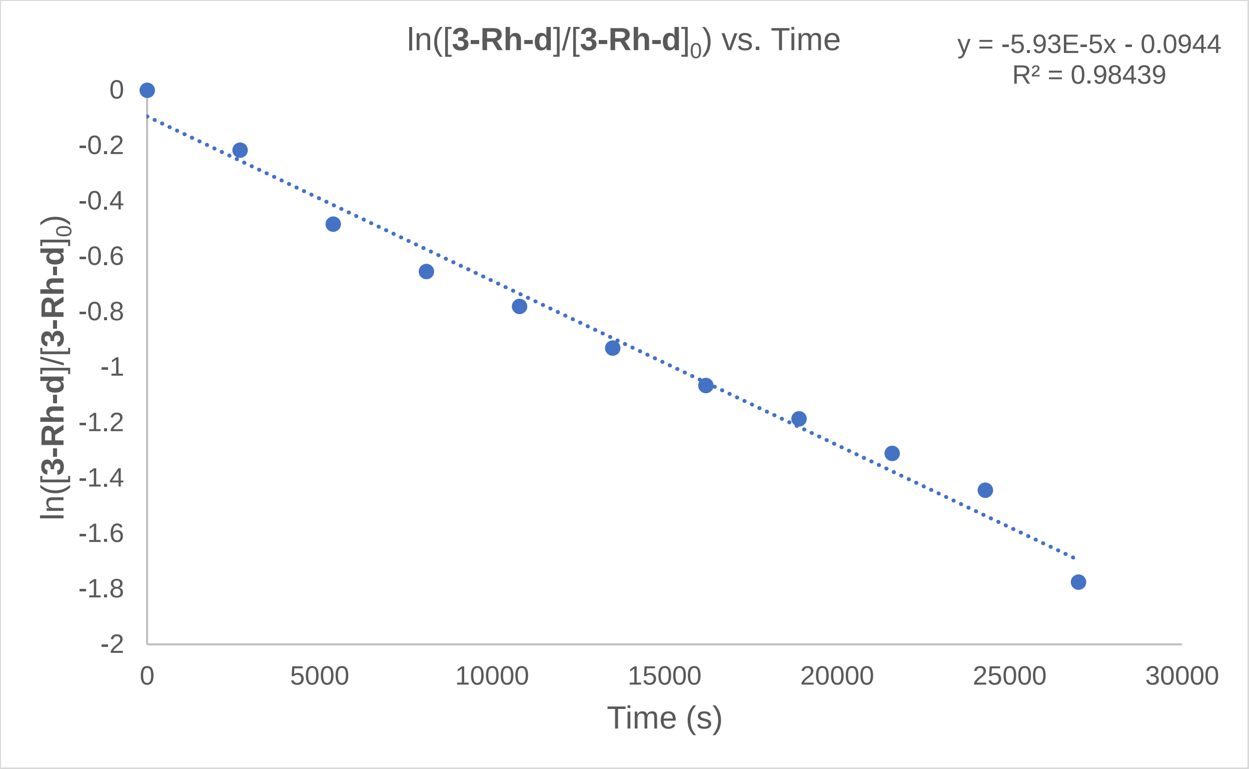

Figure S39. 1st order integrated rate law plot for the thermolysis of **3-Rh-d** (trial 2).

Based on the average rate constants for the *protio* and *deutero* thermolysis, KIE was determined to be 1.62(15).

8. Additional NMR Spectra for Ir Compounds.

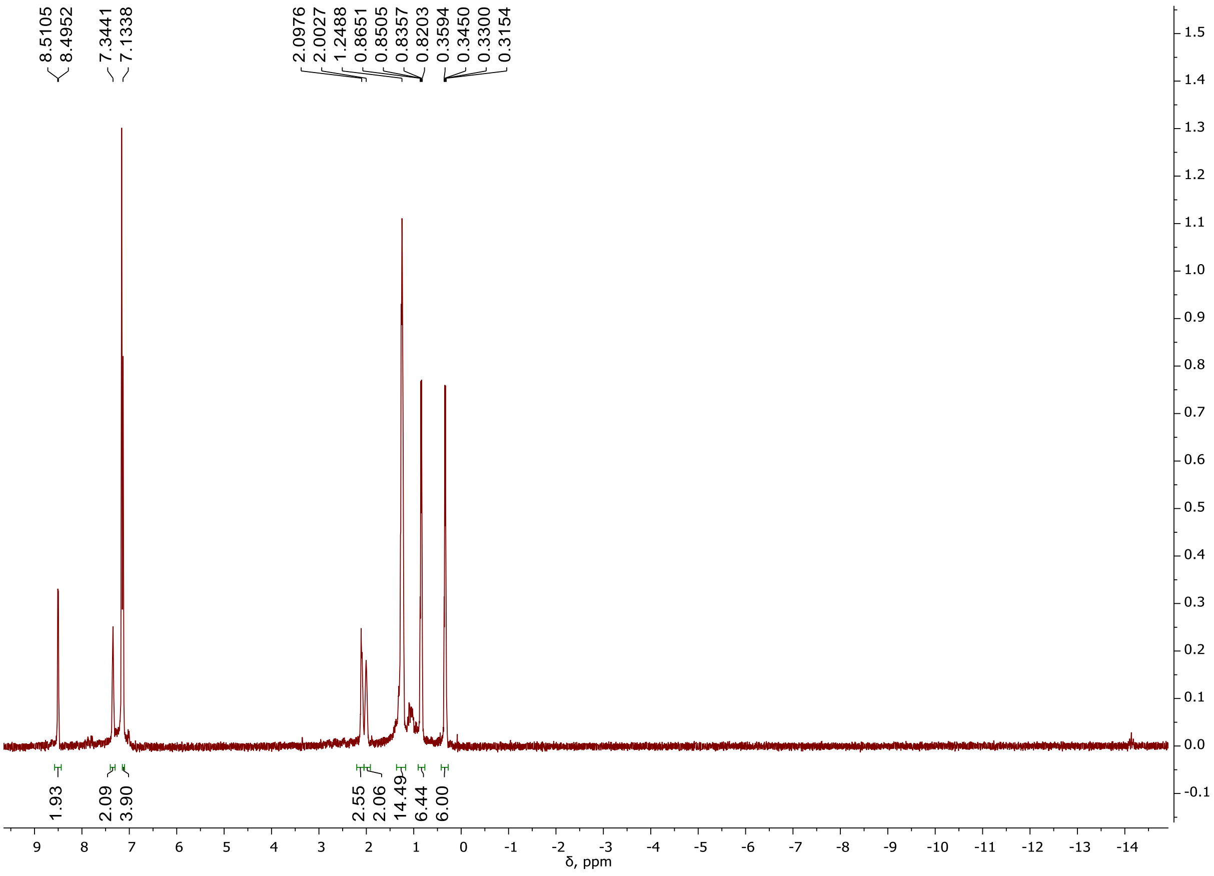

Figure S40. ^{1}H NMR (500 MHz, C_6D_6) spectrum of **2d**.

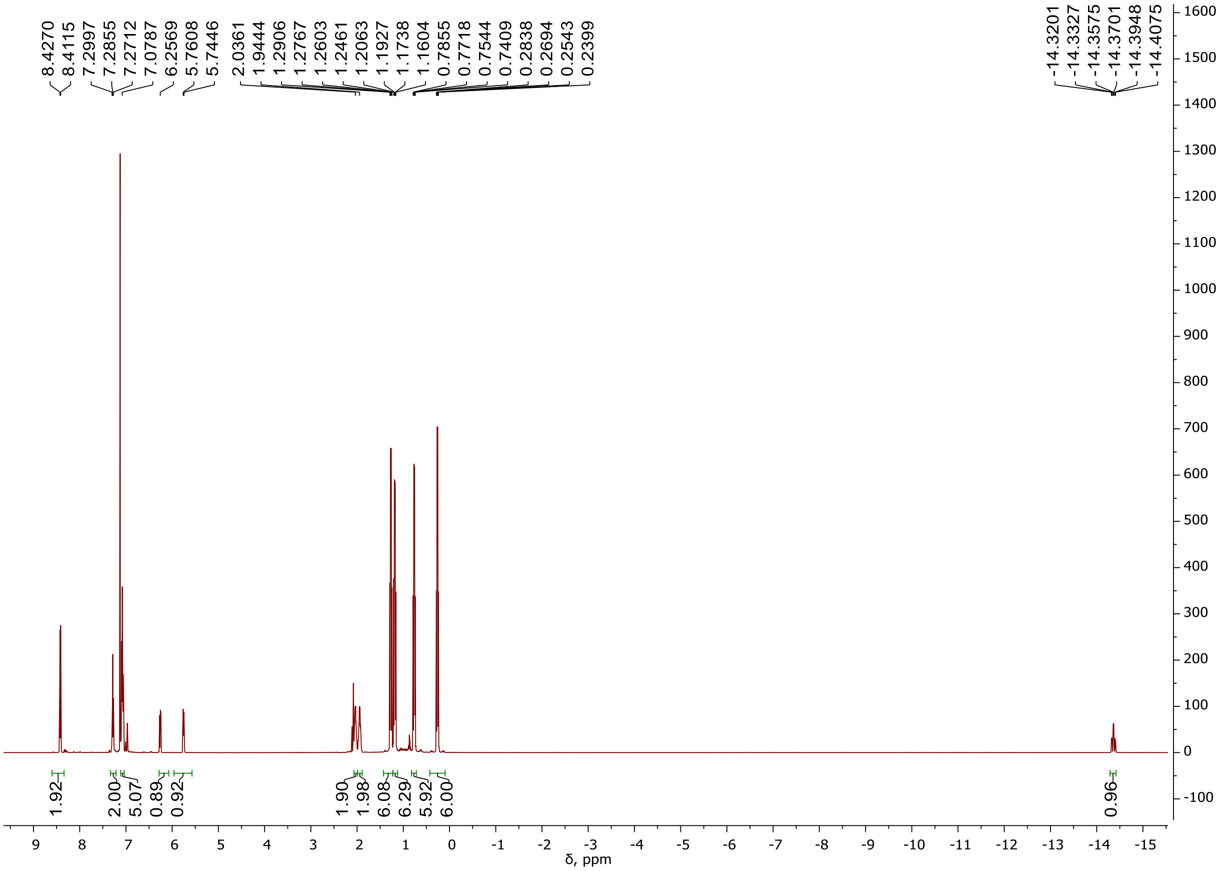

Figure S41. ^{1}H NMR (500 MHz, C_7D_8) spectrum of **2e**.

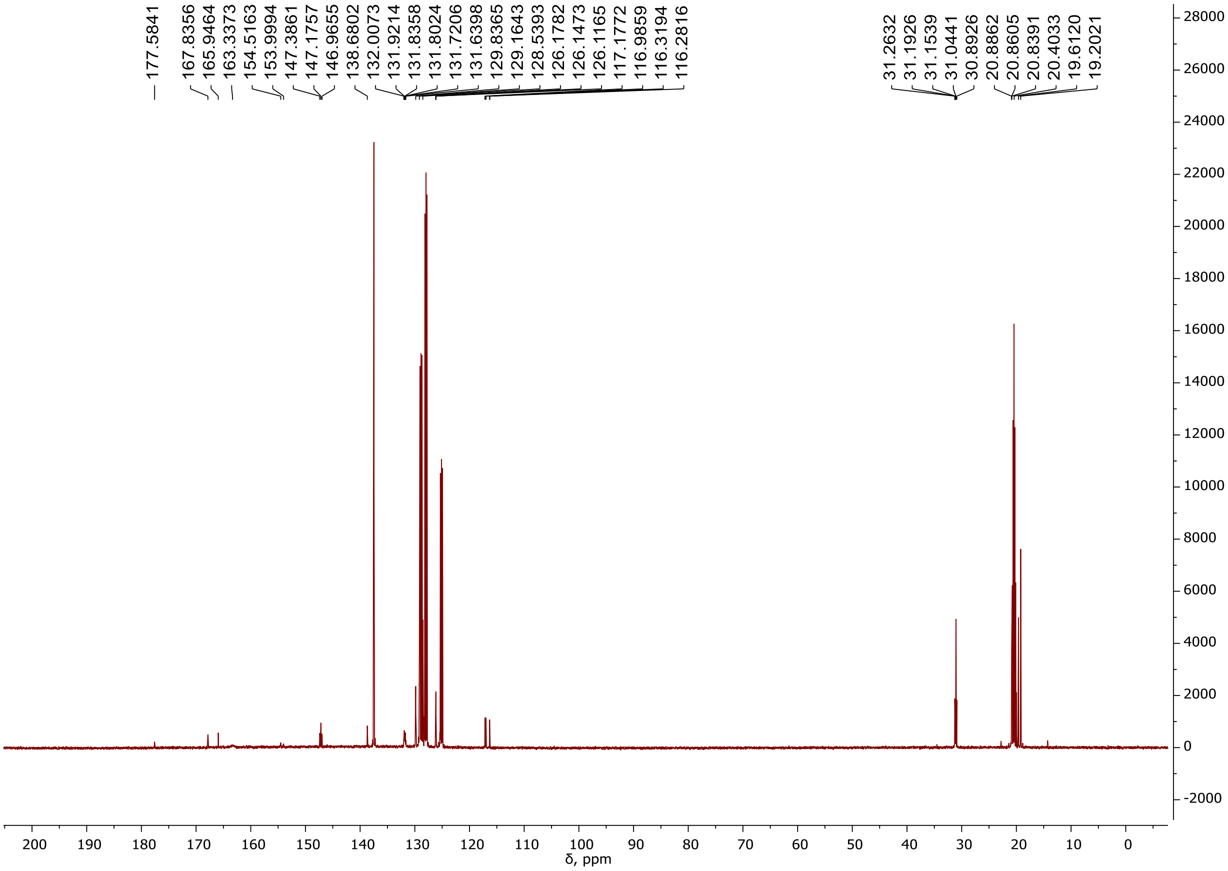

Figure S42. $^{13}C\{^{1}H\}$ NMR (126 MHz, C_7D_8) spectrum of **2e**.

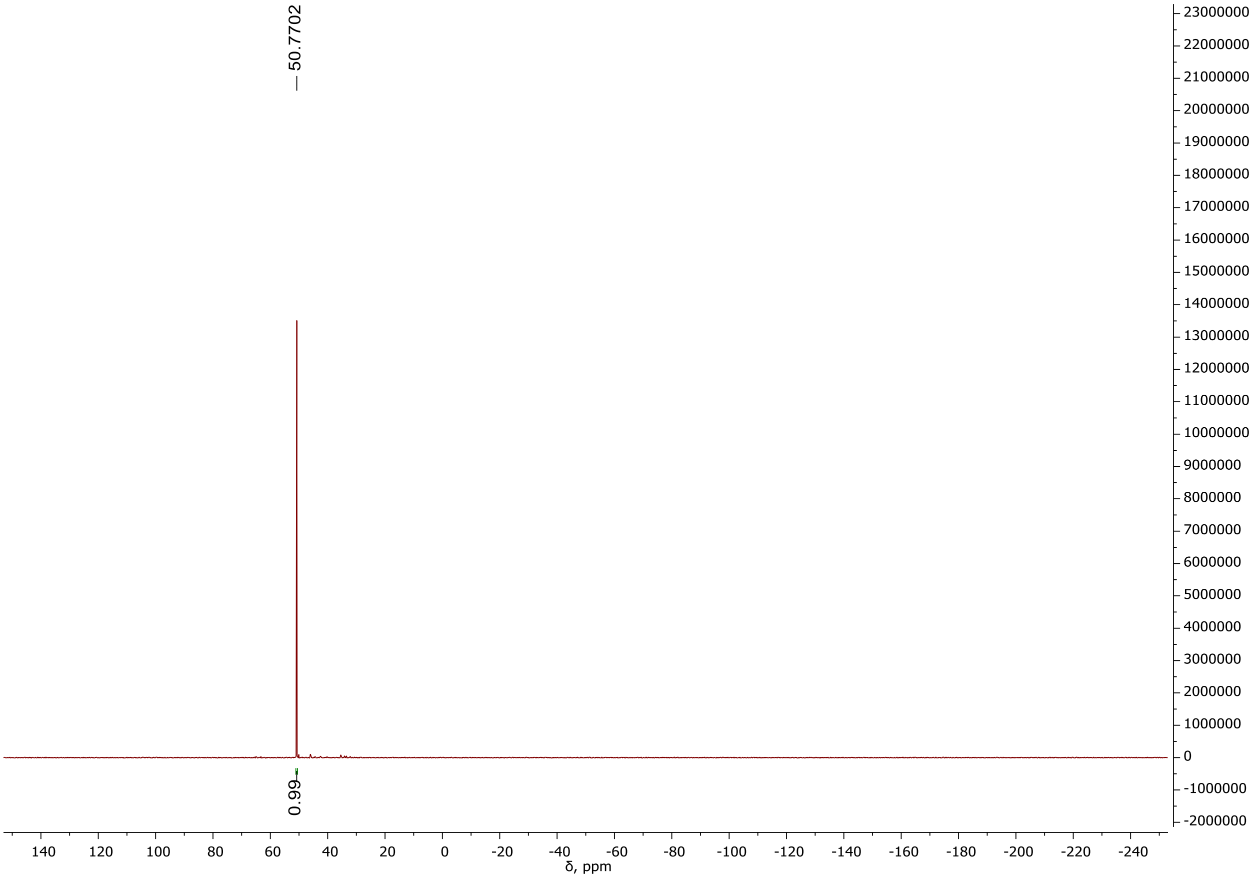

Figure S43. $^{31}P\{^1H\}$ (202 MHz, C_7D_8) NMR spectrum of **2e**.

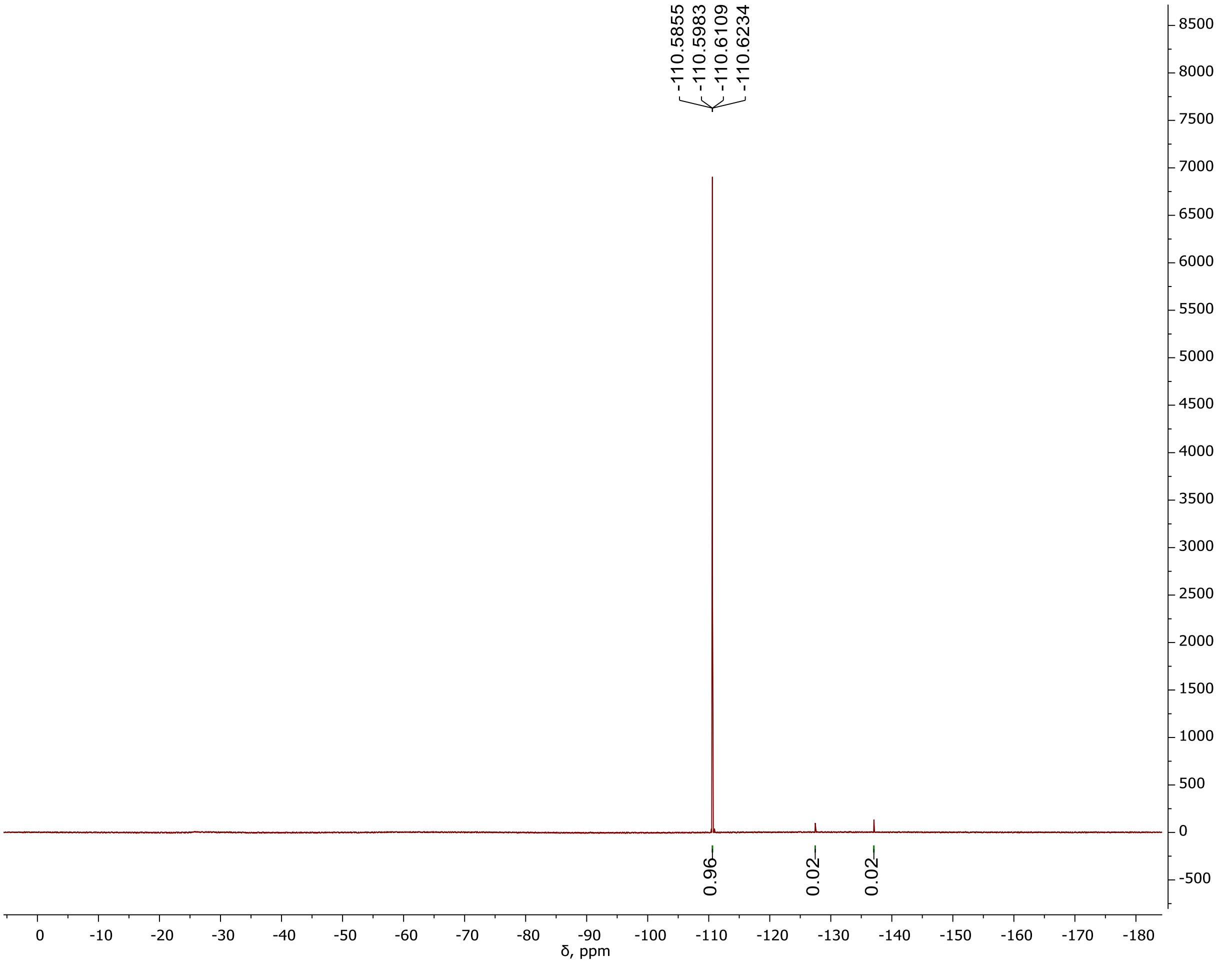

Figure S44. ^{19}F NMR (471 MHz, C_7D_8) spectrum of **2e**.

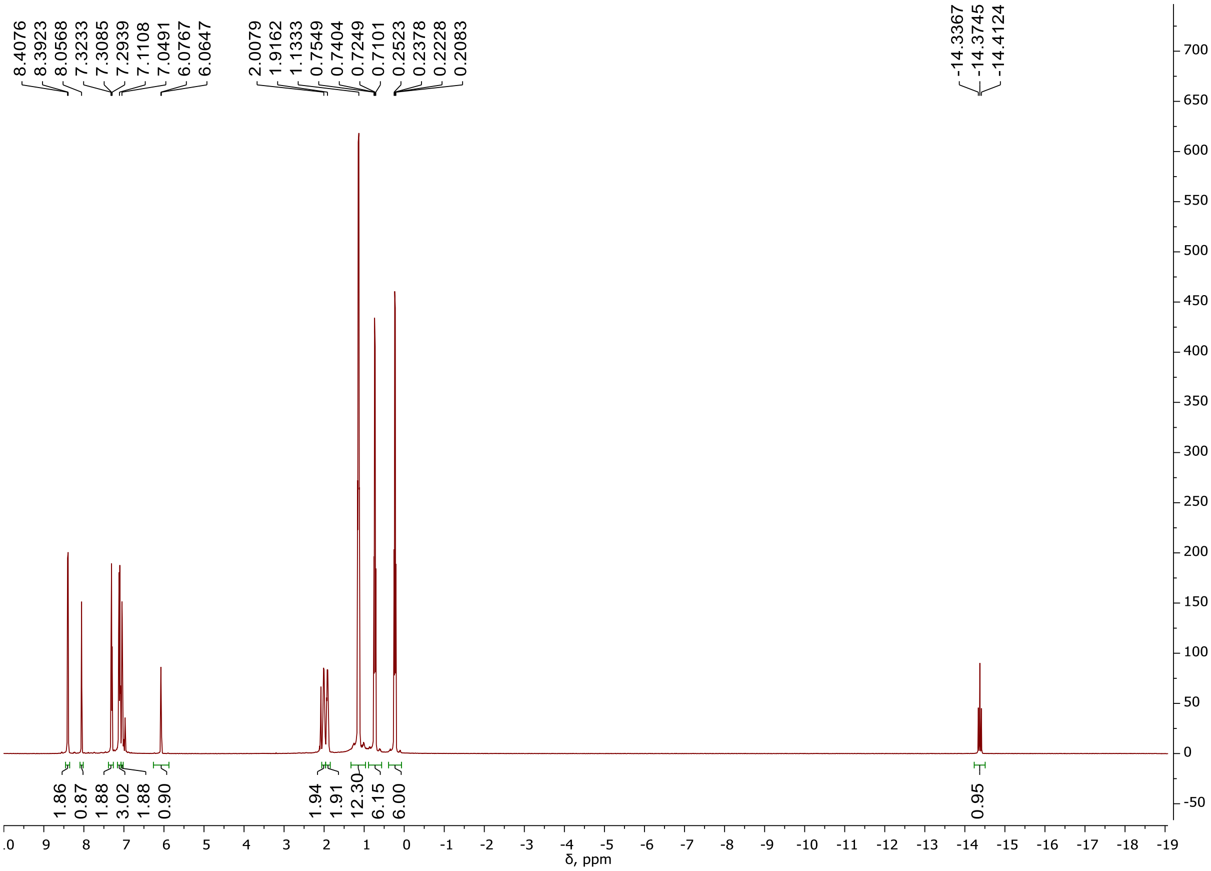

Figure S45. ^{1}H NMR (500 MHz, C_7D_8) spectrum of **3**.

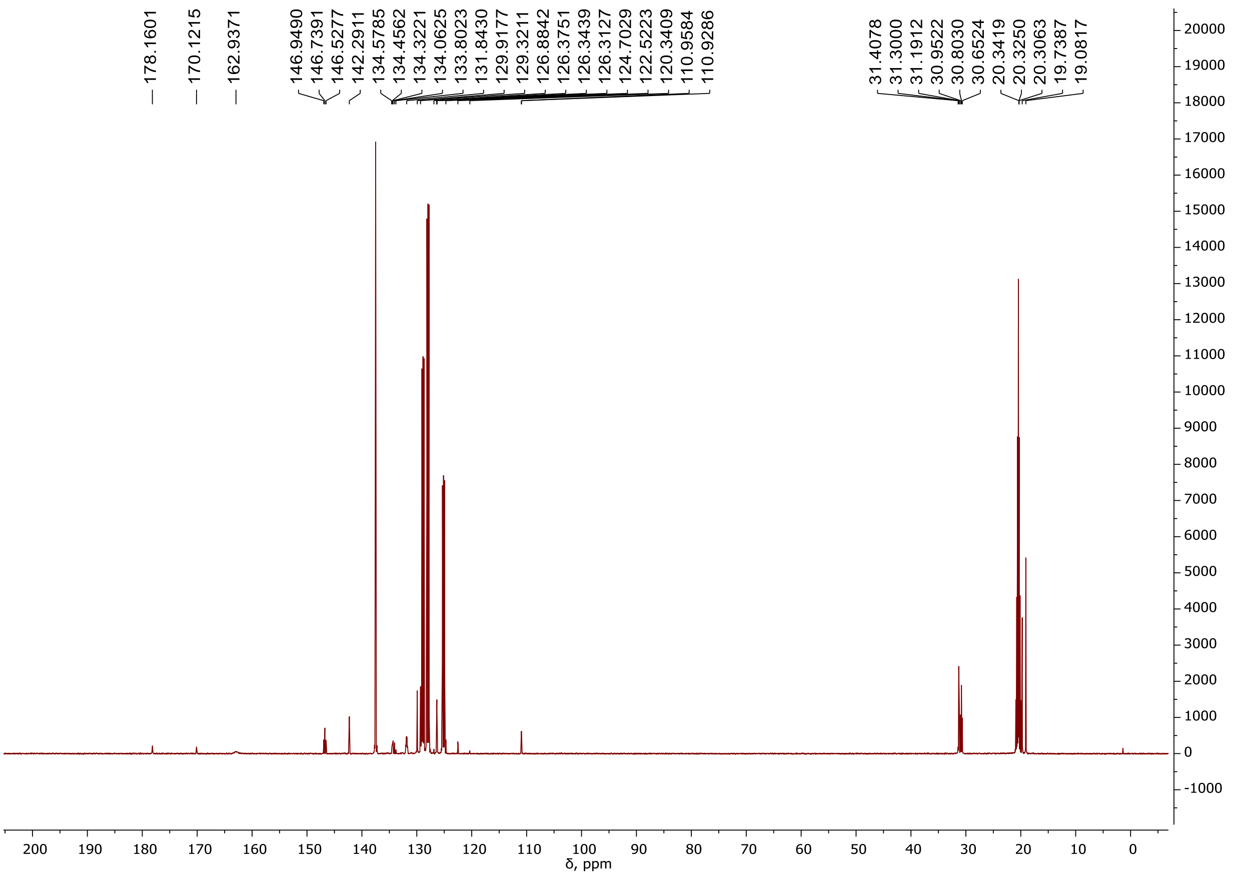

Figure S46. $^{13}C\{^{1}H\}$ NMR (126 MHz, C_7D_8) spectrum of **3**.

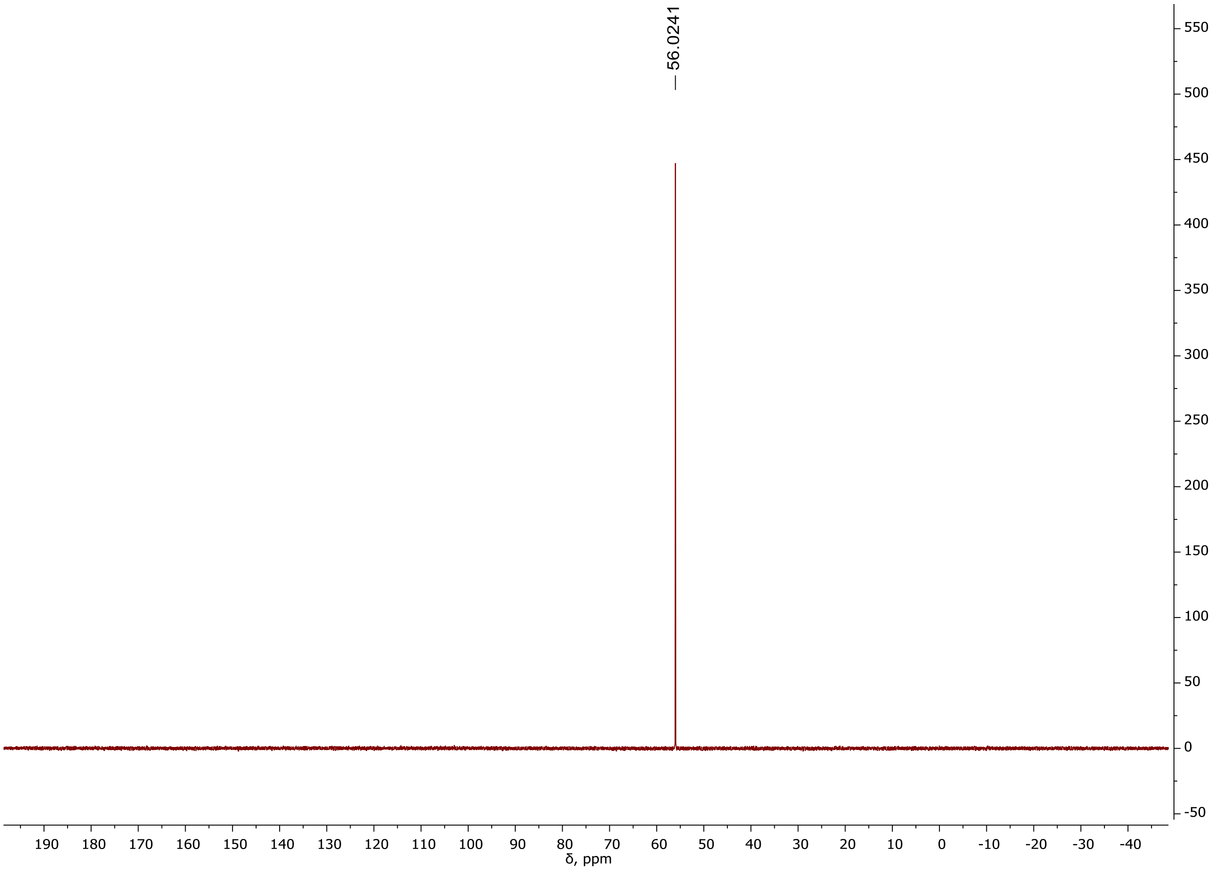

Figure S47. $^{31}P\{^{1}H\}$ (202MHz, C_7D_8) NMR spectrum of **3**.

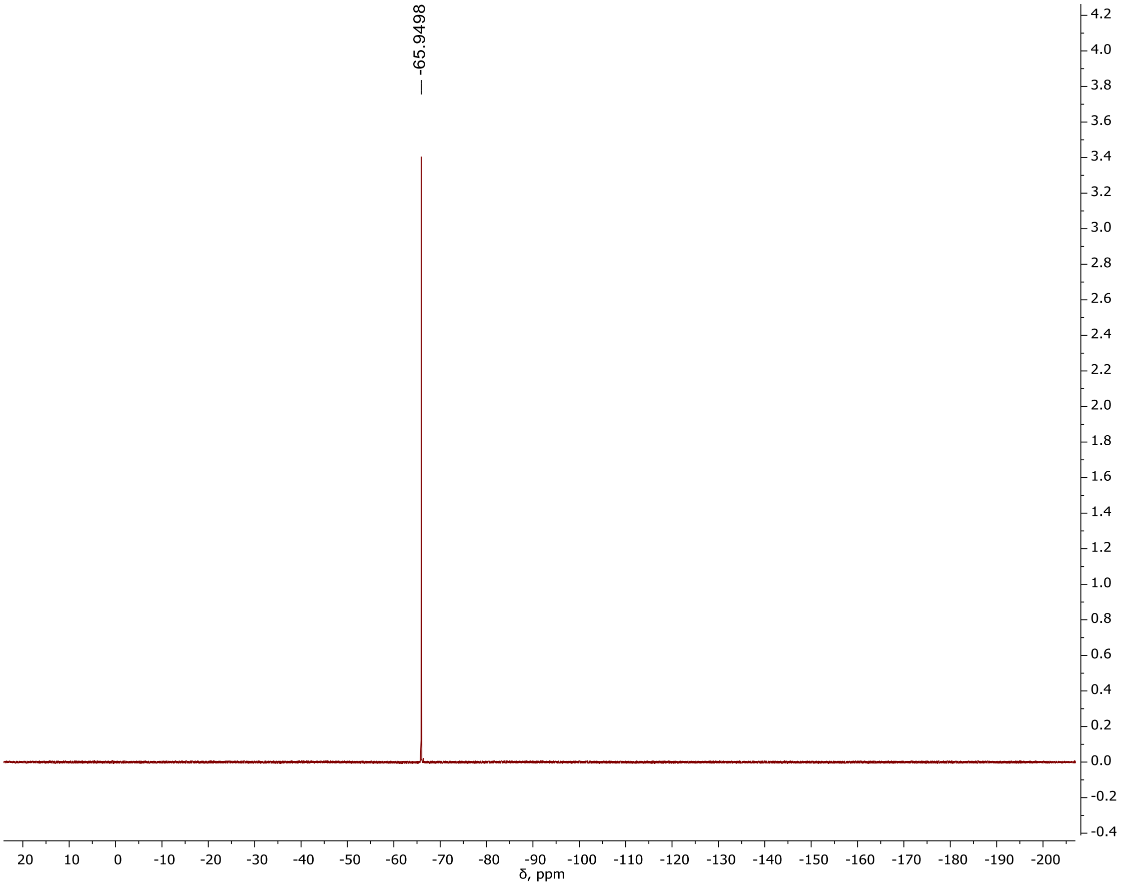

Figure S48. ^{19}F NMR (471 MHz, C_7D_8) spectrum of **3**.

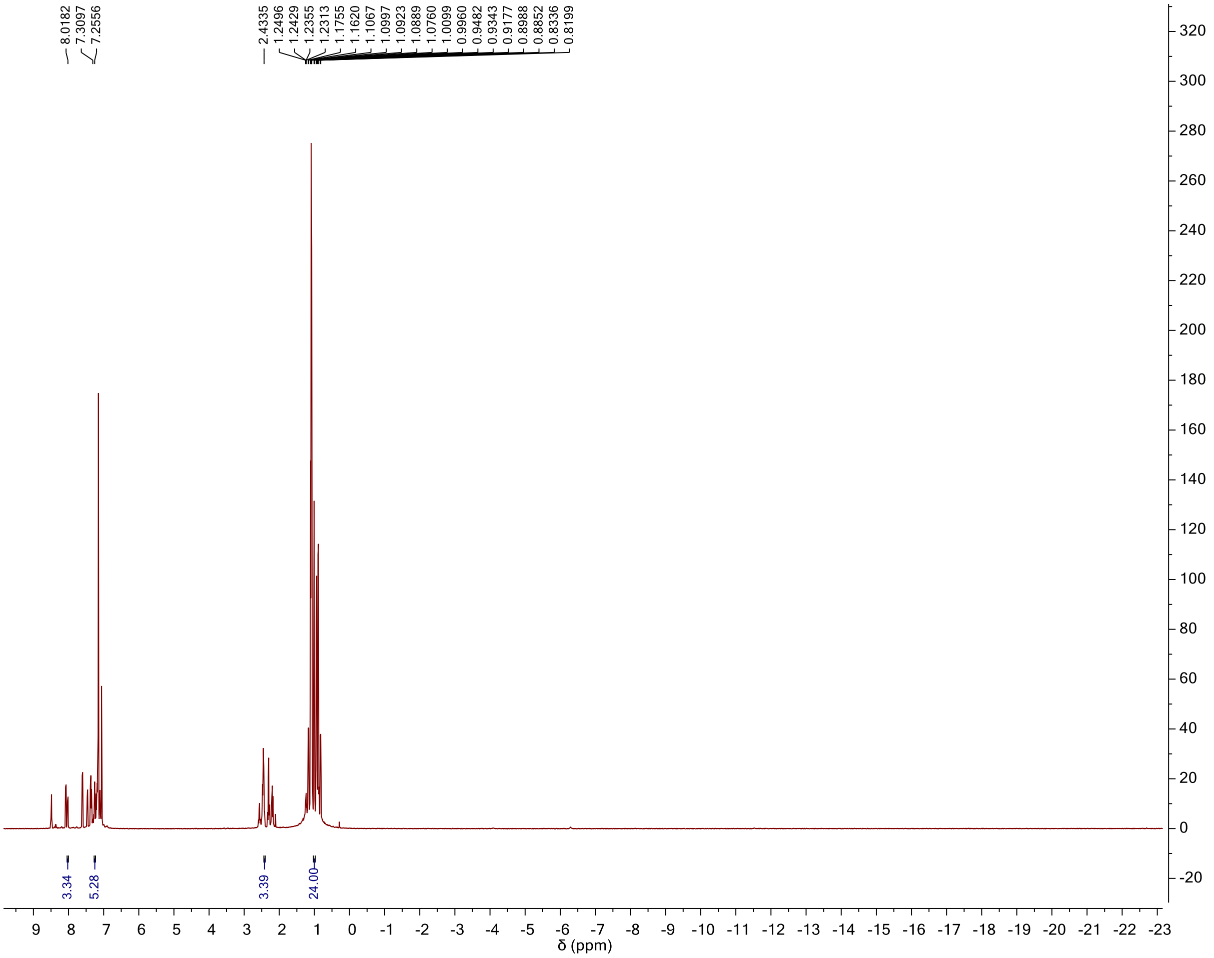

Figure S49. $^{1}H\{^{31}P\}$ NMR (500 MHz, C_6D_6) spectrum of the thermolysis of **3** at 100 ºC in C_6D_6 for 2 h with the *in situ* observation of **4**.

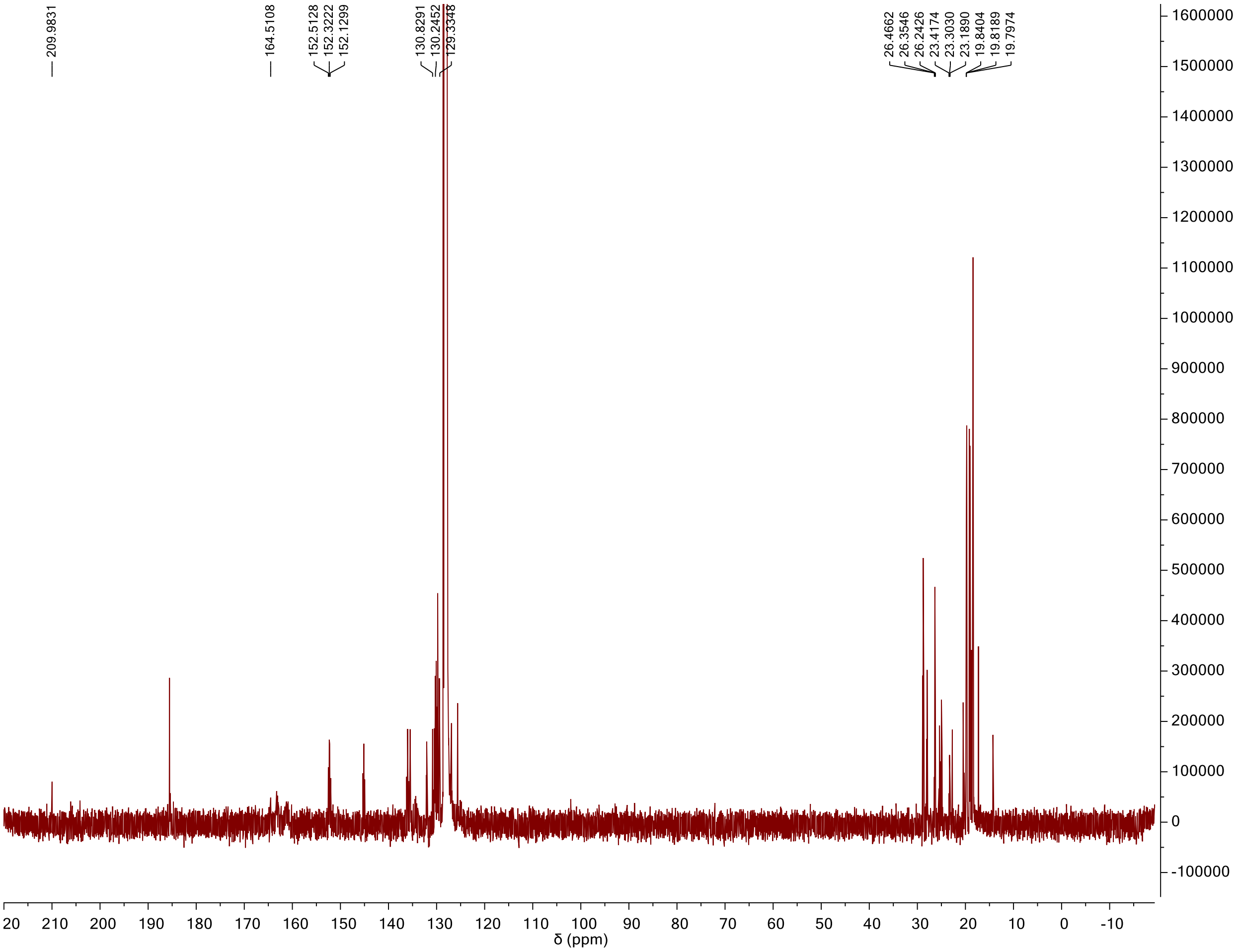

Figure S50. $^{13}C\{^1H\}$ NMR (126 MHz, C_6D_6) spectrum of the thermolysis of **3** at 100 ºC in C_6D_6 for 2 h with the *in situ* observation of **4**.

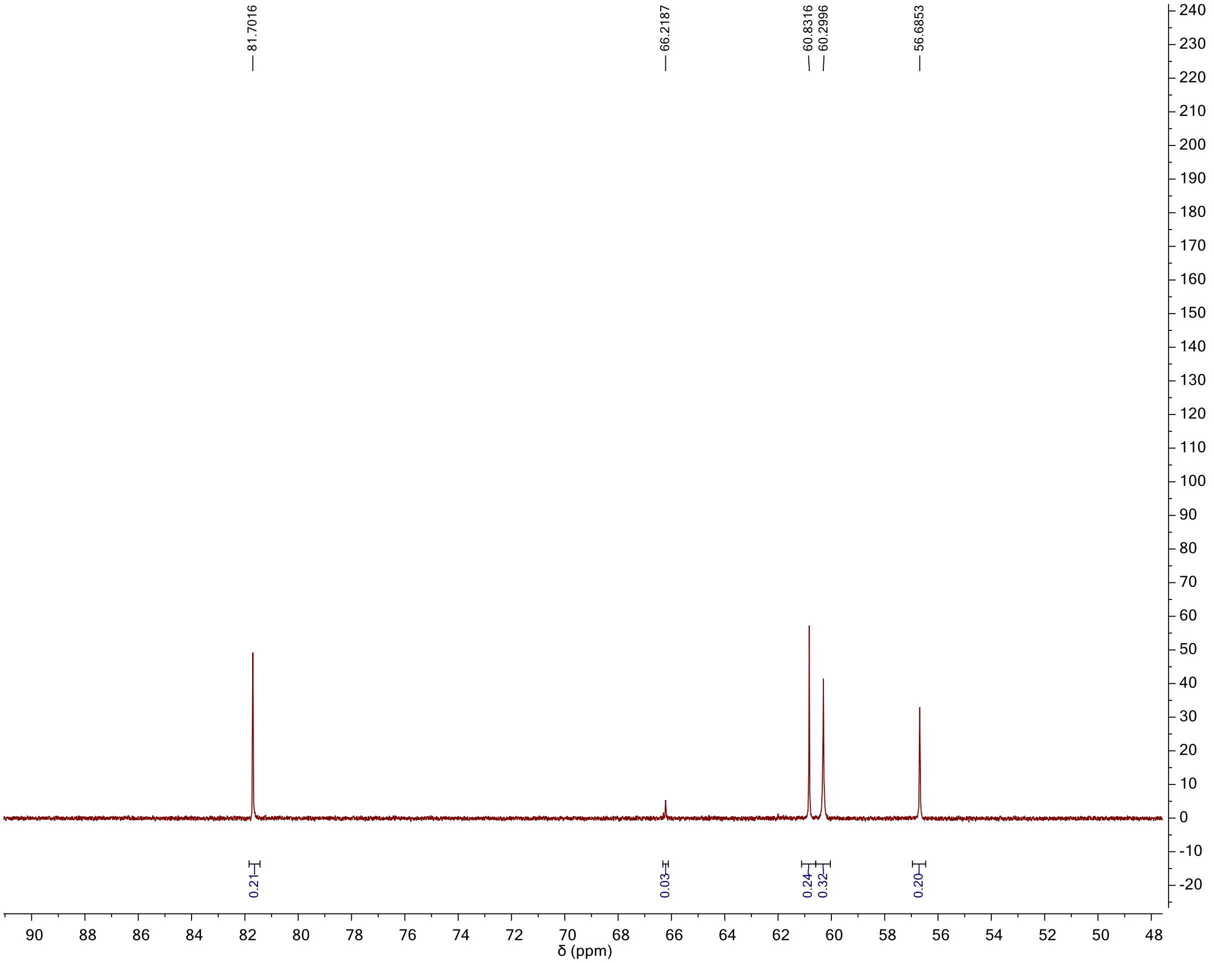

Figure S51. $^{31}P\{^{1}H\}$ NMR (202 MHz, C_6D_6) spectrum of the thermolysis of **3** at 100 ºC in C_6D_6 for 2 h with the *in situ* observation of **4**.

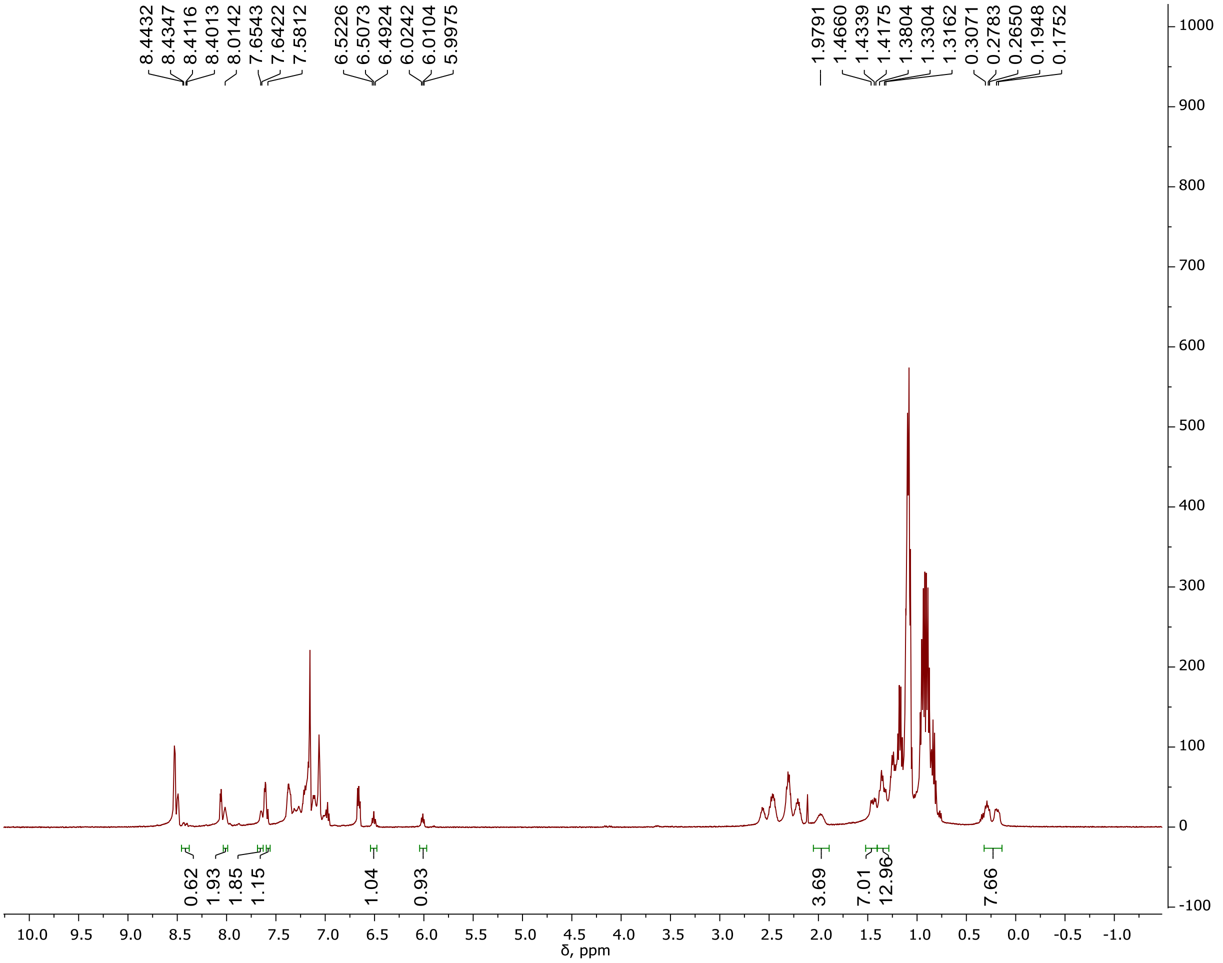

Figure S52. Expanded ^{1}H NMR (500 MHz, C_6D_6) spectrum of the thermolysis of **3** at 100 ºC for 15 h, followed by py addition. Peaks belonging to **12** were annotated.

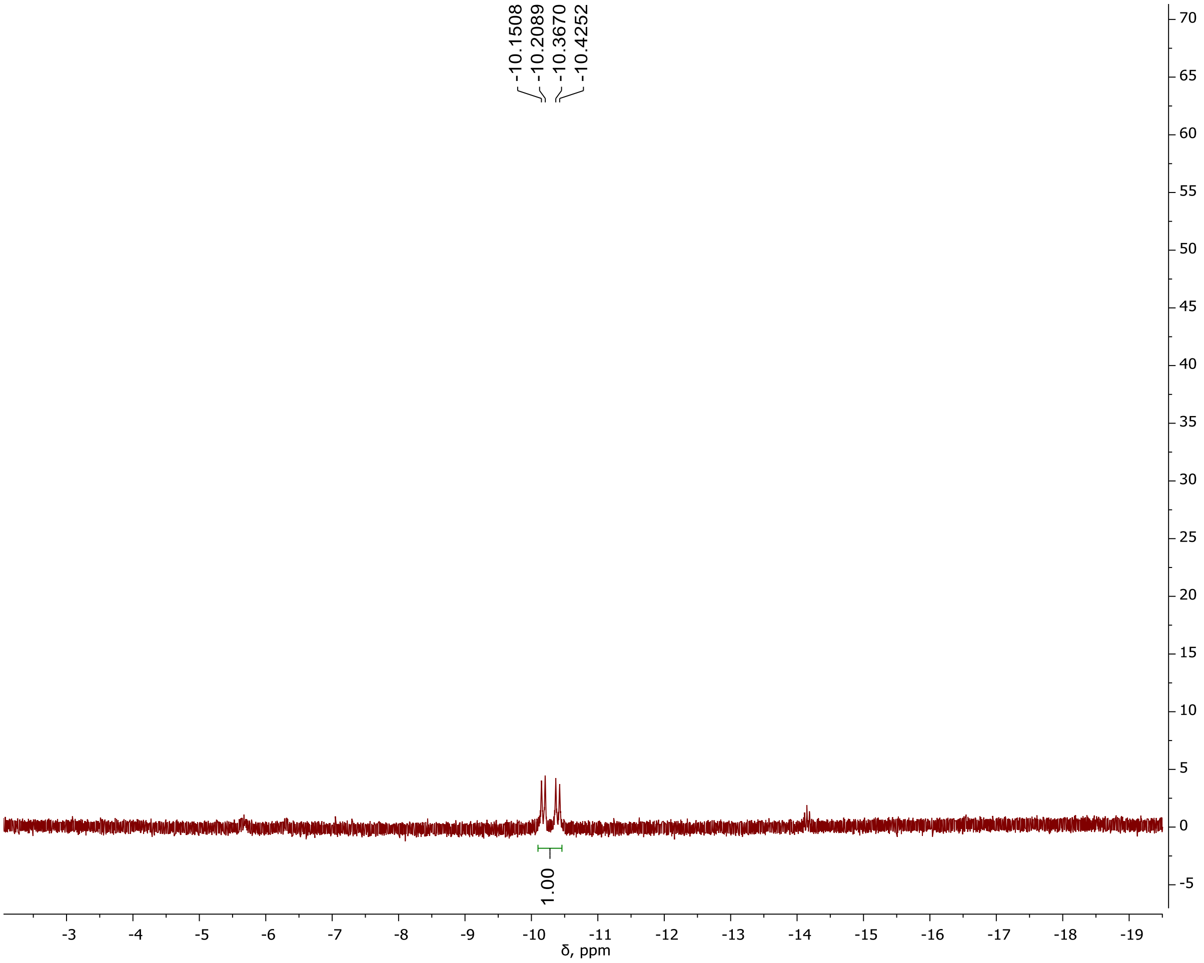

Figure S53. Expanded ^{1}H NMR (500 MHz, C_6D_6) spectrum of the thermolysis of **3** at 100 ºC for 15 h, followed by py addition. Peaks belonging to **12** were annotated.

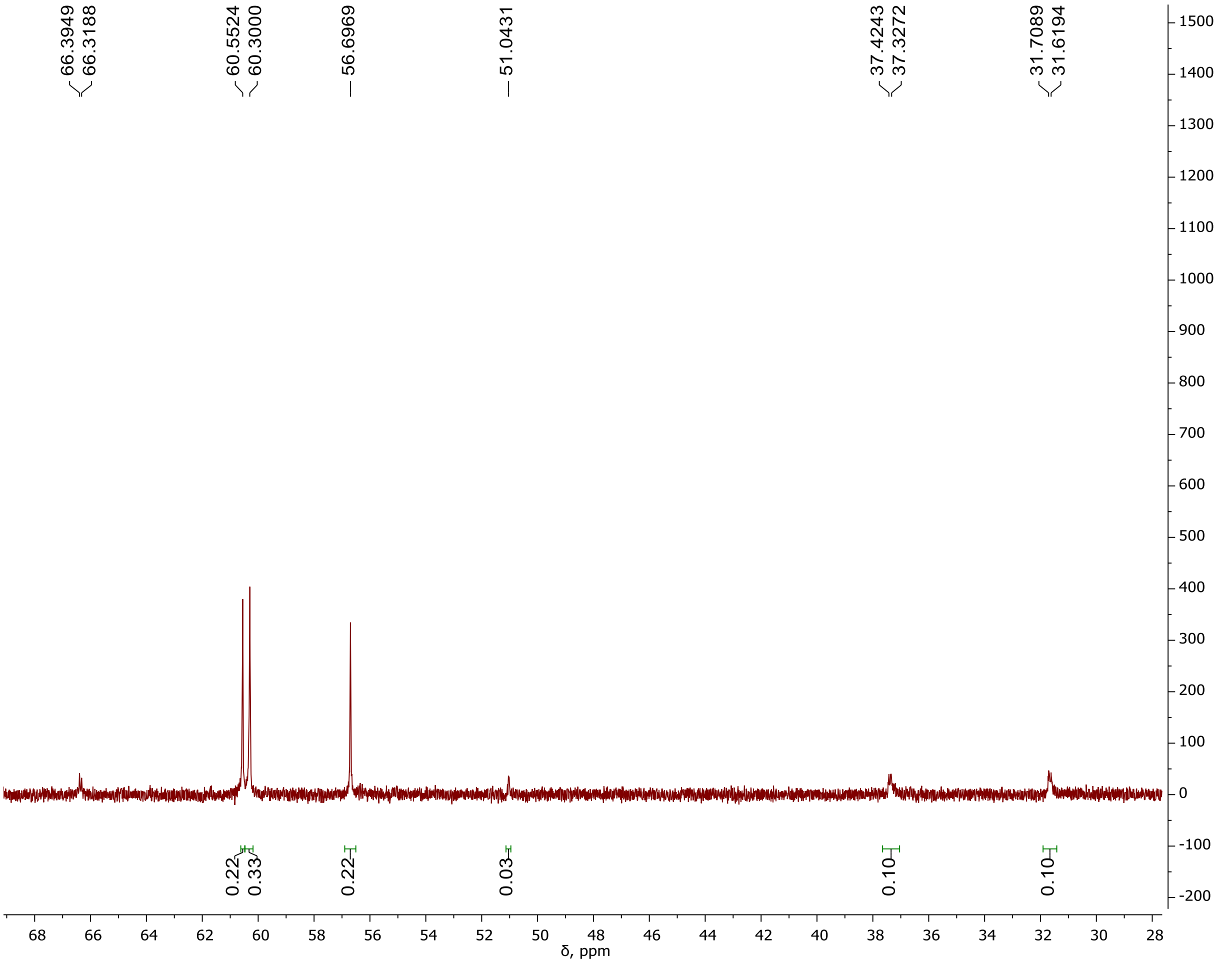

Figure S54. Expanded $^{31}P\{^{1}H\}$ NMR (202 MHz, C_6D_6) spectrum of the thermolysis of **3** at 100 ^{o}C for 15 h, followed by py addition.

9. Additional NMR Spectra for Rh Compounds.

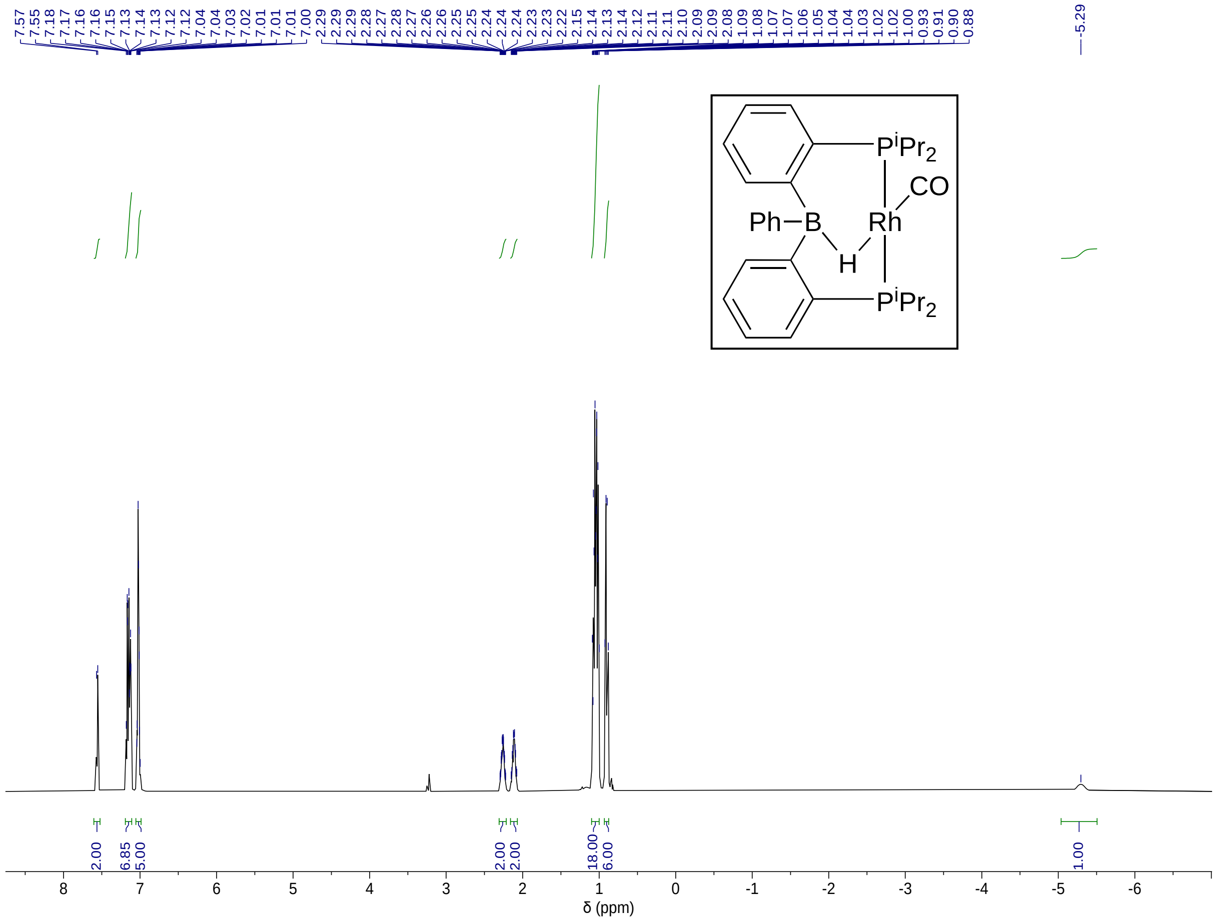

Figure S55. ^{1}H NMR (500 MHz) spectrum of **3-Rh** in C_6D_6.

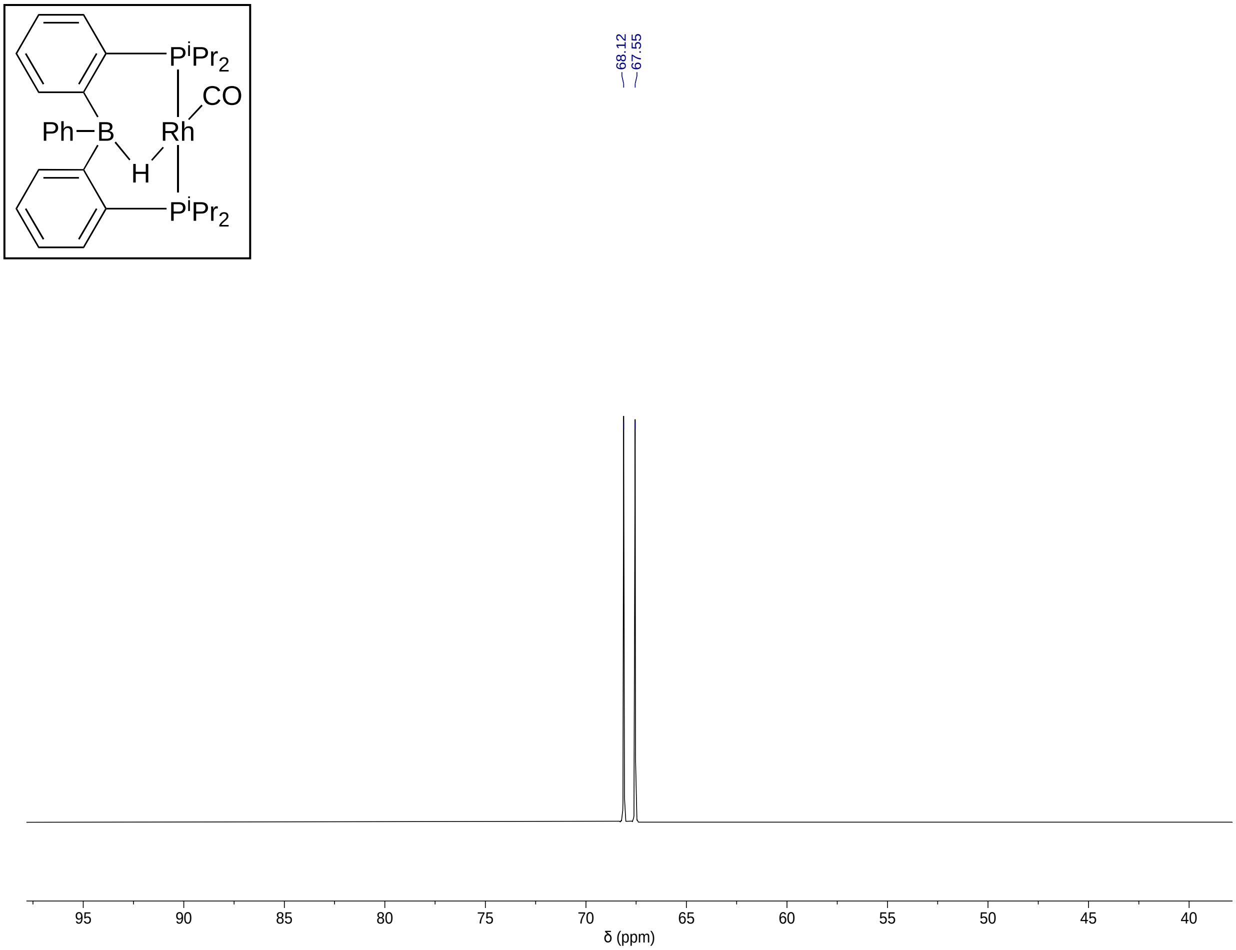

Figure S56. ^{31}P{^{1}H} NMR (202 MHz) spectrum of **3-Rh** in C_6D_6.

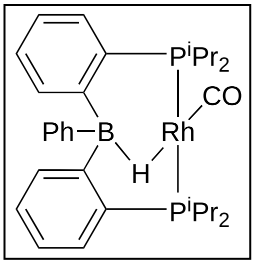

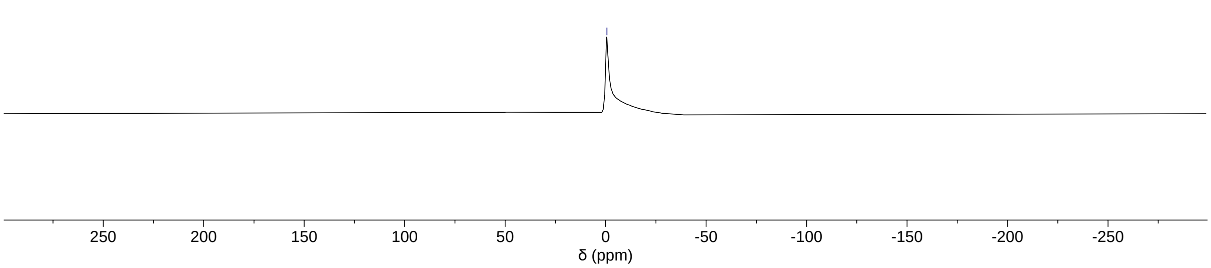

Figure S57. $^{11}B\{^1H\}$ NMR (160 MHz) spectrum of **3-Rh** in C_6D_6.

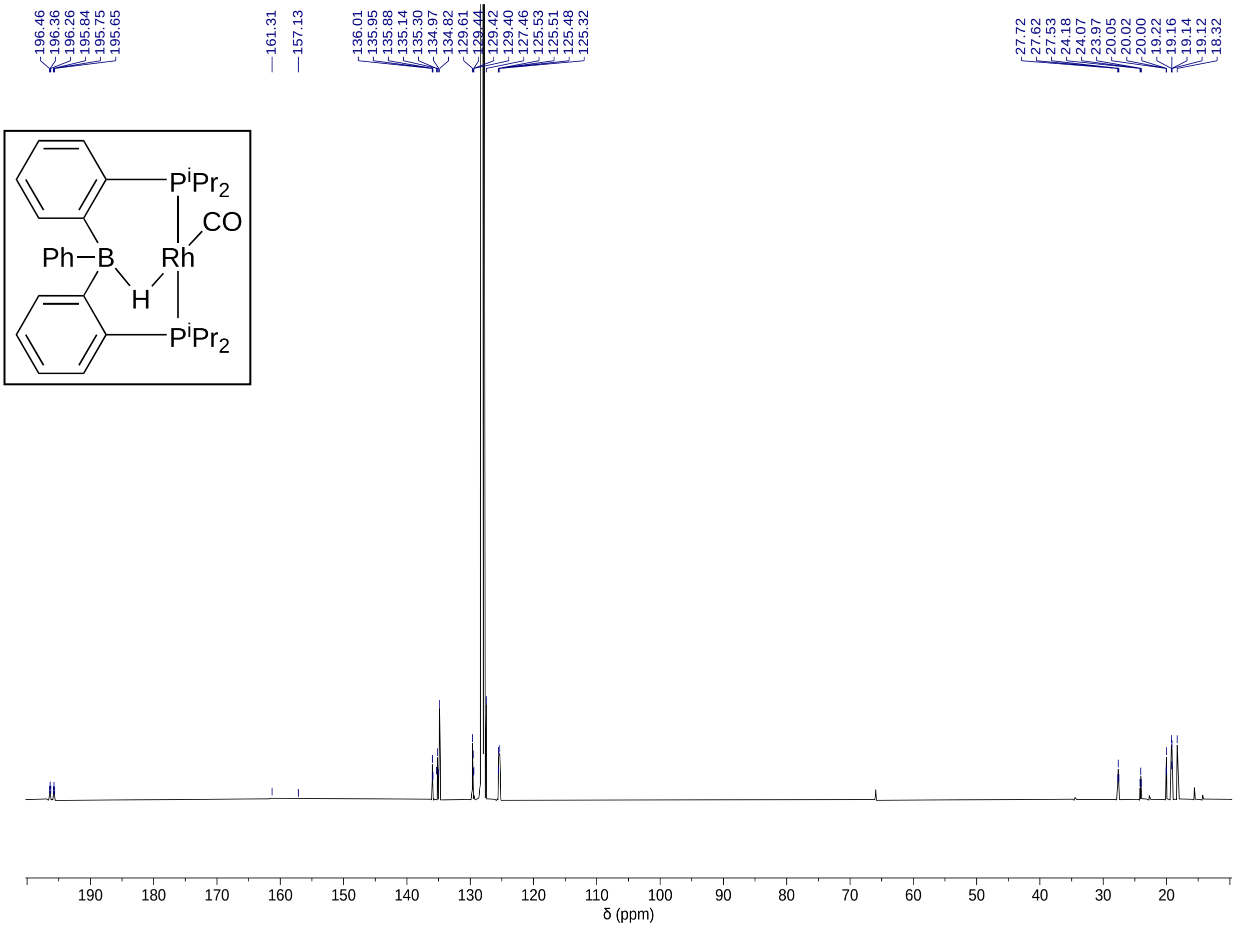

Figure S58. $^{13}C\{^1H\}$ NMR (101 MHz) spectrum of **3-Rh** in C_6D_6.

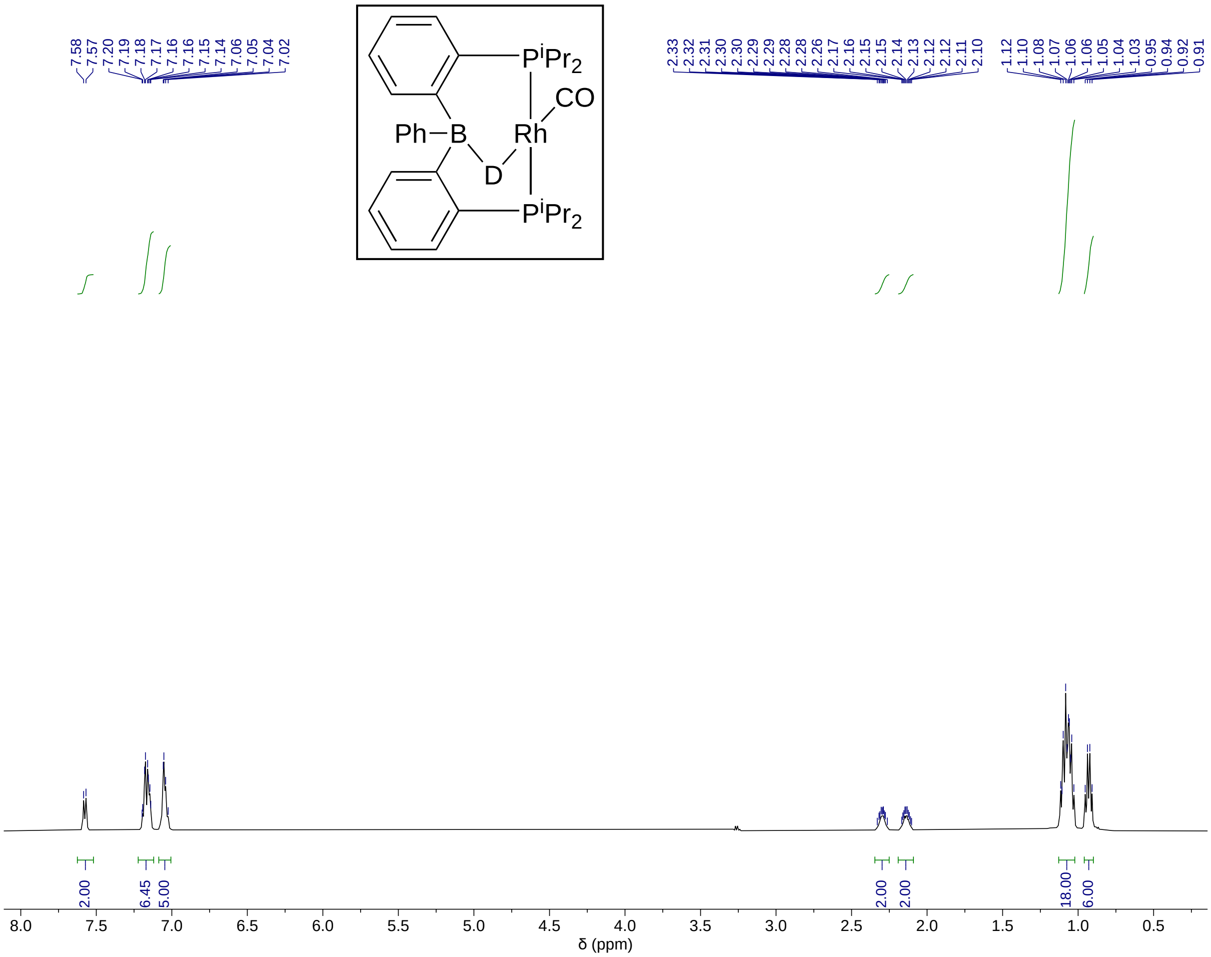

Figure S59. ^{1}H NMR (500 MHz) spectrum of **3-Rh-d** in C_6D_6.

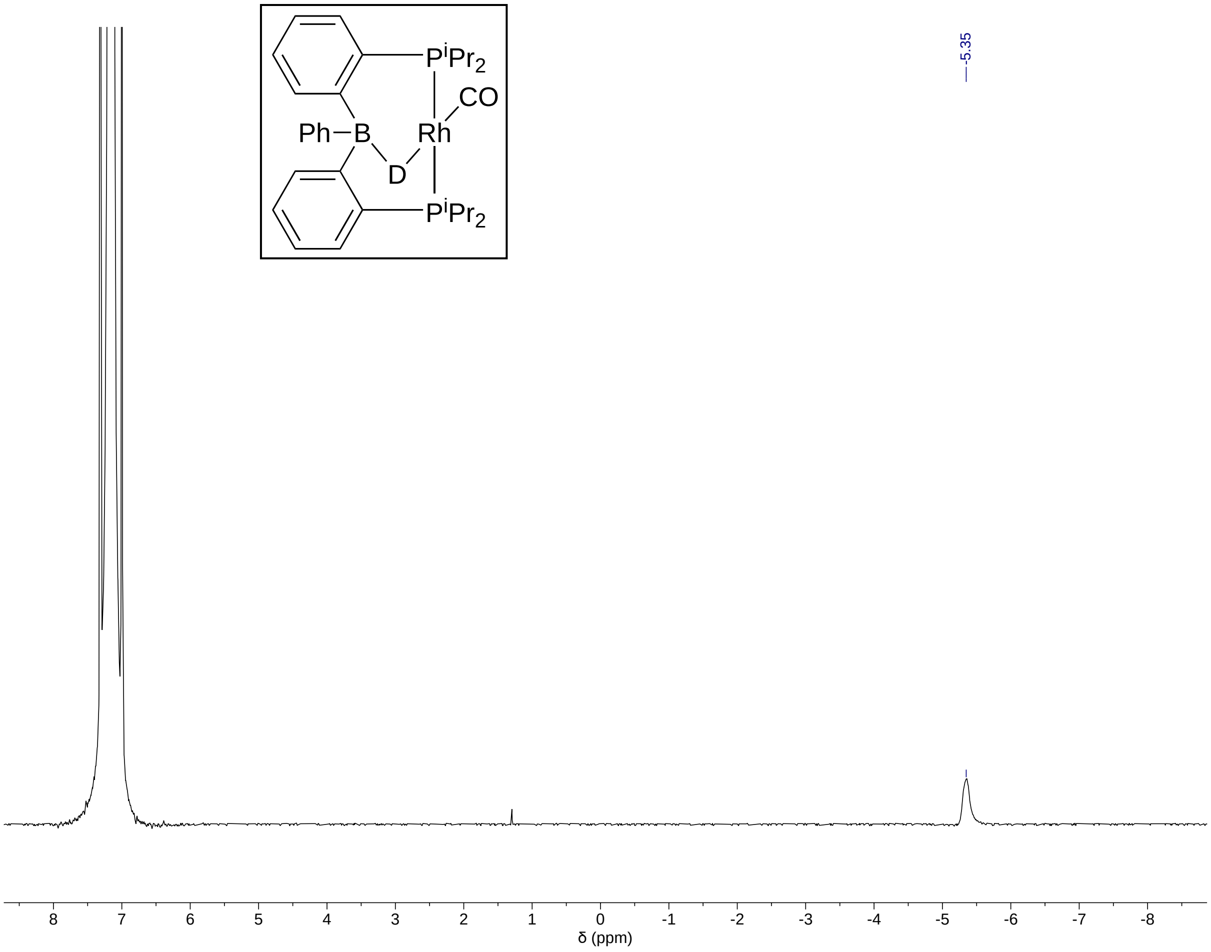

Figure S60. ^{2}H NMR (77 MHz) spectrum of **3-Rh-d** in C_6D_6.

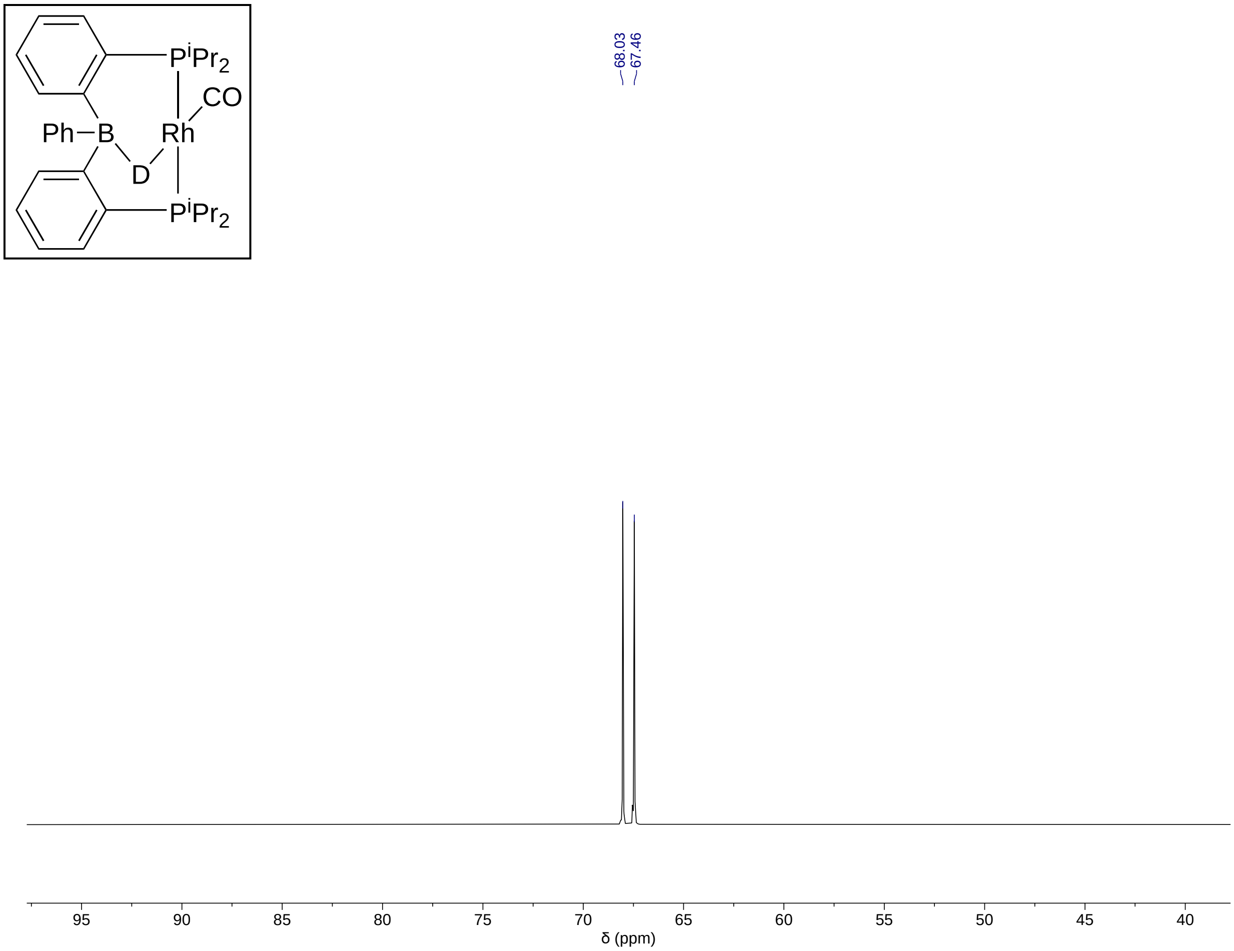

Figure S61. $^{31}P\{^1H\}$ NMR (202 MHz) spectrum of **3-Rh-d** in C_6D_6.

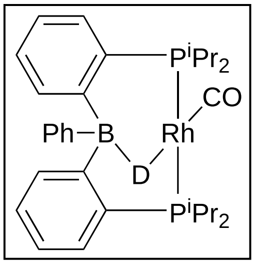

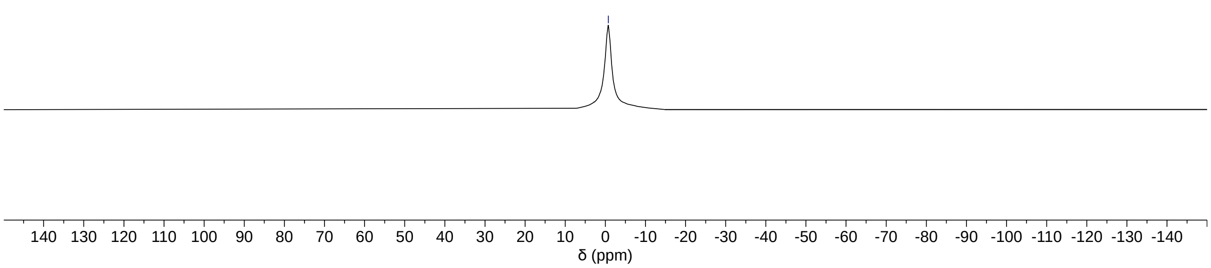

Figure S62. $^{11}B\{^1H\}$ NMR (160 MHz) spectrum of **3-Rh-d** in C_6D_6.

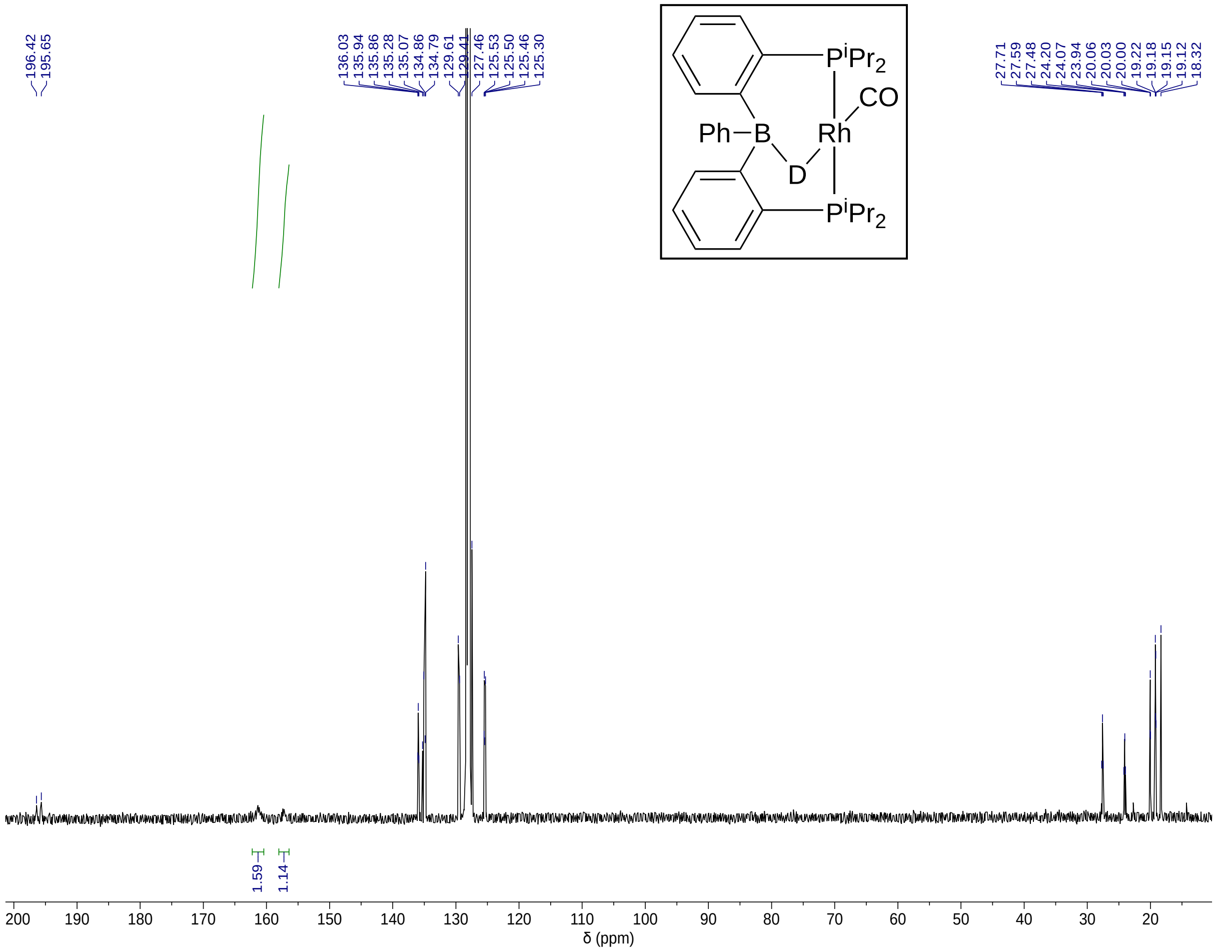

Figure S63. $^{13}C\{^1H\}$ NMR (101 MHz) spectrum of **3-Rh-d** in C_6D_6.

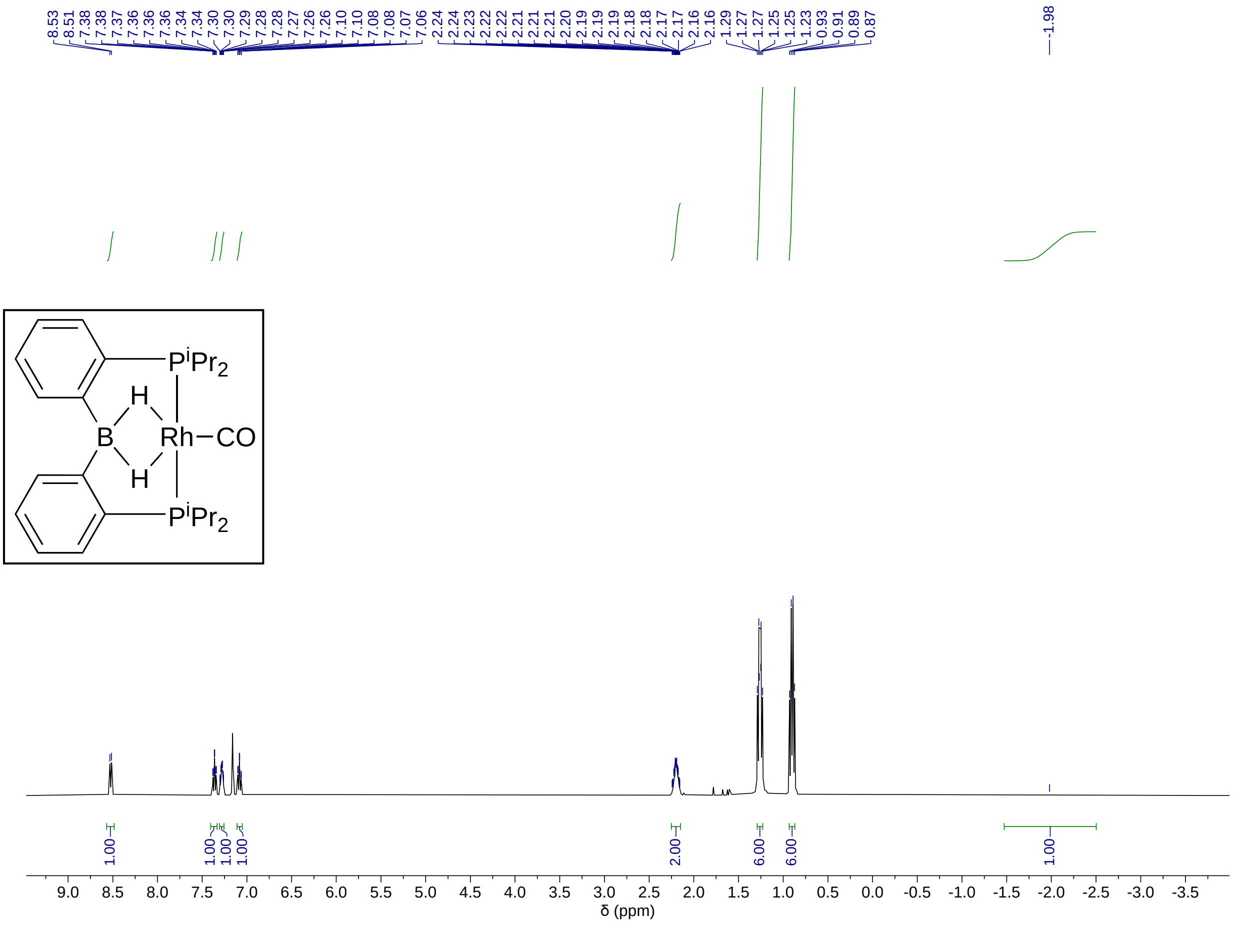

Figure S64. ^{1}H NMR (400 MHz) spectrum of **9-Rh** in C_6D_6.

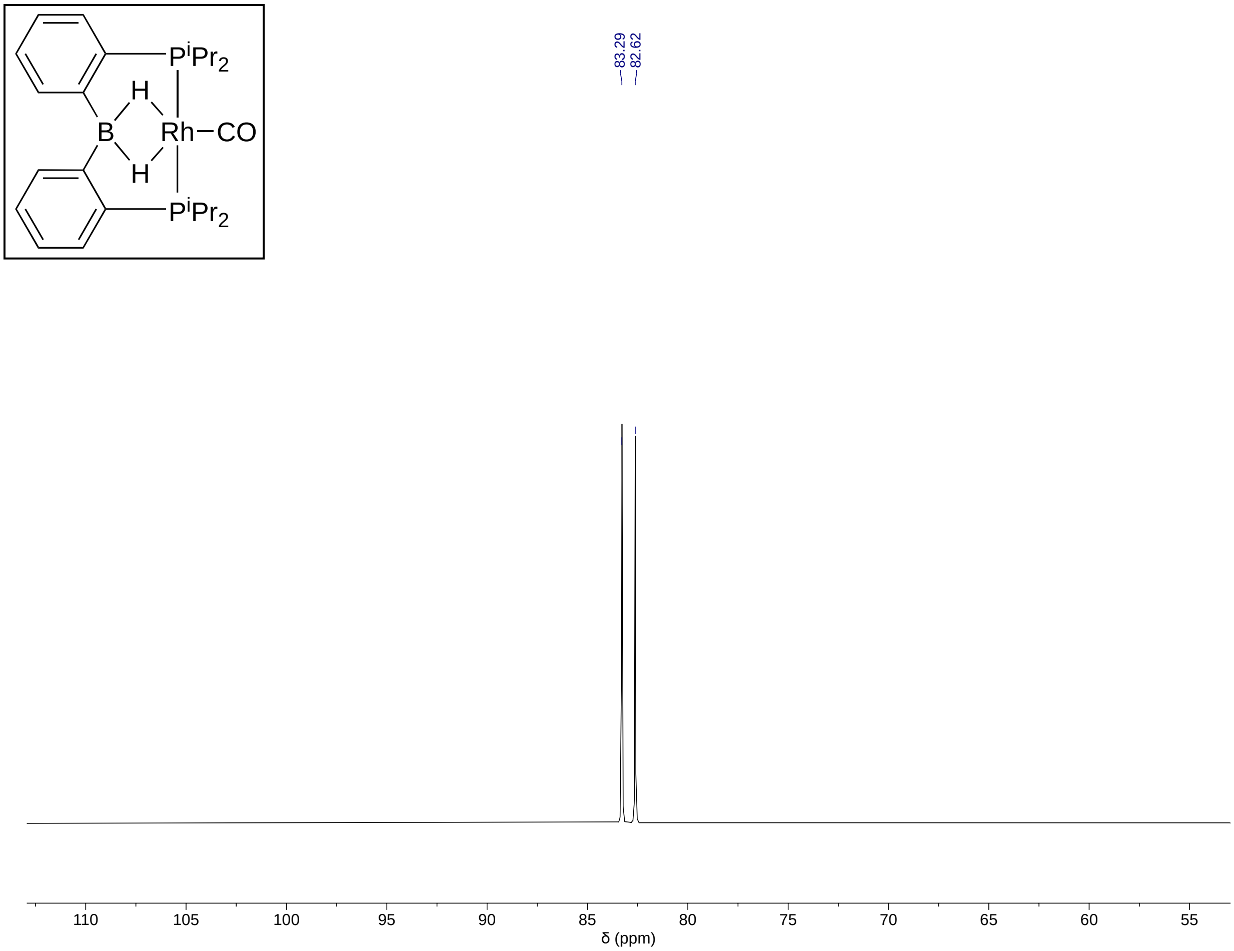

Figure S65. $^{31}P\{^{1}H\}$ NMR (162 MHz) spectrum of **9-Rh** in C_6D_6.

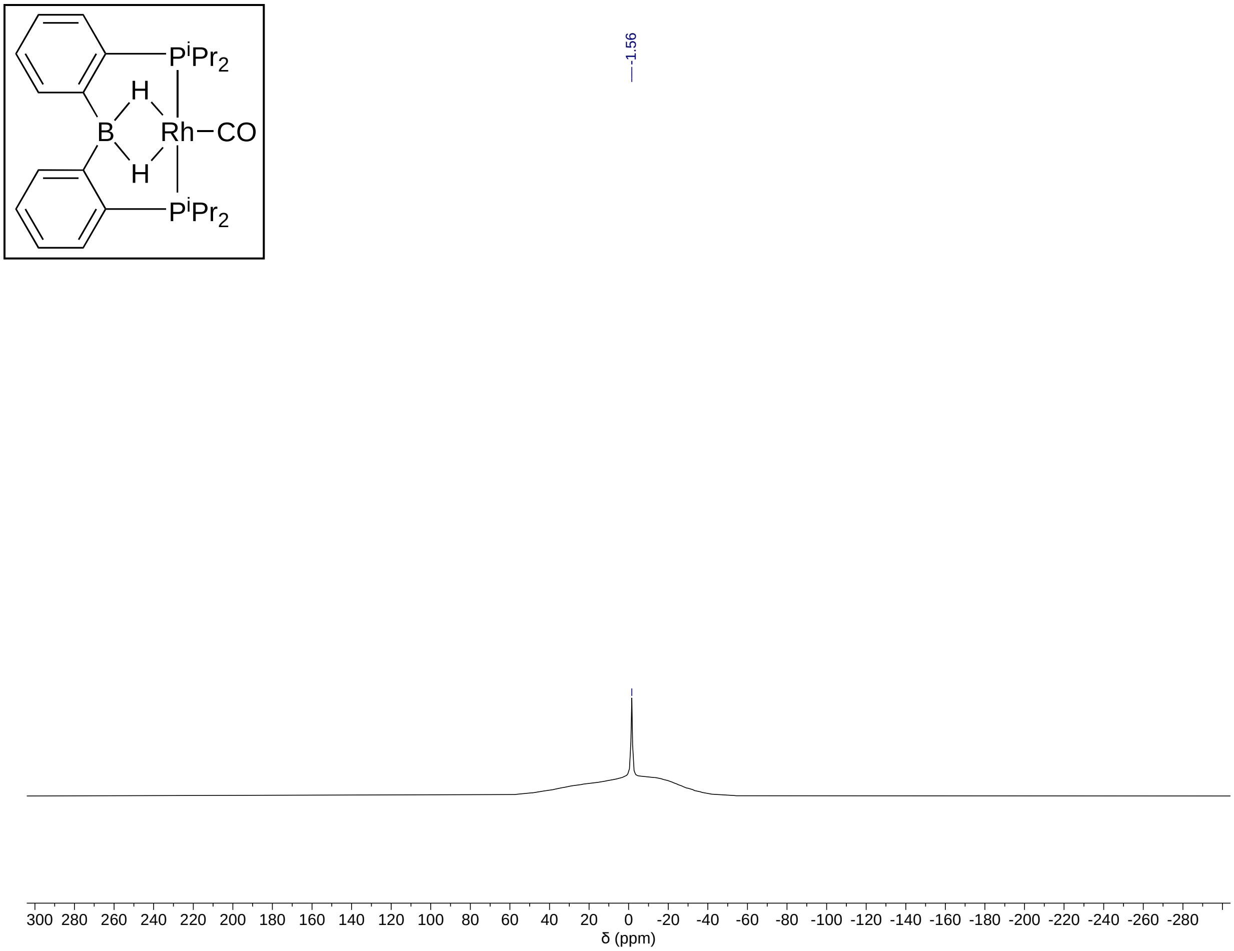

Figure S66. $^{11}B\{^{1}H\}$ NMR (160 MHz) spectrum of **9-Rh** in C_6D_6.

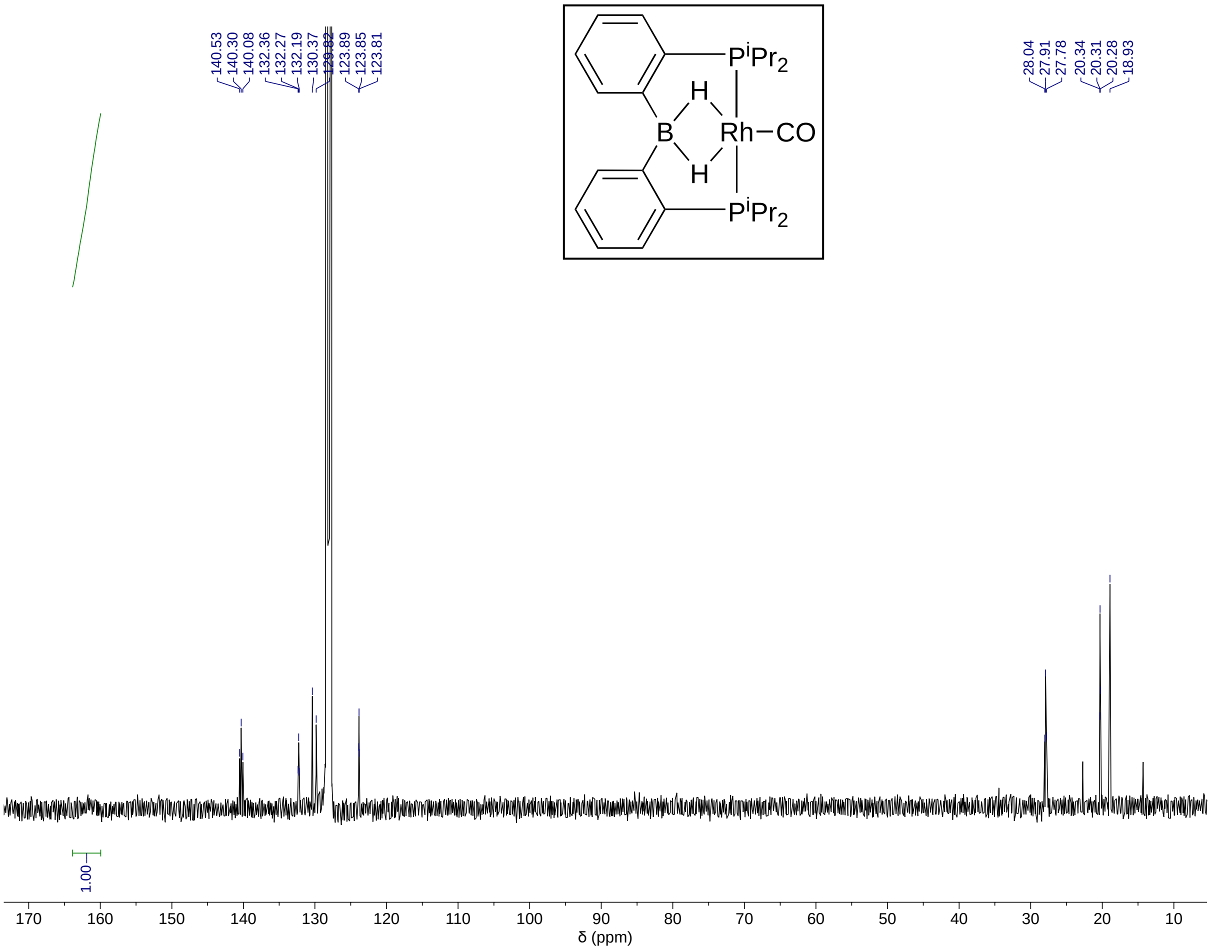

Figure S67. $^{13}C\{^1H\}$ NMR (101 MHz) spectrum of **9-Rh** in C_6D_6.

10. SI References

[1] Shih, W.-C.; Ozerov, O. V. Synthesis and Characterization of PBP Pincer Iridium Complexes and their Application in Alkane Transfer Dehydrogenation. *Organometallics* **2017**, *36*, 228-233.

[2] Shih, W.-C.; Ozerov, O.V. Selective ortho-C-H Activation of Pyridines Directed by the Lewis Acidic Boron of PBP Pincer Iridium Complexes. *J. Am. Chem. Soc.* **2017**, *139*, 17297-17300.

[3] Bontemps, S.; Sircoglou, M.; Bouhadir, G.; Puschmann, H.; Howard, J. A. K.; Dyer, P. W.; Miqueu, K.; Bourissou, D. *Chem. Eur. J.* **2008**, *14*, 731-740.

[4] Cao, Y.; Shih, W.-C.; Bhuvanesh, N.; Zhou, J.; Ozerov, O. V. Cooperative C-H activation of pyridine by PBP complexes of Rh and Ir can lead to bridging 2-pyridyls with different connectivity to the B-M unit, *Chem. Sci.* **2021**, *12*, 14167-14173.

[5] M. J. Frisch, G. W. Trucks, H. B. Schlegel, G. E. Scuseria, M. A. Robb, J. R. Cheeseman, G. Scalmani, V. Barone, B. Mennucci, G. A. Petersson, H. Nakatsuji, M. Caricato, X. Li, H. P. Hratchian, A. F. Izmaylov, J. Bloino, G. Zheng, J. L. Sonnenberg, M. Hada, M. Ehara, K. Toyota, R. Fukuda, J. Hasegawa, M. Ishida, T. Nakajima, Y. Honda, O. Kitao, H. Nakai, T. Vreven, J. A. Montgomery, Jr., J. E. Peralta, F. Ogliaro, M. Bearpark, J. J. Heyd, E. Brothers, K. N. Kudin, V. N. Staroverov, R. Kobayashi, J. Normand, K. Radhavachari, A. Rendell, J. C. Burant, S. S. Iyengar, J. Somasi, M. Cossi, N. Rega, N. J. Millam, M. Klene, J. E. Knox, J. B. Cross, V. Bakken, C. Adamo, J. Jaramillo, R. Gomperts, R. E. Stratmann, O. Yazyev, A. J. Austin, R. Cammi, C. Pomelli, J. W. Ochterski, R. L. Martin, K. Morokuma, V. G. Zakrzewski, G. A. Voth, P. Salvador, J. J. Dannenberg, S. Dapprich, A. D. Daniels, Ö. Farkas, J. B. Foresman, J. V. Ortiz, J. Cioslowski and D. J. Fox, Gaussian 09 (Revision D.01), Gaussian, Inc., Wallingford, CT, 2009.

[6] Grimme, S.; Ehrlich, S.; Goerigk, L. Effect of the Damping Function in Dispersion Corrected Density Functional Theory. *J. Comput. Chem.* **2011**, *32*, 1456-1465.

[7] Marenich, A. V; Cramer, C. J.; Truhlar, D. G. Universal Solvation Model Based on Solute Electron Density and on a Continuum Model of the Solvent Defined by the Bulk Dielectric Constant and Atomic Surface Tensions. *J. Phys. Chem. B* **2009**, *113*, 6378-6396.

[8] CrysAlisPRO, Oxford Diffraction /Agilent Technologies UK Ltd, Yarnton, England.

[9] (a) G. M. Sheldrick, *Acta Cryst.,* 2008, **A64**, 112-122. (b) G. M. Sheldrick, *Acta Cryst.,* 2015, **A71**, 3-8. (c) G. M. Sheldrick, *Acta Cryst.,* 2015, **C71**, 3-8. (d) XT, XS, BRUKER AXS Inc., 5465 East Cheryl Parkway, Madison, WI 53711-5373 USA.

[10] Van Geet, A. L. Calibration of the Methanol and Glycol Nuclear Magnetic Resonance Thermometers with a Static Thermistor Probe. *Anal. Chem.* **1968**, *40*, 2227-2229.

[11] Morse, P.M.; Spencer, M. D.; Wilson, S. R.; Girolami, G. S. A Static α-CH-M Interaction Observable by NMR Spectroscopy: Synthesis of the Chromium(II) Alkyl $[Cr_2(CH_2SiMe_3)_6]^{2-}$ and its Conversion to the Unusual "Windowpane" Bis(metallacycle) Complex $[Cr(\kappa^2 C,C'-CH_2SiMe_2CH_2)_2]^{2-}$. *Organometallics* **1994**, *13*, 1646-1655.
